# Supplementary figures and images for: The effect of protecting women against economic shocks to fight HIV in Cameroon, Africa: The POWER randomised controlled trial
Source: PLoS Med. 2024 Oct 24;21(10):e1004355. doi: 10.1371/journal.pmed.1004355 (PMC11500901; doi:10.1371/journal.pmed.1004355)

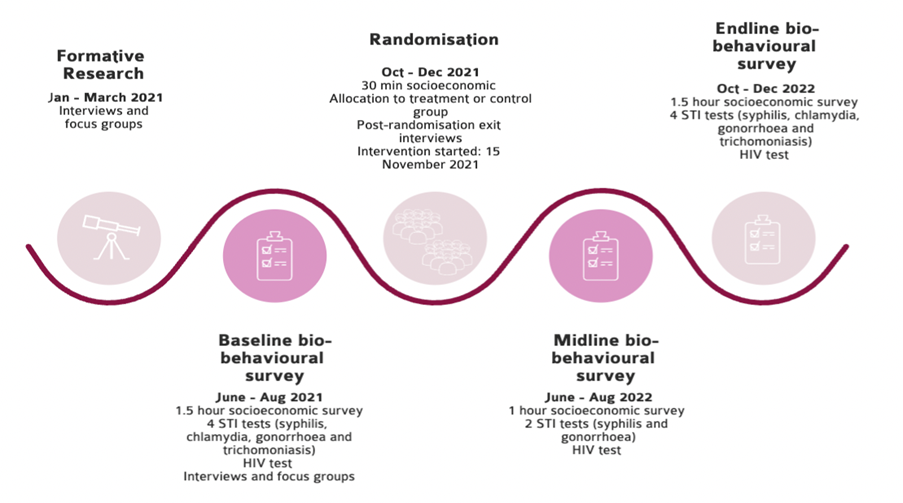

Supplement: S1 Fig — (TIF) [file pmed.1004355.s002.tif]

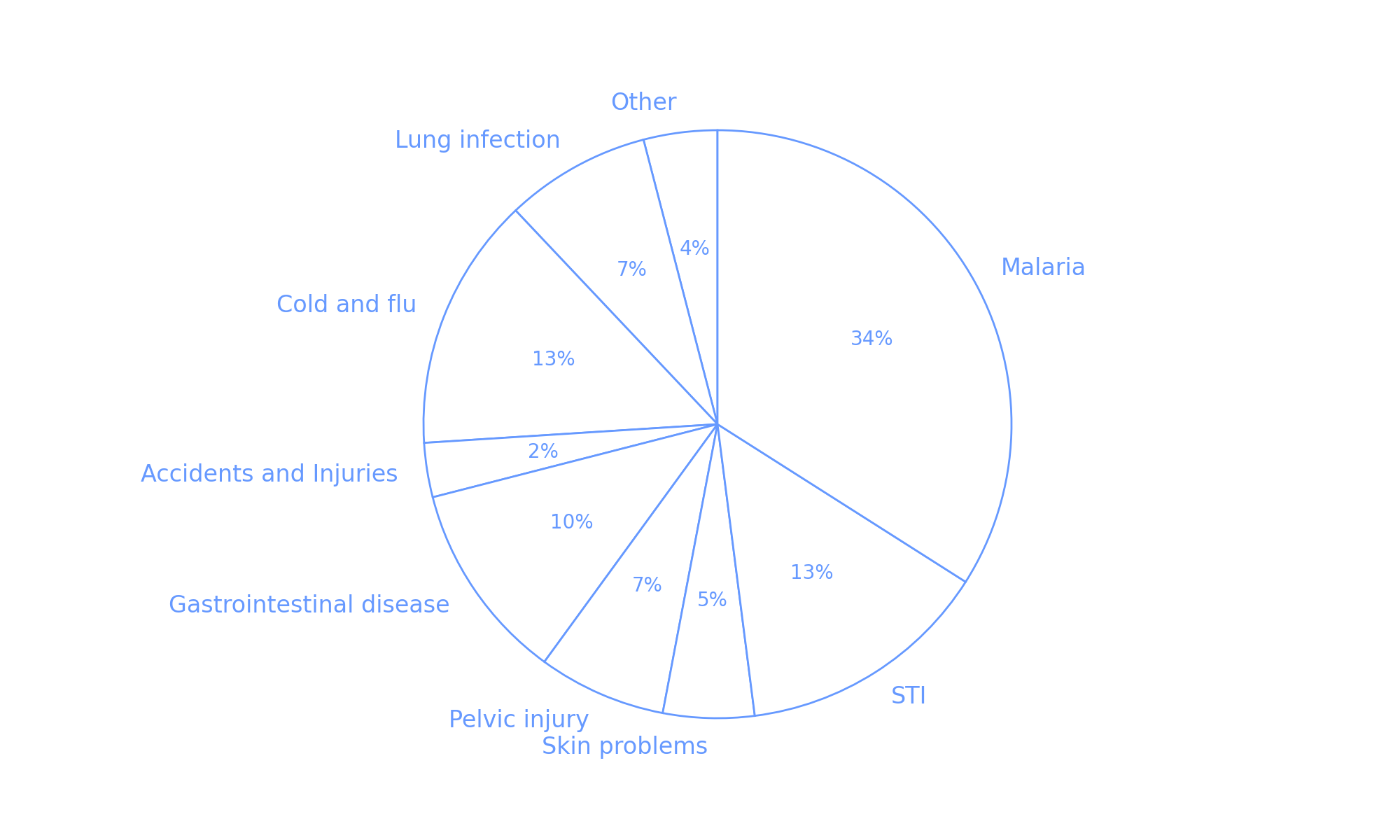

Supplement: S2 Fig — (TIF) [file pmed.1004355.s003.tif]
