# Supplementary material for: The effect of protecting women against economic shocks to fight HIV in Cameroon, Africa: The POWER randomised controlled trial
Source: PLoS Med. 2024 Oct 24;21(10):e1004355. doi: 10.1371/journal.pmed.1004355 (PMC11500901; doi:10.1371/journal.pmed.1004355)
Supplement: S1 Appendix — (DOCX) [file pmed.1004355.s004.docx]

Appendix A: List experiment method and double list experiment design

Our primary measure of risky sexual behaviour within sex acts is condom use collected via the verified double list experiment method, an indirect elicitation method. The advantage of the list experiment is that it allows respondents in our survey to answer sensitive questions, in our case about the use of condoms during their last sex act, allowing confidentiality since the enumerator or researchers are not able to assign the behaviour to a specific individual. It allows a more accurate prevalence of condomless sex to be estimated, minimising social desirability bias. Previously, the list experiment method has been used for eliciting abortion (Moseson et al., 2021; Bell and Bishai, 2019), voting preferences (Gonzalez-Ocantos et al., 2012; Holbrook and Krosnick, 2010), use of micro-finance loans (Karlan and Zinman, 2012), opinions on undocumented migrants (McKenzie and Siegel, 2013), gay marriage (Lax et al., 2016) and racism (Krumpal, 2013) and has been proven to be effective to measure condom use (LaBrie and Earleywine, 2000; Treibich and Lépine, 2019). There is debate over the effectiveness of the list experiment in measuring sensitive behaviours. Lensvelt-Mulders et al. (2016) performed a meta-analysis finding it more accurate than face-to-face questioning at estimating prevalence of sensitive behaviours, whereas several other studies find issues often resulting from a poor list experiment design, including respondents’ understanding is high (Haber et al., 2018) and Imai (2012) and Imai (2011).

Here we describe the statements for the women engaging in transactional sex, but the method is identical for the commercial sex group. During the survey, when an enumerator reaches the list experiment question, their respondent is randomly allocated to the treatment or control groups for the list experiment and asked how many of the following statements the respondent agrees with. It then lists either three non-sensitive statements for the control group:

- Usually, I meet my sugar daddies on the street.
- My sugar daddy is older than me.
- Monday is the day I see most of my sugar daddies.

Or for the treatment group, it lists the same three non-sensitive statements plus a sensitive statement of interest in position 2:

- Usually, I meet my sugar daddies on the street.
- **I used a condom the last time I had sex with my last sugar daddy**.
- My sugar daddy is older than me.
- Monday is the day I see most of my sugar daddies.

The key assumption is that the average number of non-sensitive statements agreed with is the same for the treatment and control groups. Therefore, the difference in the average number of statements agreed with is the prevalence of condom use at the last sex act.

The double list experiment method simply repeats the list experiment with a new set of non-sensitive statements and reverses the treatment and control groups allocated in the first experiment. This means each respondent receives the sensitive statement at least once during the interview. The second set of non-sensitive statements are:

- I like to look good before I go out.
- I consider myself as a sex worker.
- I want to marry my sugar daddy.

The prevalence can also be estimated using OLS regression analysis. When using the double list experiment, each respondent appears in the model as two observations, one when they were in the control group and one in the treatment group of the list experiment.

As you can see, the advantage of this method is that there is no way for the researcher to back out the true answer to the sensitive statement that a respondent has, providing privacy to answer in confidence. This strength is also a drawback since interpretation of findings can only be made about a groups prevalence and not at the individual level. The validity of the list experiment has been examined and proven elsewhere [39,47].

For the commercial sex strata the two sets of non-sensitive statements are:

- Usually, I meet my clients at the couloir.
- I prefer the client to pay me before the act.
- Monday is the day I have the most clients.

And for the group that receives the sensitive statement:

- I like to look good before I go out.
- **I used a condom the last time I had sex with my last client.**
- I like all of my clients.
- Usually, I solicit clients by phone.

The prevalence can almost be estimated using simple OLS regression that can also be used to estimate the difference in the prevalence between sub-groups within our dataset. To determine the impact of the intervention on condom use collected via the list experiment, we estimate the following equation:

$$Y_{i}^{LE}=\beta_{0}+\beta_{1}{LT}_{i}+\beta_{2}S_{i}+\beta_{3}S_{i}*{LT}_{i}+\beta_{4}{List}_{i}+\beta_{x}X_{i}+\beta_{z}X_{i}*{LT}_{z}+u_{i}$$

Where $Y$ indicates the number of statements the individual *i,* agreed with during the list experiment (*LE*). $LT$ indicates if the individual received the treatment or control list, $S$ indicates if the respondent was part of the treatment group or not. Since we are using the validated double list experiment where each individual is asked the list experiment twice, once as a treatment list and once as a control list, the term $List$ is an indicator for which set of non-sensitive statements they received. $X$ is a list of individual controls. Models are estimated with robust standard errors clustered at the individual since each respondent is included twice within each model, once for the first instance of the list experiment and once for the second instance. $\beta_{3}$ is our parameter of interest and represents the difference in condom use between the treated and control groups or the marginal impact of the intervention on condom use at the last sex act.

Appendix B: Difference between baseline characteristics of those lost to follow up in the transactional sex strata

Appendix C: Difference between baseline characteristics of those lost to follow up in the commercial sex strata

Appendix D: Difference between baseline characteristics of those lost to follow up in the transactional sex strata

Appendix E: Effect of the intervention on HIV and STI (odds ratios)


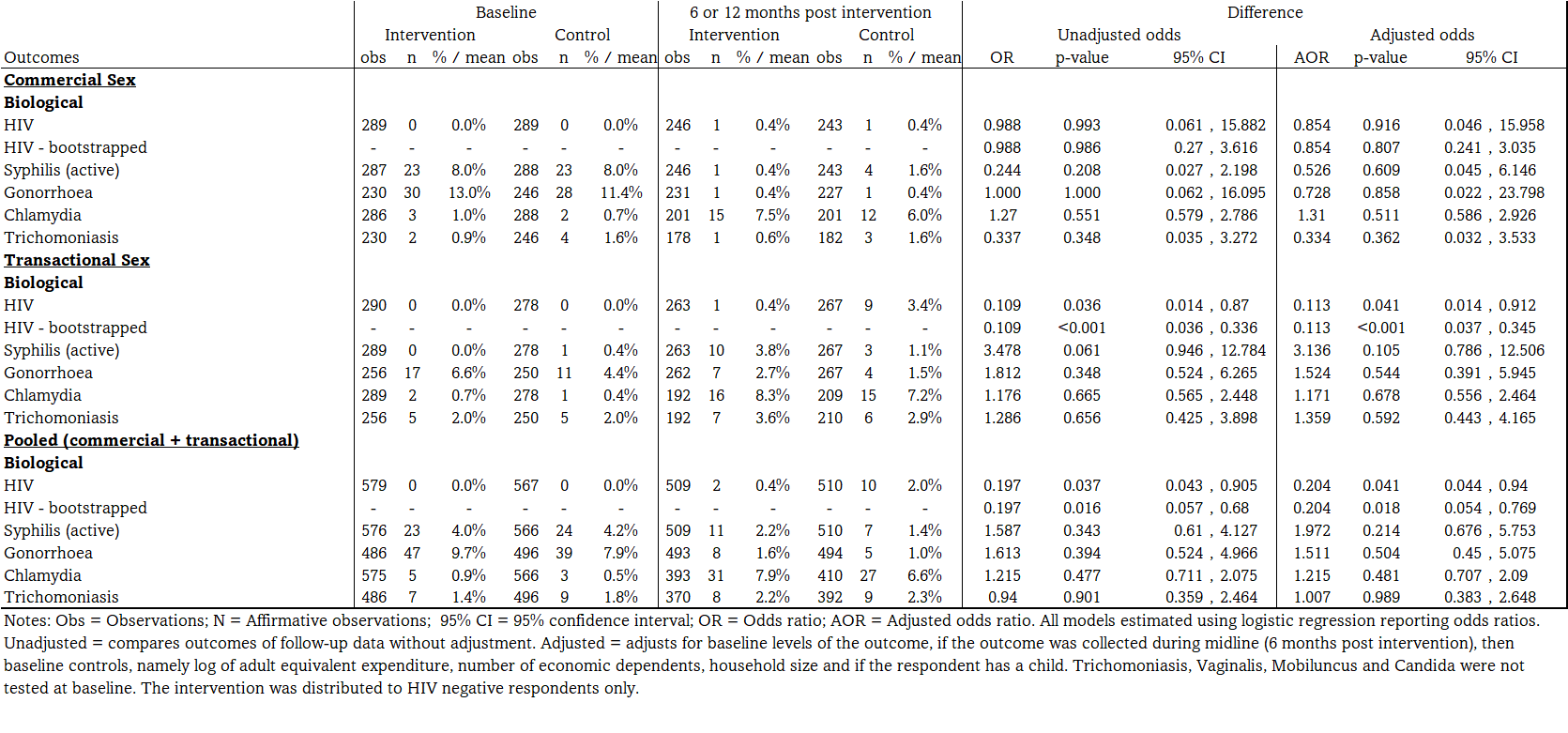


Appendix F: Effect of the intervention on HIV and STI (marginal effects)


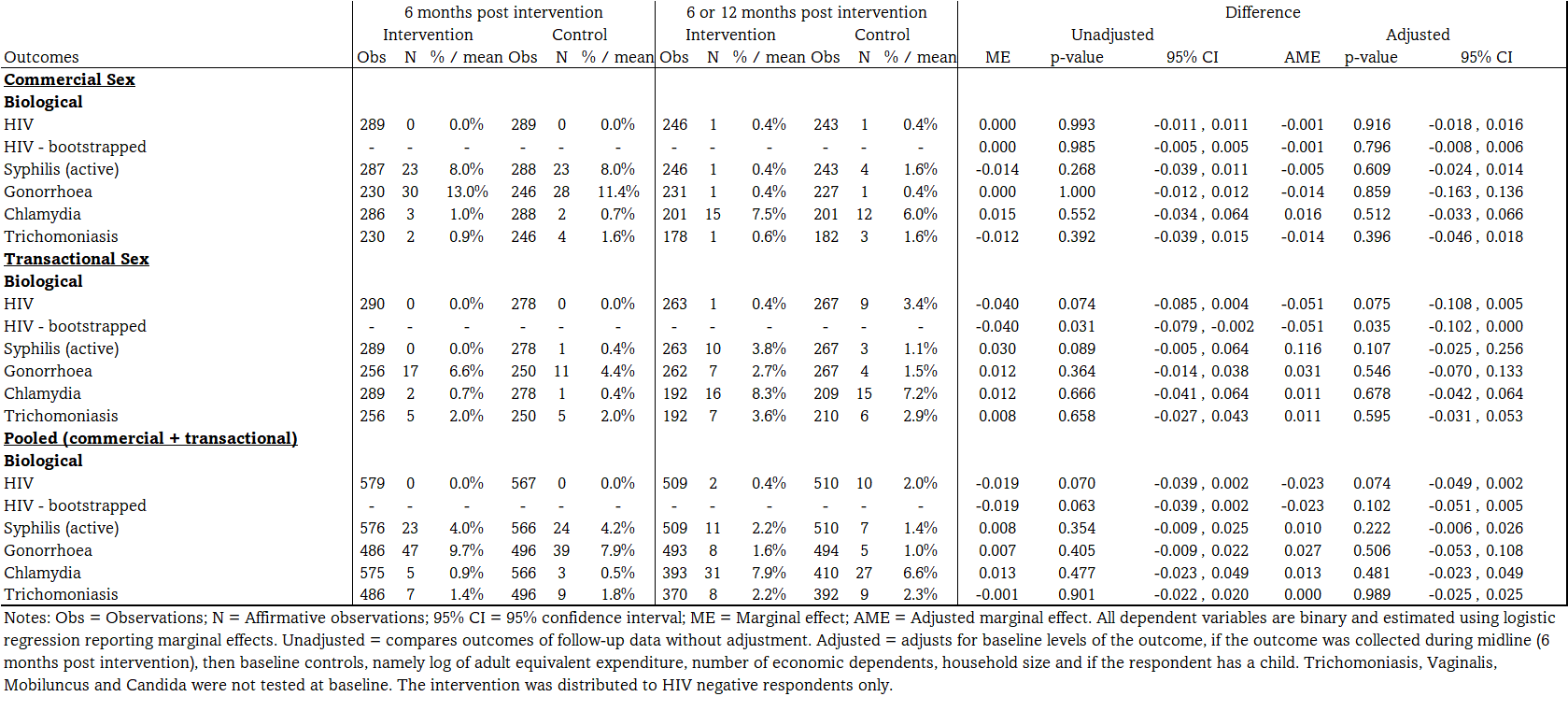


Appendix G: HIV and self-reported STI symptoms for women in the transactional sex strata

Appendix H: STI measurement issues

Below is more detail on the lack of effect found for STIs other than HIV. For gonorrhoea and trichomoniasis, the tests required vaginal swabs, which are difficult to collect [48], and testing in the laboratory, which meant that there was a high refusal rate (gonorrhoea and trichomoniasis refusal rates were 17% at baseline, 14% at 6 months post-intervention and 10% at 12 months post-intervention, compared with zero refusals for HIV across all surveys). We found that there were problems with transport and storage in hot and humid environments, which affected the accuracy of the tests because samples had to be kept cold [30]. We found zero cases of gonorrhoea at 12 months post-intervention, but an average prevalence of 9.2% at 6 months post-intervention. Trichomoniasis results did not differ between 6 and 12 months post-intervention, but as the samples tested were the same as those for gonorrhoea, they suffered from the same refusal and transport problems, limiting their validity.During the baseline and 6 months post-intervention surveys, every 15th chlamydia, syphilis and HIV test performed (including HIV) and all inconclusive and positive tests were sent to a laboratory for verification using the same rapid test as performed in the field. We found that many of the positive tests taken during the survey came back negative from the laboratory. Therefore, at 12 months post-intervention, retesting was moved to the ELISA laboratory, where a new chlamydia test and a more robust syphilis test were performed. We then found that many of the 15th sample retests were coming back positive after a negative rapid test. Following this finding, we sent all chlamydia and syphilis samples for laboratory testing as well as completing the rapid tests at the survey site. This led to our findings of poor sensitivity and specificity of the chlamydia and syphilis tests currently recommended by the Cameroonian government.

Appendix I: Odds ratio of behavioural outcomes & secondary outcomes


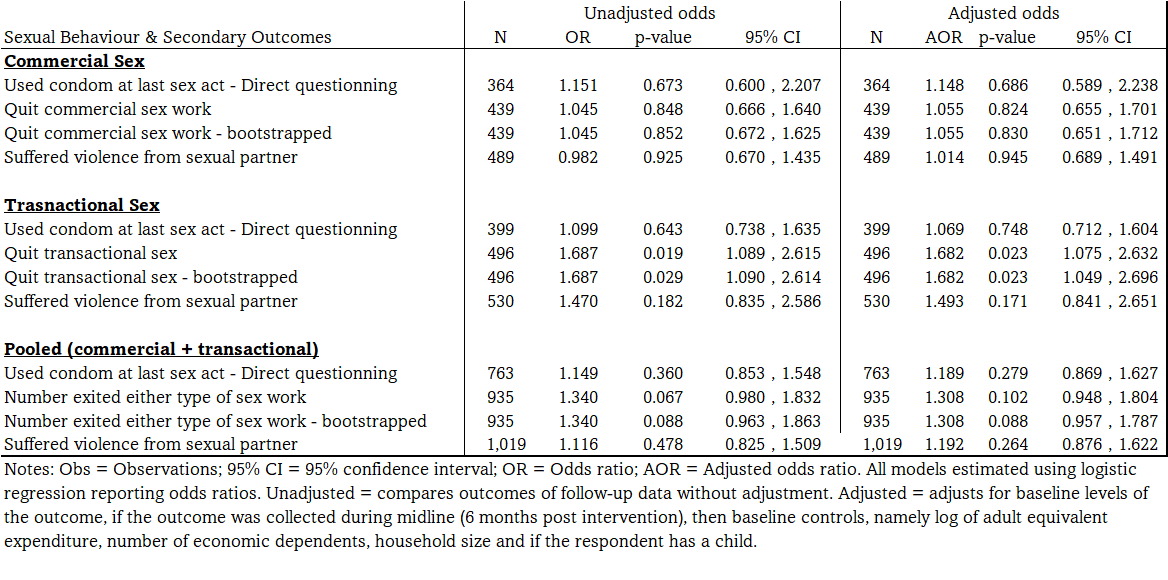


Appendix J: Effect of health insurance on HIV for those engaged in transactional sex accounting for STI symptoms

The impact of the intervention on HIV for women in transactional sex can work through two channels, see Figure 1. First, through protection against health care spending related economic shocks that reduce the incentives to engage in risky behaviours resulting in less risky sex; second, through treatment for STIs and therefore lower susceptibility to HIV that way [37]. To somewhat disentangle these two mechanisms, we can examine the role of self-reported STI symptoms in mediating the intervention’s impact on HIV in the transactional arm.

Another method to check the channel the intervention works is to examine those who are expected to benefit more or less from the intervention. Whilst predicting illness is impossible, we know that those with more economic dependents will have a greater chance of both suffering an illness and receiving free care for the illness. Therefore, we test whether the intervention impacted condom use (using the list experiment) for those with more or less than three economic dependents finding the intervention leads to a 25% increase in condom use (ME=0.248 (95% CI[0.031,0.466]); p=0.026) for women in transactional sex with more than three economic dependents but has no effect (ME=-0.067 (95% CI[-0.34,0.212]); p=0.638) for those with less than three economic dependents^^[[1]](#footnote-1)^.^ Together this evidence suggests the primary mechanism at work is that of protection against economic shocks rather than through treatment of STIs.


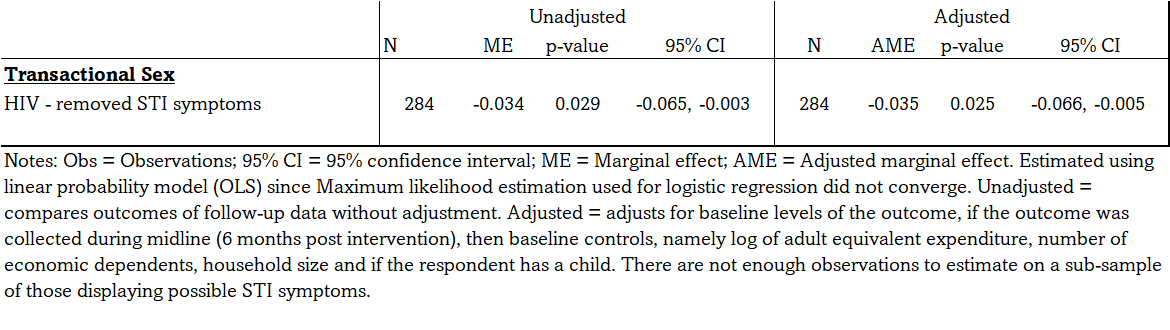


Appendix K: Effect of health insurance on moral hazard


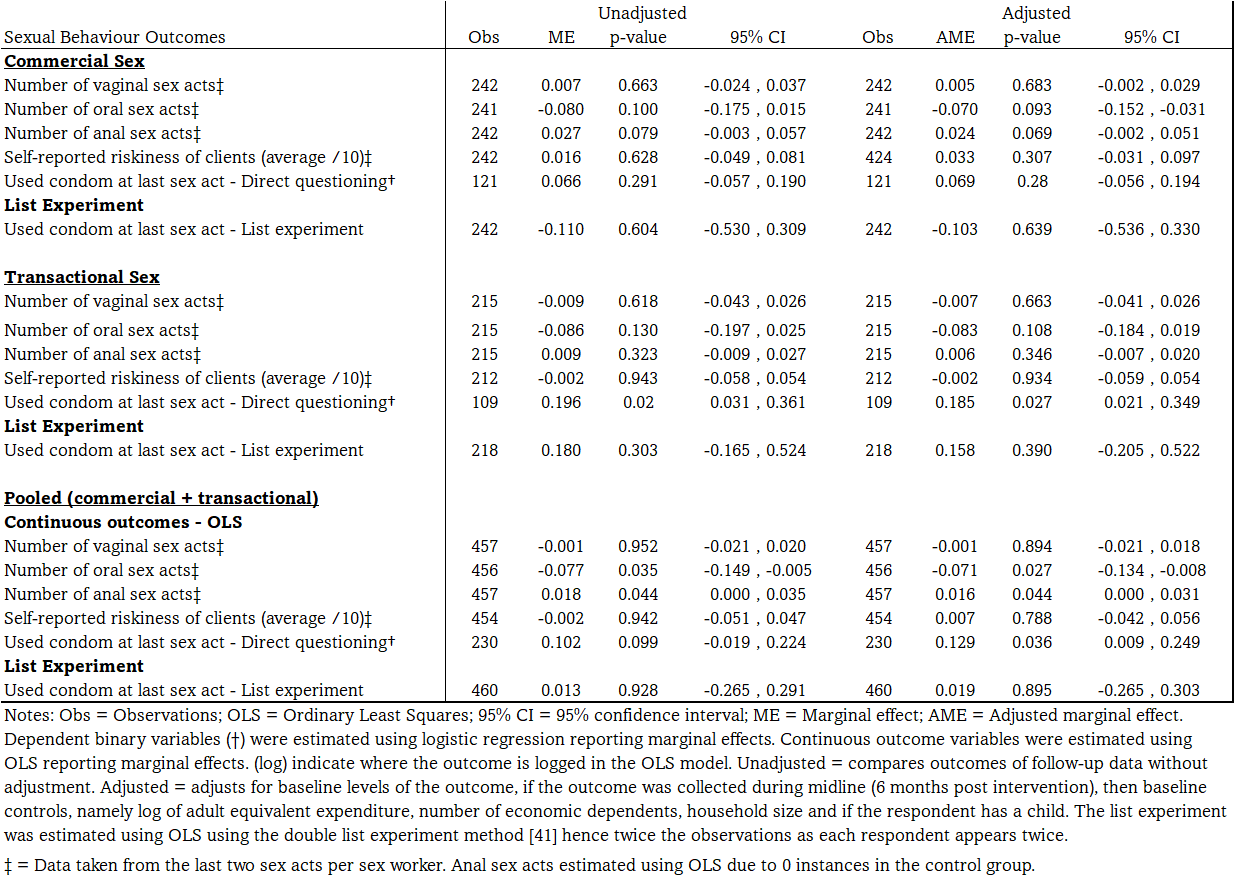


The intervention succeeded in its primary aims of increasing the likelihood of reducing the costs of seeking health care when respondents or economic dependents were sick or injured. The likelihood of suffering a health-related economic shock also fell alongside typical and last 30-day health-related out-of-pocket payments, see Table 5. Moral hazard could counter the reductions in risk through protection from the intervention. Ex-ante recipients could increase risky health behaviours because of health insurance provision, or ex-post are more likely to use more healthcare than needed after an accident or illness occurs. The latter is difficult to measure and is not relevant to our mechanism. However, increased risk-taking behaviour in the knowledge treatment is provided for free could counteract reduction in risks from shocks protection.

Table 5 shows no difference in reported illness between intervention and control groups at follow-up, suggesting little moral hazard in reporting of illness or increased risk-taking leading to illness overall. We also test moral hazard by comparing risky behaviours of those who do not report an illness or injury to themselves or an economic dependent; since they did not need the financial protection, any differences are likely to suggest moral hazard is occurring. Appendix K shows an increase anal sex (ME=0.016 (95% CI[0.000,0.031]); p=0.044, for pooled sample) but reductions in other risky behaviours for those for those in the intervention group but that did not use it. The intervention reduces risky behaviours in these people with protected sex increasing (ME=0.129 (95% CI[0.009,0.249]); p=0.036 for pooled sample), and oral sex acts falling (ME=-0.071 (95% CI[-0.134,-0.008]); p=0.027, for pooled sample) among women who did not report any illness.

1. Not reported in tables. [↑](#footnote-ref-1)
