# Supplementary material for: The effect of protecting women against economic shocks to fight HIV in Cameroon, Africa: The POWER randomised controlled trial
Source: PLoS Med. 2024 Oct 24;21(10):e1004355. doi: 10.1371/journal.pmed.1004355 (PMC11500901; doi:10.1371/journal.pmed.1004355)
Supplement: S1 Study Protocol — (DOCX) [file pmed.1004355.s006.docx]

Title: **Protecting women from economic shocks to fight HIV in Africa (POWER): A study protocol for a randomised controlled trial**

Authors: Sandie Szawlowski^1^, Emile Nitcheu^2^, Eric Defo Tamgno^2^, Fanny Procureur^1^, Julienne Noo^2^, Stephanie Moyoum^2^, Illiassou Mfochive^2^, Serge Billong^3^, Ubald Tamoufe^2^, Aurélia Lépine^1^

Affiliation:

1. Institute for Global Health, University College London, London, United Kingdom
2. John Hopkins Cameroon Programme, Yaoundé, Cameroon
3. University of Yaoundé I, Yaoundé, Cameroon

Corresponding author: Aurélia Lépine, Institute for Global Health, University College London, United Kingdom, [a.lepine@ucl.ac.uk](mailto:a.lepine@ucl.ac.uk)

Contributorship: SS led the drafting of the manuscript and contributed to the design of the study. FP contributed to the design of the study drafting of the manuscript and gave final approval of the manuscript. ED, SB, IMN and UT contributed to the design of the study and gave final approval of the manuscript. AL conceived of and led the design of the study, led the proposal and the protocol development, contributed to the drafting of the manuscript and gave the final approval of the manuscript. All authors read and approved the final manuscript.

**Title**

Protecting women from economic shocks to fight HIV in Africa (POWER): A study protocol for a randomised controlled trial

**Abstract**

**Introduction:** HIV/AIDS is the leading cause of mortality among women aged 15 – 44 years. In Africa is there a huge gender disparity in the acquisition of HIV. African women aged 15 – 24 are twice as likely to be infected with HIV than their male counterparts. However, in Cameroon, adolescent girls are five times more likely to be infected with HIV than boys of the same age. Evidence suggests that risks taken during transactional and commercial sexual relationships account for this gender inequality. Recent studies demonstrate that a key driver of a woman’s engagement in risky sexual behaviours is to cope with negative income shocks (e.g., agricultural and climatic shocks, illness or death of family members), suggesting that economic shocks are a substantial piece of the HIV puzzle in Africa. This study is the first to estimate the effectiveness of a formal coping strategy, health insurance, to protect women from economic shocks to prevent STIs and HIV.

**Methods and Analysis**: This study is a stratified cluster randomised controlled superiority trial with two parallel groups. 1,508 unmarried girls and young women engaging in transactional (753) and commercial (755) sex have been recruited onto a baseline study in Yaoundé, Cameroon. Participants are randomised into treatment (receiving health insurance for them and their economic dependents) and control (receiving nothing) groups using participatory randomisation. A target of a 1:1 allocation was stratified by those who engage in commercial or transactional sex. Midline data will be collected at 6 months and 12 months after randomisation.

**Ethics and Dissemination:** This trial has been prospectively registered with the ISRCTN Registry. We obtained ethical approval from Cameroon National Ethics Committee and the UCL ethics committee before data collection. The findings of the trial will be published in peer-reviewed journals irrespective of the final results.

**Trial registration**:

ISRCTN 22516548. Registered on 31 July 2021.

**Funding:**

This project is funded by Dr Aurélia Lépine’s (PI) UKRI Future Leaders Fellowship (round 2, 2020-2024) which is an award by the MRC/ESRC (grant number: MR/S031790/1).

**Keywords**

Randomised controlled trial; health insurance; vulnerable women; STIs/HIV

**Administrative information**

| **Title** | Protecting women from economic shocks to fight HIV in Africa (POWER): A study protocol for a randomised controlled trial |
| --- | --- |
| **Trial registration** | ISRCTN 22516548. Registered on 27 May 2021. |
| **Protocol version** | POWER protocol version 1 |
| **Funding** | This project is funded by Dr Aurélia Lépine’s (PI) UKRI Future Leaders Fellowship (round 2, 2020-2024) which is award by the MRC/ESRC (grant number: MR/S031790/1). |
| **Author details** | 1. Ms Sandie Szawlowski, University College London 2. Mr Emile Nitcheu, John Hopkins Cameroon Programme 3. Mr Eric Defo Tamgno, John Hopkins Cameroon Programme 4. Ms Fanny Procureur, University College London 5. Ms Julienne Noo, John Hopkins Cameroon Programme 6. Ms Stephanie Moyoum, John Hopkins Cameroon Programme 7. Dr Iliassou Mfochive, John Hopkins Cameroon Programme 8. Dr Serge Billong, University of Yaoundé I 9. Mr Ubald Tamoufe, John Hopkins Cameroon Program 10. Dr Aurélia Lépine, University College London |
| **Name and contact information for the trial sponsor** | UK Research and Innovation 58 Victoria Embankment London EC4Y 0DS |
| **Role of sponsor** | The funding source is a UKRI Future Leaders Fellowship awarded to Dr Lépine, UKRI had no role in the design of this study and will not have any role in any of these processes. |

**Introduction**

**Background and rationale {6a}**

Human immunodeficiency virus (HIV)/ Acquired immune deficiency syndrome (AIDS) is one of the leading causes of mortality globally and is the leading cause among women aged 15 – 44 years. African women aged 15 – 24 are twice as likely to be infected with HIV than their male counterparts (1). In Cameroon, one of the countries with the highest gender disparity in HIV globally, adolescent girls are five times as likely to be infected with HIV than boys of the same age. Sexually transmitted infections (STIs) and HIV not only affect women and their sexual partners, but they are also a special concern during pregnancy and pose serious health risks to unborn children (2). Hence, reducing STI and HIV acquisition in high-risk women can translate into much wider societal benefits and targeted interventions have the potential to provide excellent value for money. There is a growing number of studies showing that risks taken during transactional sex (non-commercial sexual relationships in exchange for material support or other benefits) and commercial sex – in addition to biological susceptibility – are responsible for gender inequalities in HIV/AIDS. However, there is a superficial understanding of the main causes driving risky sexual behaviours of women who engage in those practices in Africa.

Recent studies have shown that women mainly adopt risky sexual behaviours to cope with negative income shocks (e.g., agricultural and climatic shocks, illness or death of family members) and suggest that economic shocks are a substantial piece of the HIV puzzle in Africa (3). Researchers have previously explored the link between poverty and HIV through cash transfers targeting adolescent girls and young women (4). Cash transfers can be an effective way to reduce risky sexual practices among women by increasing their income. However, evidence regarding their effectiveness to prevent HIV is mixed. In Malawi, cash transfers were found to reduce HIV prevalence by 64% (5) while they had limited effect in Kenya (6) and no effect in South Africa (7). The POWER project is based on the assumption that income variability matters more than income level for HIV acquisition, which could explain why cash transfer programs are not always effective. While the literature shows mixed evidence regarding the effect of poverty on HIV prevalence among women (8), there is robust evidence demonstrating that women adopt risky sexual behaviours as a way to cope with economic shocks. Economic shocks are defined as any events that lead to a sudden increase in expenditure level (e.g., illness, COVID-19) or a decrease in income level. In Kenya, it was estimated that an illness of a family member led sex workers to increase unprotected sex by 21 per cent (9). Another study found that each time a village in Africa suffered from a drought, it led to an increase in HIV prevalence in that village by 11 per cent (10). Hence, there is a need to explore whether the effectiveness of some cash transfer programs in reducing HIV acquisition is due to their role in protecting against economic shocks. If so, cash transfers may fail to prevent HIV infection if the amount offered is not large enough to protect against economic shocks or if they are received outside a shock period.

There are several reasons why economic shocks may lead to STIs and HIV acquisition. The main reason lies in the fact that the low access to formal well-paid jobs and productive assets makes women in Africa prone to use commercial and transactional sex as risk-coping strategies. Such strategies may be attractive given that they allow women to raise money quickly and they can earn up to three times more compared to other occupations (11). Relationships developed with men through commercial and transactional sex can also result in other financial or in-kind gifts, which could also help women to cope with economic shocks (12). Women who engage in commercial and transactional sex are incentivised to provide unprotected sex because men are generally willing to pay more for unprotected sex. It is estimated that sex workers can earn 9% more in Kenya and 66% more in India (11) for an unprotected sex act. In addition to unprotected sex, commercial and transactional sex are risky practices because they often lead to multiple, concurrent sexual partners and partners at greater risk of HIV infection (older men and sex workers’ clients). In addition to increasing risky behaviours, economic shocks may also lead to STI and HIV infection if households cut health expenditures concerning STI treatment and HIV prevention services.

Determining whether an economic shock is the missing piece of the HIV puzzle is critical as economic shocks are very common in Africa and these women often lack risk-coping strategies. Economic shocks are a key issue for many African households. At least 60% of African households report large and sudden losses in income every year (13) but only 10% of the poorest households in Africa benefit from social safety net programs to negate income fluctuations (14). Among the shocks experienced by households, health shocks are of particular concern given that they are more frequent, more costly and more concentrated among the poor than other economic shocks (15). If women adopt risky sexual behaviours to cope with negative economic shocks providing women with formal risk-coping strategies could be a very promising approach to preventing HIV. However, no previous study has been designed to specifically answer this research question. Hence, there remain important gaps in understanding.

**Objectives {7}**

**Research Hypothesis**

The main research hypothesis is that women who engage in transactional and commercial sex in Africa use risky sex as a way to cope with economic shocks. Providing health insurance for themselves and their economic dependents is effective to reduce risky sexual behaviours and in turn, prevent STIs and HIV infection among women and girls who engage in commercial and transactional sex.

**Research Objectives**

The general objective of this research is to understand and explore the role of economic shocks (e.g., illness, COVID-19) as a driver of the heightened vulnerability of women to HIV. The identification of the role of economic shocks in STI and HIV acquisition will inform the design of novel public health interventions to tackle STIs and HIV. The RCT aims to estimate the effectiveness of health insurance for high-risk women and their economic dependents as a strategy to prevent STIs and HIV. The POWER project addresses important gaps in the current literature by answering the following research questions:

- What is the role of economic shocks on STIs and HIV?
- How do economic shocks affect STIs and HIV?
- What is the effect of health insurance as a risk-coping strategy to reduce risky sexual behaviours, STIs and HIV?

**Trial design {8}**

This study is a stratified cluster randomised controlled superiority trial with two groups: control and treatment. Private participatory randomisation will be conducted with a target of a 1:1 allocation stratified by the type of risky activity, commercial sex or transactional sex.

**Methods: Participants, interventions and outcomes**

**Study setting {9}**

The trial is being conducted in Yaoundé, Cameroon. Cameroon was chosen because it has one of the highest gender disparity in HIV globally, adolescent girls aged 15–19 are five times more likely to be infected with HIV than their male counterparts [2% versus 0.4%] (16).

The implementing partner is John Hopkins Cameroon Program (JHCP), an NGO experienced in conducting research for John Hopkins University. Recruitment of participants using respondent-driven sampling (RDS) is hosted by two partner Community-based organisations (CBOs), Horizon Femmes (HF) and RENATA. HF and RENATA were chosen because of their extensive experience and excellent ongoing work in STI and HIV prevention with the target population. Both organisations were recommended by the implementing partner JHCP.

All women and girls engaging in commercial and transactional sex who have received a recruitment coupon and meet the eligibility criteria can participate. Recruitment of participants into the study began in June 2021. The randomisation of participants began in October 2021.

After negotiations with several health insurance and care providers in Cameroon and on the advice of local partners (JHCP, HF and RENATA), GMC assurances and Cité Verte Hospital were the chosen health insurance and healthcare providers. GMC assurances was chosen as they offered the best value insurance package. The choice of hospital was based on the quality of care, availability of medication on site and the positive culture and experience in providing care to stigmatised and vulnerable groups.

**Eligibility criteria {10}**

Eligibility inclusion criteria for participation are:

1. Be engaged in commercial or transactional sex in Yaoundé, Cameroon
2. In receipt of a recruitment coupon (given by RDS)
3. Aged over the age of 15
4. Not married
5. Identify as woman
6. Have at least one economic dependent living in Yaoundé
7. Have their own, password protected, phone and be able to respond to SMS messages
8. Be HIV negative at enrolment

Eligibility exclusion criteria for participation are:

1. Those under 21 years old who cannot obtain parenteral or guardian consent to participate in the study

**Who will take informed consent? {26a}**

Trained enumerators take informed consent by reading and explaining the information sheets (containing information on the study, objectives of the research, risks and benefits and explaining that participation is voluntary) to potential participants. Participants then have the opportunity to have an informed discussion with the enumerators regarding the study. Participants 21 years old and over are considered majors, and those under the age of 21 are minors. Majors can offer their own consent to participate in the study. For minors, an adult representative/guardian must undergo a similar informed consent, receiving the same information as the participant, before the assent from the minor in a separate room. Participants are never referred to as girls or women who engage in commercial or transactional sex to the adult representative, to minimise adverse consequences. If an adult representative does not offer their consent, a minor cannot participate. If consent is offered, a trained enumerator will then take a minor through an informed assent process, following the same steps as that for a major. Adult representatives offering consent and all participants (minor and major) will receive a hard copy of the consent. An electronic copy, for researchers, of all consent forms (adult representative, minor, major) is stored electronically using an ODK-based survey.

**Additional consent provisions for collection and use of participant data and biological specimens {26b}**

Participants are informed during the informed consenting process that they will undergo biological tests at three different points in the study. HIV and STIs will be tested at these three points, and they will also receive testing for other bacteria and fungi in the vagina to understand their non-sexually transmitted infection status during the final testing for the study. It is explained to participants that the reason we collect their data and biological specimens is to meet the research objective. Consent for biological specimens required for infection testing, using vaginal swabs and blood tests, is obtained during the consenting process.

**Patient and public involvement**

The partner CBOs who maintain a close relationship with the target population were consulted throughout the design phase of the intervention and the study. Formative interviews and focus groups with girls and women engaging in commercial and transactional sex were also conducted to ensure the appropriateness of language employed in the survey, willingness to participate in the study and willingness to accept the intervention. A key concern was that participants would be unwilling to accept the results of the randomisation process. We sought feedback during formative interviews and focus groups on the randomisation design by presenting potential participants with alternative randomisation designs e.g., computer randomisation, public randomisation, and private randomisation. The chosen design was a private participatory draw as this was described as the fairest and most transparent design. We asked participants to support the recruitment of participants in the study using RDS, a chain referral sampling method. Participants were asked to invite up to three of their peers to participate in the study. Once the analysis of the data is complete, participants and the general public will be invited to a dissemination workshop where the results will be presented.

**Interventions**

**The explanation for the choice of comparators {6b}**

The trial will compare participants who receive a free health insurance product with participants who receive nothing. It is expected that there will be equal proportions for the control and treatment groups and that the two groups (treatment and control) will be similar as they are taken from the same population (girls and women engaging in commercial and transactional sex).

**Intervention description {11a}**

The insurance product, provided by GMC Insurance, covers the participant and up to six of their (nominated) economic dependents and is offered for 12 months. An economic dependent is anyone that depends on the participant financially for health care and can include family members, friends and sex work intermediaries (e.g., pimp). The health insurance product provides free health coverage, there are no out-of-pocket costs, with a cumulative ceiling for each group (the participant and their economic dependents) of 500 000 FCFA (= 638,55 GBP, in 2022 prices) following an illness or an accident and is only valid at the Cité Verte Hospital, Yaoundé, Cameroon. The negotiated ceiling of 500 000 FCFA provides good coverage of health expenses for 12 months in Cameroon. If the sum of 500,000 FCFA has been reached (by the participant and their economic dependents combined), participants must pay for the health expenses themselves. Before the group reaches the 500 000 FCFA limit, the participant will be notified to ensure that they are informed when consuming health care. Costs covered by the insurance product are: consultation fees; pharmaceutical fees; medical fees and; hospitalization fees for non-chronic pathologies. Costs not covered by the insurance product are: chronic diseases (e.g. diabetes, high blood pressure, asthma, sickle cell anaemia, obesity...); maternal health care (prenatal care, delivery, postnatal care); illness resulting from civil or foreign war, civil commotion or riot; injury following a violent quarrel except in cases of self-defence; epidemics declared as such by a competent authority (e.g. COVID-19); cosmetic care; dietary care; weight loss or rejuvenation; sterility or abortion treatment; psychiatric treatment; alcoholism; dialysis; dental care and; ophthalmologic care.

The insurance product was rolled out on the 15^th^ of November 2021 and ended on the 14^th^ of November 2022.

Those in receipt of free health care (treatment group participants and their economic dependents) will receive a health insurance pamphlet (explaining the health insurance product and the terms and conditions of usage) and a health insurance card (see online supplemental file appendix 3). Insured parties seeking free healthcare at Cité Verte Hospital must arrive with their health insurance card and photo identification (ID). The research team will generate IDs which are only valid for the POWER project, for those who do not hold identification.

At the Cité Verte Hospital, those covered by the POWER insurance product are received at a dedicated reception, staffed by an enumerator (who is involved in the administration of the ODK-based survey) and an administrative staff member of the Cité Verte Hospital. Staff at the POWER reception identifies the consumer of free healthcare using the insurance card and photo ID. Eligible patients are then triaged and taken through the Cité Verte Hospital patient care process.

**Criteria for discontinuing or modifying allocated interventions {11b}**

All adverse events will be treated on a case-by-case basis. In the event of an unanticipated adverse event that may cause direct harm or threat to the participants or others that are related to study implementation, the local JHCP staff will notify the PI. An unanticipated event report will be submitted promptly to the Cameroon National Ethics Committee and the UCL ethics committee per protocol.

**Strategies to improve adherence to interventions {11c}**

The main strategies employed to encourage participant adherence to the intervention is to ensure that participants and their economic dependents have the best possible experience whilst accessing care at the hospital and to reduce the barriers to access to care. A key concern is that participants or their nominated economic dependents will not go to the hospital to seek care when sick because they face large barriers to access e.g., lack of understanding of illness, distrust in healthcare or not having enough money to get to the hospital. Treatment participants receive information at randomisation, midline and endline regarding what is provided as part of the health insurance product, how to access it and whom to contact if they are having difficulties. Data is collected during the midline and endline survey regarding the participant's experience of using the health insurance product. If the participant has not used the insurance product because they forgot they are part of the treatment group the participant is reminded of the health insurance product and encouraged to use the intervention if needed by the enumerator and the site supervisor at the end of the survey. If the participant has had a bad experience whilst accessing care at the hospital, this is reported to the site supervisor and to the research staff. This problem will be discussed whilst the participant is being surveyed by the enumerator. At the end of the survey, the site supervisor will discuss their experience with the participant and ensure that any issues will be resolved and encourage them to use their health insurance product if needed. Experienced POWER enumerators are based at the hospital throughout the intervention period to support the patients throughout their clinical pathway. If there are any issues with treatment or access to care, the POWER staff report it directly to the research team for a resolution. The CBOs and specifically peer leaders and mobilisers support the adherence of participants to the intervention by keeping in regular contact with the participants throughout the lifetime of the project. Regular contact allows participants with concerns regarding the project or the intervention specifically the opportunity to speak up. Any problems are communicated to the research team and resolved. All resolutions are discussed with the participants. Peer leaders and mobilisers use their strong relationships with the participants and their status in this vulnerable population to engage participants and encourage adherence to the intervention.

**Relevant concomitant care permitted or prohibited during the trial {11d}**

There are no restrictions on concomitant care.

**Provisions for post-trial care {30}**

Any participants who suffer harm, e.g., psychological distress, as a consequence of the POWER project will be supported by the relevant CBO's psychological support services. Those in the control group, that remain in the study until the end of the project, will receive the same health insurance product for themselves and their economic dependents for 12 months.

**Outcomes {12}**

**Overview**

There are several methods of data collection. The socio-economic and biobehavioural surveys occur over three time periods: baseline, 6 months after baseline (midline), and 12 months after baseline (endline). The SMS survey occurs fortnightly, commencing just after the completion of the baseline for 52 weeks. The surveys are administered in both French and English, depending on the participant's language preference.

**Primary outcome measure**

Measured using questionnaires developed by the study team where not otherwise stated at 1, 6 and 12 months:

1. STI acquisition measured using % test positive for syphilis, % test positive for chlamydia, % test positive for gonorrhoea. STI symptoms prevalence measured using % who had any symptoms of STI in the last 30 days: vaginal discharge, lower abdominal pain outside of diarrhoea or menstrual period, wound or ulcer on genitals, burns during urination, swelling in the groin, itching in the genital area, pain during the sexual act, bleeding outside of period

2. HIV status measured using % testing HIV positive

**Secondary outcome measures**

Secondary outcomes include:

1. Physical health of participants measured using % of participants with good health, % of participants with a chronic illness other than Hepatitis B, the average number of chronic illnesses, % of participants who were sick in the last 30 days, % who sought treatment from a qualified health worker if sick in the last 30 days, % of participants with abnormal levels of Gardnerella vaginalis (GV), Mobiluncus, Candida and streptococcal in the vagina (only at endline)

2. Physical health of economic dependents measured using % of economic dependents with good health, % of economic dependents with a chronic illness, the average number of chronic illnesses per economic dependent, % economic dependents who were sick in the last 30 days, % economic dependent who were sick during sex act with last client, % economic dependents who were sick during sex act with the penultimate client, % of children with good health

3. Beliefs regarding control over own health measured using % who believe that nothing can be done to prevent illness, % who believe that being in good health is a matter of luck

4. Poverty measured using total expenditures, asset index, income from sex work, amount of savings, % who have Informal shock-coping strategies, average amount of transfers received and sent, % who have a debt, amount of the debt, food insecurity

5. Education/training measured using % school dropout, new enrolment in schools, % who are undertaking professional training

6. Other occupation measured using transitions out of sex work, % who have other occupation, share of sex work income in total income

7. Risky sexual behaviours measured using % of condom use with last and penultimate client using a double list experiment and the colourbox method, a newly indirect elicitation method developed by the team members, number of clients per week, type of sex acts performed with last client, self-reported risk preferences with sex (out of 10 scale), % of were pregnant the last 6 months, % who performed both transactional sex and sex work

8. Violence measured using % who experienced violence (threats, physical, sexual, emotional/psychological) the last time they had sex with a client, % who experienced violence the penultimate time they had sex with a client, % who experienced violence by a current partner in the last 6 months, % who experienced violence by a sugar daddy in the last 6 months, % who experienced violence by another sex worker in the last 6 months, % who experienced violence by an occasional client in the last 6 months, % who experienced violence by a regular client in the last 6 months, % who experienced violence by police in the last 6 months, % who experienced violence by their pimp in the last 6 months, % who experienced violence by hostel owners in the last 12 months

9. Stress measured using perceived stress scale 4 (PSS-4)

10. Self-efficacy measured by participants' ability to deal with issues in different domains of their life

11. Loneliness measured using UCLA 3 Item Loneliness scale

12. Discrimination measured using Everyday Discrimination Scale (Short Version)

13. Stigma measured using % who have at least one of their family or friends aware that the participant has having paid sex, % who expect discrimination by family if HIV positive, % who consider themselves as a sex worker

14. HIV and STI knowledge measured using knowledge of participants regarding ways HIV can be transmitted, average knowledge of STI symptoms

15. Social networks measured using the number of friends in sex work, the number of peers who provide financial support

16. Mental health measured using % that are depressed using PHQ-9 scale, % who are happy, % satisfied with life, % with good self-esteem

**Indicators of implementation of the intervention**

Finally, we will measure the good implementation of the intervention by looking at:

1. Health care utilisation
2. Out-of-pocket expenditures
3. Health shocks
4. Patient satisfaction

**Participant timeline {13}**

A schematic diagram is presented in Figure 1.

Figure 1: Schedule of enrolment, interventions, and assessments

**Sample size {14}**

The baseline study has recruited 1,508 unmarried girls and young women engaging in transactional (753) and commercial (755) sex in Yaoundé, Cameroon. Currently, 1,147 participants have been randomised via participatory randomisation, 568 participants allocated to the control arm and 579 allocated to the treatment arm. To maximise statistical power, STI-positive participants receive free treatment at baseline to start the trial with an STI-free cohort. Participants will also receive free treatment at midline to allow for the possibility to observe more than one STI per participant over the trial period. Free treatment will also be provided at endline for ethical reasons. Assuming that 20% of participants will get at least one STI over the trial period, this sample size is sufficient to show a decrease of 30% (6 percentage points) in the incidence of any STI in the treatment group assuming 80% power and a significance level of 5%. To conduct analysis separately among the sub-samples of those engaging in commercial and transactional sex, the MDE is a ~40% reduction (8.3 percentage points) in STI incidence. Assuming an HIV incidence of 4% over the trial period, the sample size would allow the detection of a decrease of 65% in HIV incidence (2.6 percentage points).

**Recruitment {15}**

Participants are recruited using RDS, a chain-referral recruitment method that allows for unbiased population estimates. Initial participants (seeds) were identified by the CBOs. Seeds, and all subsequent participants, were required to recruit up to three of their friends that met the eligibility criteria. A motivation of 500 FCFA (=0.64 GBP, in 2022 prices) was given to participants for each (up to three) participant recruited. Study staff, alongside peer leaders/mobilisers, will follow participant recruitment, calling participants who have not recruited anyone onto the study to encourage them to do so.

**Assignment of interventions: allocation**

**Sequence generation {16a}**

Randomisation is conducted at the individual level using a participatory approach with a target of a 1:1 ratio to treatment or control group stratified by engagement in commercial or transactional sex. Formative research, conducted before randomisation, found that participatory randomisation was the preferred method of randomisation by the target population. Participatory randomisation was deemed fairer and more transparent than computer-based randomisation.

**Concealment mechanism {16b}**

A protocol has been developed to minimise the opportunity to fix randomisation so participants all receive the treatment. Two balls, one orange (allocating participants to the treatment group) and one white (allocating participants to the control group) are placed in a long black opaque bag. Participants are required to draw one ball out of the bag and cannot see inside. Hence, participant allocation to the treatment and control arm is known by the participants, the enumerators and supervisors at the time of the draw.

**Implementation {16c}**

Before the draw is conducted, the enumerators will take the participant through the informed consent process, explain the randomisation process and define the meaning of the two different coloured balls. After the participant consents to take part in the study and before the randomisation process begins participants are asked several questions to ensure that they understand how randomisation will be conducted.

All participants who give consent for participation and who fulfil the inclusion criteria will be randomised. Randomisation is conducted with a participant in a private room by two enumerators under the supervision of a site supervisor. No draw can occur in the absence of those three witnesses. This is to ensure that the assignment of participants to the treatment or control group is conducted fairly and transparently. One enumerator leads the randomisation whilst the other completes the participant's responses in an ODK-based survey. Firstly, the enumerator will ask the participant to feel the bag to demonstrate the bag is empty and that the draw is not rigged by the POWER team. Then the enumerator will place one orange and one white ball in the empty bag in front of the participant. The participant will then place then hand and arm, up to their elbow, in the bag. A drawstring is tightened underneath the participant's elbow so they are unable to see inside the bag. The enumerator holding the drawstring is standing up by the participant to ensure that the participant cannot see inside the bag. The participant must then shuffle the balls around in their hand inside the bag. When the participant is ready to draw, they must notify the enumerator to loosen the drawstring. Participants only have one chance to draw a ball from the bag. When the draw is complete, and participants have been allocated to their treatment group, treatment participants are requested to re-consent to the study after receiving a description of the insurance product. If treatment participants chose to remain in the study, they will receive the health insurance product. If not, they will withdraw their participation from the study.

**Assignment of interventions: Blinding**

**Who will be blinded {17a}**

Participants and care providers will not be blinded. Researchers will not be blinded during the conduct of the study to support the smooth-running implementation of the intervention. However, data analysts will be blinded during the data analysis period.

**Data collection and management**

**Plans for assessment and collection of outcomes {18a}**

There are several methods of data collection. The socio-economic and biobehavioural surveys occur over three time periods: baseline, 6 months after baseline (midline), and 12 months after baseline (endline). The surveys are administered in both French and English, depending on the participant's language preference. The primary outcomes of the study are STI acquisition and HIV status will be measured via the biobehavioural and socioeconomic survey conducted at baseline, midline and endline. At baseline, we will test for syphilis, chlamydia, gonorrhoea and trichomoniasis. For financial reasons, we will only collect HIV and the two most prevalent STIs at the midline. At endline we will test for all the STIs tested at baseline (syphilis, chlamydia, gonorrhoea and trichomoniasis). In addition, to allow for a better understanding of the participants' non-sexually transmitted infections status, the levels of GV, Mobiluncus, Candida and streptococcal in the vagina will also be tested. Testing the levels of these bacteria and fungi is a secondary outcome. It is important as sexual activity is associated with an increased risk of imbalance of these bacteria or fungi in the vagina which may lead to infections such as Bacterial vaginosis (BV), Candidiasis and Group B Strep infection. Evidence suggests that there is a positive relationship between a person having these infections and an STI and HIV(17). Biological tests (blood tests and vaginal swabs) to test for STIs, HIV and non-sexually transmitted bacteria and fungi are conducted at the same time as the socioeconomic survey.

Other secondary outcomes are measured via the biobehavioural and socioeconomic survey conducted at baseline, midline and endline. Additional measures for secondary outcomes will also be captured in the fortnightly SMS survey. The survey questionnaire will include sociodemographic questions, income, health status, demand for healthcare, economic shocks, STI prevention, risky sexual behaviours, violence, personality traits and preferences.

The procedures for each test are as follows. For HIV, serological testing will be based on a venous blood draw and follows national procedures, including both pre-and post-test counselling. HIV testing utilised dual rapid testing (Alere Determine ^TM^ HIV – ½ and OraQuick HIV – ½ ) based on the national testing protocol (Figure 2). At midline and endline, participants with undetermined tests will be referred to the military hospital of Yaoundé for an ELISA test. Patients who test positive will be linked into care.

Figure 2: HIV testing algorithm based on Cameroon national protocol

Syphilis testing will utilize rapid VDRL and Treponema pallidum hemagglutination assays (TPHA) for the detection of syphilis antibodies and classification of primary, active and past infection (Figure 3). The testing algorithm will be based on the national protocol, but FTA-ABS is not available in Cameroon for confirmatory testing. Subsequently, we will not exclude false-positive VDRL tests which will be considered positive. For midline and endline we will only consider active cases of syphilis. Following the blood draw, participants received post-test counselling until results were available 30–40 minutes after testing. Participants who test positive will receive treatment for free. We will contact them to find out if the treatment was taken and, for those who have a partner, to understand if they provided information about their infection to their partner.

Figure 3: Syphilis testing algorithm adapted from the Cameroon national protocol

Chlamydia testing will utilize Chlamydia Rapid Test Kit on vaginal swabs and include pre-and post-test counselling. A swab specimen collected from the patient will be treated with an extraction reagent. The Chlamydia rapid test dipstick is immersed in the extraction sample. The liquid migrates through the absorbent area and along the membrane on the Chlamydia rapid test. As the mixture flows along the membrane of the Chlamydia rapid test, the complex is captured by the antibody immobilized in the test zone (T) of the membrane, producing a visible rose-pink colour band and at the same time in the control zone (C). A rose-pink line in the control zone (C) indicates the Chlamydia rapid test is working properly. When only a control line appears with no test line, the Chlamydia trachomatis antigen has not been detected and the Chlamydia rapid test result is considered negative.

Gonorrhoea testing will utilize Gonorrhoea Rapid Test Kit on vaginal swabs and include pre-and post-test counselling. A swab specimen collected from the patient will be treated with an extraction reagent. The Gonorrhoea rapid test dipstick is immersed in the extraction sample. The liquid migrates through the absorbent area and along the membrane on the Gonorrhoea rapid test. As the mixture flows along the membrane of the Gonorrhoea rapid test, the complex is captured by the antibody immobilized in the test zone (T) of the membrane, producing a visible rose-pink colour band and at the same time in the control zone (C). A rose-pink line in the control zone (C) indicates the Gonorrhoea rapid test is working properly. When only a control line appears with no test line, the Gonorrhoea trachomatis antigen has not been detected and the Gonorrhoea rapid test result is considered negative.

There is no rapid test for trichomoniasis, the testing for these STIs will be subcontracted to CRESAR (Research centre for the armed forces). CRESAR is a laboratory which has been based on the JHCP premises for over 20 years. Serum samples will be stored in a freezer (-80°C). Cervicovaginal samples are discarded immediately after testing. The collected data are stored in a register and in a computer. In electronic format, the data is stored in Excel format (without a password). The persons having access to the data are the staff and the trainees authorised by the head of CRESAR. The information stored in the register and computer is, for each participant, the identification number, age, date of collection, time of collection (only for cervicovaginal samples), date and time of receipt, name of the collection site, name of the person transporting the samples from the collection site to CRESAR, name of the person receiving the samples at CRESAR, results of the tests performed. The samples are kept for the duration of the study, at the end of the study the samples will be disposed of by incineration.

**Plans to promote participant retention and complete follow-up {18b}**

**Participant retention**

Participants are not incentivised or coerced to participate in the study. During the informed consent process, the benefits of the study are outlined in detail to encourage participant retention. The primary benefit highlighted is the possibility that this project will contribute to the proposal of public policy intervention to improve the quality of life of young women who engage in transactional and commercial sex.

In addition, all participants receive compensation for their time and travel costs. Participants are compensated 2000 FCFA (2.57 GBP, in 2022 prices) for their time and travel when responding to on-site questionnaires. This compensation will act to reduce any barriers to participant participation (e.g., transport fees). In addition, they receive free STI and HIV testing on-site at baseline, midline and endline. Those with a positive STI test will be offered free treatment (regardless of group status). This will be covered by POWER directly and not by the health insurance package for those in the treatment group. HIV-positive participants will be referred to initiate antiretroviral treatment.

Participants receive airtime to reply to weekly SMS diary surveys and an additional financial incentive. To achieve higher retention and completion rates a compensation for their time of 1000 FCA francs (1.28 GBP, in 2022 prices) is offered to all participants with a completion rate of diaries superior to 90% over the last 30 days.

To retain participants in the control group, the project offers the same health insurance product to all eligible control group participants at the end of the data collection period. To be eligible for this health insurance product, control participants must remain in the study by completing all follow-up surveys and responding to the SMS survey.

Further to the potential benefits of the study, study site staff are also responsible for supporting the development and implementation of procedures to support the retention of participants, using their knowledge of the context. Peer leaders (at HF) and mobilisers (at RENATA), CBO staff members who work closely with the target group, will use their extensive experience and networks to encourage participants to continue participation in the study. The study Advisory Group will also be consulted about the retention strategies of participants.

**Participant withdrawal**

Participation is voluntary and participants can withdraw at any time, without giving reasons, by notifying a project staff member. Participant information already collected will be suppressed from the dataset. For control participants, withdrawal from the study means that participants are no longer entitled to the health insurance product provided at the end of the study. Withdrawal of participants on the treatment arm of the study will not impact any services or medical treatment they receive or any relationships they have with those treating them outside of the POWER project. However, any treatment or services that the participant and/or their nominated economic dependents receive will no longer be covered by health insurance provided by the POWER project. If the participant chooses to withdraw, they must choose to either stop ongoing treatments for themselves and their economic dependents or find an alternative means of paying for them.

**Data management {19}**

Socioeconomic questionnaires will be administered by enumerators in the field. All potential participants will be screened before recruitment onto the study to ensure they have not already participated. Participant responses are entered directly into an ODK-based questionnaire using Android tablets and uploaded to the ODK secure server in the UK when a 3G/WIFI connection is available (immediately, or at the end of each day). On survey completion, the data will be transferred to the UCL Secure Server, a fully encrypted (SSL) server with a direct data link to the android device. The ODK-based questionnaire is programmed with a logic that minimises missing data (e.g., enumerators will not be able to skip questions) and data quality (e.g., range checks will be conducted regularly). Any issues regarding data quality will be reported to the Principal investigator (PI) and directly to the enumerators and the team in the field.

Biobehavioural survey data will be held on the laboratory registry, which is a confidential registry that does not contain any identifiable personal information. The laboratory registry is paper-based (there are no facilities to have an electronic database) and will be kept securely in a locked cabinet. Paper-based data (laboratory records) will be securely transferred to the JHCP office where they will be transferred to a centralized, 128-bit encrypted and password-protected database. All electronic and encrypted data will be transferred securely to the UCL Secure Server.

Responses, using participant telephones, to the weekly SMS sexual diaries are sent directly to the dedicated server at MTN Cameroon. This server is dedicated to this study and will only contain POWER project data. This server is fully encrypted (SSL) and complies with all international standards and norms in terms of hardware and software security (firewall, antivirus, hubs, VPN). The server is in a data centre in Yaoundé, Cameroon, which is a secure building with fire protection systems, generators, redundant air conditioning, and access security). Only the PI of the study and the director of DINA surveys will have access to the data held on the password-protected server. Data is downloaded and exported to excel weekly and stored immediately on the UCL Secure Server. The data will be deleted from the MTN Cameroon dedicated server for up to three years after data collection, or earlier if requested.

In-depth interviews and focus groups will be audio-recorded using an encrypted capture device. The recordings are anonymised and transcribed verbatim in the interviewees' language (French) and transcribed into English using Microsoft Word by an independent professional transcription company. All files will be password protected at all times. They will be transferred to the UCL Secure Serve before deleting them from the device. Subsequently, they will be imported into Nvivo for anonymisation and analysis. Only the research team will have access to the in-depth interview and focus group data.

All data will be stored in accordance with University College London's data storage policies.

After completion of the project, data will be made openly available through the UCL data repository, subject to the ability to remove personally identifiable information. Data will be deposited with UCL research data repository (<https://www.ucl.ac.uk/library/research-support/research-data-management/ucl-research-data-repository>), where it will be given a Digital Object Identifier (DOI). The DOI will be cited in project reports and journal publications through a Data Access Statement or citation list. Metadata will also be made available to 3rd party research catalogues, such as https://datamed.org/.

Anonymised data will be made openly available using a permissive licence, such as Creative Commons Attribution (CC-BY). If complete anonymity cannot be provided, it will be made available through a controlled access system. If data must be made available via controlled access, interested parties will be asked to provide information on their research (purpose, institutional affiliation, ethics approval) and sign a data sharing agreement indicating they will comply with the consent form and will not attempt to re-identify individuals. Access requests will be evaluated by the project team in the first instance. In cases where an access request is denied and the requester wishes to appeal, the request will be escalated to the UCL Research Governance Committee for consideration.

**Confidentiality {27}**

The following measures have been put in place to ensure data confidentiality and to respect the privacy of participants:

1. Implementation of and compliance with the code of ethics

All study staff, inclusive of hired and trained experienced local enumerators and interviewers, must sign a commitment to comply with the code of ethics. Research partners with access to data (JHCP and DINA surveys) have signed a sub-contract with a clause which ensures that all data collected must remain confidential.

1. Participation is confidential

Socioeconomic and biobehavioural data collection and in-depth interviews and focus groups will be conducted in a private location at the CBOs. Participants must not share a telephone to be enrolled in the SMS survey. They are required to confirm that they have their own, password-protected, telephone and can respond to SMS messages. This is to ensure that only the participant reads and responds to the SMS survey.

1. Secure storage of data

During the trial, all paper-based records will be kept securely in a locked cabinet, in a locked office, and securely transferred to the JHCP office where they will be transferred to a centralized, 128-bit encrypted and password-protected database. All electronic data will be stored on the UCL Secure Server.

1. Aggregation and anonymisation of data

Individual responses will be deidentified and aggregated for analysis and reporting. All published findings, including any quotes used, will not allow for individuals to be identified. All results will be presented in aggregate form.

**Plans for collection, laboratory evaluation and storage of biological specimens for genetic or molecular analysis in this trial/future use {33}**

Once participants have completed the ODK-based socioeconomic survey (0 months, 6 months and 12 months) they will also undergo etiological testing for STIs (chlamydia, gonorrhoea, trichomonas, syphilis) and HIV. Participants will also undergo testing for additional bateria and fungi in the vagina (GV, Mobiluncus, Candida and streptococcal) at endline. Rapid tests are conducted in the CBOs laboratory for chlamydia, gonorrhoea, syphilis and HIV. No rapid test for Trichomoniasis is available in Cameroon, therefore testing will be subcontracted to CRESAR (Centre de Recherche sur la Santé des Armées), a laboratory which has been based in the JHCP premises for over 20 years.

Serum samples will be stored in a freezer (-80°C). Cervicovaginal samples are discarded immediately after testing. The collected data are stored in a register and a computer. In electronic format, the data is stored in Excel format (without a password). The persons having access to the data are the staff and the trainees authorised by the head of CRESAR. The information stored in the register and computer is, for each participant, the identification number, age, date of collection, time of collection (only for cervicovaginal samples), date and time of receipt, name of the collection site, name of the person transporting the samples from the collection site to CRESAR, name of the person receiving the samples at CRESAR, results of the tests performed. The samples are kept for the duration of the study, at the end of the study the samples will be disposed of by incineration.

**Statistical methods**

**Statistical methods for primary and secondary outcomes {20a}**

All statistical analyses will be conducted using STATA. Descriptive statistics will first be used to summarise participant characteristics between the study groups. To investigate the effect of health insurance on primary and secondary outcomes, the analysis will use an intention-to-treat (ITT) approach where participants will be analysed in their allocated randomised study group. If there are non-compliers, i.e., participants and economic dependents who do not use the health insurance product when sick, we will also estimate the local average treatment effect (LATE). To investigate the effect of health insurance on primary and secondary outcomes, baseline characteristics will be compared for the treatment and control groups and determine whether there is differential attrition between the two groups. Outcomes for the treatment versus the control group will then be compared accounting for any baseline or attrition differences using ordinary least squared. The analysis will be conducted for all women and then separately for women engaging in transactional and commercial sex separately. A causal mediation analysis will be conducted to assess how much of the effect of health insurance on STI and HIV acquisition operates through risky sexual behaviours.

**Interim analyses {21b}**

The interim analysis will be conducted at midline and we will seek guidance from the ethics committee if the intervention is harmful to participants, i.e., if we observe a deterioration of primary outcomes.

**Methods for additional analyses (e.g., subgroup analyses) {20b}**

Further analysis will investigate if the effect of economic shocks on STIs and HIV varies depending on (1) women’s characteristics, (2) the person affected by the shocks (3) the type of shocks (e.g., illness, job loss), (4) the intensity of the shock and (5) available coping strategies (e.g., savings).

To explore the robustness of the cost-effectiveness deterministic and probabilistic sensitivity analysis will be conducted to explore the impact of uncertainty of parameter estimates, assumptions and model structure on the results.

**Methods in analysis to handle protocol non-adherence and any statistical methods to handle missing data {20c}**

Non-compliance issues will be investigated by estimating the local average treatment effect (LATE).

In addition, we will enquire about cases of STIs that were untreated at baseline and will also provide evidence of the impact of the intervention by removing participants who test positive at midline and endline because they did not take their treatment at baseline.

**Plans to give access to the full protocol, participant-level data and statistical code {31c}**

Data and statistical code will be made openly available through the UCL data repository, subject to the ability to remove personally identifiable information. Data will be deposited with UCL research data repository (<https://www.ucl.ac.uk/library/research-support/research-data-management/ucl-research-data-repository>), where it will be given a Digital Object Identifier (DOI). The DOI will be cited in project reports and journal publications through a Data Access Statement or citation list. Metadata will also be made available to 3rd party research catalogues, such as <https://datamed.org/>. Qualitative responses will not be uploaded to this repository due to the potential to identify participants.

Anonymised data will be made openly available using a permissive licence, such as Creative Commons Attribution (CC-BY). If complete anonymity cannot be provided, it will be made available through a controlled access system. If data must be made available via controlled access, interested parties will be asked to provide information on their research (purpose, institutional affiliation, ethics approval) and sign a data sharing agreement indicating they will comply with the consent form and will not attempt to re-identify individuals. Access requests will be evaluated by the project team in the first instance. In cases where an access request is denied and the requester wishes to appeal, the request will be escalated to the UCL Research Governance Committee for consideration.

**Oversight and monitoring**

**Composition of the coordinating centre and trial steering committee {5d}**

There is no formal committee for the coordination and oversight of this trial. The PI will oversee and coordinate all aspects of the trial with the support of the project’s researchers. All decisions impacting the project protocol will be discussed with the lead researchers. The Advisory Group made up of experts internal (project staff from UCL and JHCP, partners from local CBOs RENATA and Femmes Horizons) and external (senior staff from UCL and JHCP) to the project, will be consulted at key decision points.

**Data monitoring {21a}**

There is no formal committee for monitoring data collected as part of the POWER project. Data monitoring will be undertaken by a researcher supervised by the PI. Questionnaire responses will be checked daily to determine any problems with the data collection instruments.

**Adverse event reporting and harms {22}**

The potential risks of participation in this trial are minimal. An adverse event will be defined as a participant becoming significantly distressed as a consequence of their involvement in the trial. All adverse events, as a consequence of participation in the POWER study, occurring after the entry into the study and until the end of the study will be recorded and will be treated on a case-by-case basis. In the event of an unanticipated adverse event that may cause direct harm or threat to the participants or others that are related to study implementation, the local JHU staff will notify the PI. Per protocol, an unanticipated event report will be submitted promptly to the Cameroon National Ethics Committee and the UCL ethics committee.

The study will not be providing any direct treatment. Participants who tested positive for HIV and/or STIs (Syphilis, Chlamydia, Gonorrhoea, Trichomoniasis) will be referred to POWER-supported health facilities with the capacity for follow-up treatment. They will also be referred to RENATA and Horizons Femmes (POWER CBOs) for pastoral support and advice.

We are not expecting any major adverse events from the implementation of these studies (socioeconomic survey, weekly SMS diaries, interviews, focus groups, laboratory testing activities). However, we will establish a procedure so that participants who require any medical attention as a consequence of laboratory testing activities, can have full access. Psychological stress may occur during the survey, interviews and focus group, as well as following the disclosure of HIV and STI results. Counsellors will be trained and made available to respond to stress resulting as part of the study.

During the study, we might have some minor adverse events such as syncope during blood drawing for laboratory testing. Patients will be informed of this risk. Laboratory technicians will be trained on how to deal with these minor events. An unlikely adverse event could be an infection at the site of the needle prick, the chance of which will be minimized through observing international standards of blood sample collection. The respondent will be properly counselled on how, where and when to seek treatment in case of such an incident.

Mild levels of stress may occur during interviews, as the questions cover sensitive topics and are likely to be unfamiliar to the respondents. Interviewers will be extensively trained to respond to stress resulting from any of these possible scenarios.

**Frequency and plans for auditing trial conduct {23}**

The PI meets weekly with researchers conducting the daily activities of the trial online to discuss and review trial progress, and at least monthly with the full investigator team. Any adverse events or issues in trial progress will be promptly communicated with the investigator team at these meetings or between meetings if necessary. Progress to the trial funder will be reported annually. Any trial protocol amendments will be reported to the University College London Research Ethics Committee and National Ethics Committee in Cameroon. The Steering Committee, comprised of key representatives from JHCP, RENATA, HF, Dina Surveys, Cité Verte Hospital, and GMC assurances, will meet once per year for input into trial progress and will be contacted between meetings as needed. Auditing of the intervention will take place consistently throughout the year via a qualitative study and quantitative data collection collected at midline. Staff from JHCP will be based at the hospital to help POWER participants navigate the healthcare system.

**Plans for communicating important protocol amendments to relevant parties (e.g., trial participants, ethical committees) {25}**

Any amendments to the protocol will be provided to the University College London Research Ethics Committee and National Ethics Committee in Cameroon and will also be communicated to all researchers on the project and project partners (JHCP, RENATA, HF, Dina surveys).

**Dissemination plans {31a}**

The project partners (JHCP, RENATA, HF, Dina surveys), the Ministry of Health in Cameroon, and the funders will be provided with a summary of the findings of the trial. The findings will also be reported in high-impact scientific journal articles and presented at scientific national and international conferences and in public forums as requested.

A national workshop at the end of the project will disseminate findings to national stakeholders (70+). Participants will include governmental organisations (Ministry of Health, CNLS), financial and technical partners (WB, USAID, ILO, GIZ, CHAI, AFD, UNICEF, WHO, CDC, UNFPA), researchers working on HIV/AIDS (University Yaoundé I, John Hopkins Cameroon Program), non-governmental organisations working with young women at high risk of HIV in Cameroon (e.g. Horizons Femmes, RENATA, CAMNAFAW, Alternatives Cameroun, Colibri, KidAIDS, SWAA littoral, Synergie des Sciences Sociales et Humaines, UFAPROD-M), and grassroots organisations of female sex workers. A policy brief will also be disseminated by those stakeholders through internal newsletters.

Throughout the lifetime of the project, accessible briefing notes will be created for the Institute for Global Health (UCL), the PI’s UCL webpage and social media (Twitter - <https://twitter.com/power_ucl>. A press release of research findings will be created upon publication which will be communicated with journalists in the general media (e.g., CNN). Digital stories will also be produced to engage the public on the effect of economic shocks on the sexual behaviours of young women in Cameroon. These digital stories will be presented in Cameroon and at the ESRC festival of social science in order to engage the public in this research.

**Discussion**

POWER aims to advance understanding of the structural drivers of STIs and HIV by analysing the role of a recently identified and under-researched factor: economic shocks. This study is the first to provide evidence of the effectiveness and value for money of a formal shock-coping strategy, health insurance, to prevent STIs and HIV in Africa using an RCT. Hence, POWER will generate robust and rigorous information on the causal mechanisms that determine STI and HIV acquisition in vulnerable women in Cameroon. This information will be a vital contribution to support the pressing need to achieve universal health coverage and to identify novel cost-effective interventions to address the burden of HIV in Africa. By contributing to both debates, the research project will enhance the efficiency of UK and international aid and has the strong potential to have a long-lasting impact on shaping the research agenda on HIV.

A study of this size and complexity does have several limitations and risks. Primarily, there may be participant loss since we already observe an attrition of 23% between recruitment and randomisation. In addition, issues in the implementation of the intervention are likely to be frequent given the lack of experience in implementing health insurance projects of this size in Cameroon. Additionally, issues inherent to the quality of care could lead to an under-utilisation of healthcare services, which would prevent the evaluation of the effect of the intervention of the primary and secondary outcomes.

**Trial status**

Protocol version: Final version as of 10 March 2023

Recruitment dates: The date of first enrolment was 2 June 2021 and participants are currently being followed-up.

Enrolment status: No longer recruiting.

**Abbreviations**

- POWER - Protecting women from economic shocks to fight HIV in Africa
- JHCP – John Hopkins Cameroon Programme
- UCL – University College London
- DSH – Data Safe Haven
- CBO – Community-based organisation
- HF – Horizon Femme
- STI – Sexually transmitted infection
- HIV – Human immunodeficiency virus
- CEO – Chief Executive Officer
- PI – Principal investigator
- UKRI – UK Research and Innovation Council
- FLF – Future Leaders Fellowship

**Declarations**

**Acknowledgements**

We would like to say thank POWER participants.

**Authors' contributions {31b}**

AL conceived of and led the design of the study, led the proposal and the protocol development, contributed to the drafting of the manuscript and gave the final approval of the manuscript. SS led the drafting of the manuscript and contributed to the design of the study. FP contributed to the design of the study drafting of the manuscript and gave final approval of the manuscript. ED, SB, IMN and UT contributed to the design of the study and gave final approval of the manuscript. All authors read and approved the final manuscript.

**Funding {4}**

This research is funded by a UKRI Future Leaders Fellowship (round 2, 2020-2024) which is awarded by the MRC/ESRC (grant number: MR/S031790/1) awarded to Dr Aurélia Lépine. UKRI has no role in the design or conduct of the study or the interpretation and dissemination of the findings. UKRI may be contacted at [communications@ukri.org](mailto:communications@ukri.org) or <https://www.ukri.org/about-us/contact-us/>.

**Availability of data and material {29}**

The team at UCL will have access to the final dataset, held on the DSH at UCL. POWER partners can apply for access to the, anonymised, final data set via the DSH.

**Ethics approval and consent to participate {24}**

Manuscripts reporting studies involving human participants, human data, or human tissue must:

1. Include a statement on ethics approval and consent (even where the need for approval was waived): ATTACHED The work covered in this manuscript has been conducted with the ethical approval of all relevant participants (University College London Research Ethics Committee and National Ethics Committee in Cameroon, see online supplemental file appendix 2 and 3)
2. Include the name of the ethics committee that approved the study and the committee’s reference number, if appropriate a. University College London Research Ethics Committee (ref: 17341/00) and b. National Ethics Committee in Cameroon (CNERSH, ref: 2020/12/1313)

**Consent for publication {32}**

Not applicable.

**Competing interests {28}**

The authors declare that they have no competing interests.

**References**

1. UNAIDS. HIV prevention among adolescent girls and young women. 2016.

2. Mullick S, Watson-Jones D, Beksinska M, Mabey D. Sexually transmitted infections in pregnancy: prevalence, impact on pregnancy outcomes, and approach to treatment in developing countries. Sex Transm Infect. 2005;81(4):294–302.

3. Cust H, Jones H, Powell-Jackson T, Lépine A, Radice R. Economic shocks and risky sexual behaviours in low- and middle-income countries: a systematic review of the literature. Journal of Development Effectiveness. 2021;13(2):166–203.

4. Pettifor A, MacPhail C, Nguyen N, Rosenberg M. Can money prevent the spread of HIV? A review of cash payments for HIV prevention. AIDS Behav. 2012;16(7):1729–38.

5. Baird SJ, Garfein RS, McIntosh CT, Özler B. Effect of a cash transfer programme for schooling on prevalence of HIV and herpes simplex type 2 in Malawi: a cluster randomised trial. The Lancet. 2021;379(9823):1320–9.

6. Handa S, Halpern CT, Pettifor A, Thirumurthy H. The Government of Kenya’s Cash Transfer Program Reduces the Risk of Sexual Debut among Young People Age 15-25. PLoS One. 2014;9(1):e85473.

7. Pettifor A, MacPhail C, Hughes JP, Selin A, Wang J, Gómez-Olivé FX, et al. The effect of a conditional cash transfer on HIV incidence in young women in rural South Africa (HPTN 068): a phase 3, randomised controlled trial. The Lancet Global Health. 2016;4(12):e978–88.

8. Shelton JD, Cassell MM, Adetunji J. Is poverty or wealth at the root of HIV? The Lancet. 2005;366(9491):1057–8.

9. Robinson J, Yeh E. Transactional Sex as a Response to Risk in Western Kenya. American Economic Journal: Applied Economics. 2011;3(1):35–64.

10. Burke M, Gong E, Jones K. Income Shocks and HIV in Africa. The Economic Journal. 2015;125(585):1157–89.

11. Cunningham S, Shah M, editors. The Oxford Handbook of the Economics of Prostitution [Internet]. Oxford University Press; 2016 [cited 2023 Mar 10]. Available from: https://doi.org/10.1093/oxfordhb/9780199915248.001.0001

11. Robinson J, Yeh E. Risk-Coping through Sexual Networks Evidence from Client Transfers in Kenya. J Hum Resour. 2012 Jan 1;47(1):107–45.

13. Christiaensen L. Agriculture in Africa – Telling myths from facts: A synthesis. Food Policy. 2017;67:1–11.

14. World Bank. The State of Social Safety Nets 2015 [Internet]. Washington, DC: World Bank; 2015 [cited 2023 Mar 7]. Available from: https://openknowledge.worldbank.org/handle/10986/22101

15. Wagstaff A, Lindelow M. Are health shocks different? Evidence from a multishock survey in Laos. Health Econ. 2014;23(6):706–18.

16. ICF International, Ministère de la Santé Publique. Cameroun Enquête Démographique et de Santé et à Indicateurs Multiples (EDS-MICS) 2011. Calverton, Maryland, USA; 2012.

17. Lewis FMT, Bernstein KT, Aral SO. Vaginal Microbiome and Its Relationship to Behavior, Sexual Health, and Sexually Transmitted Diseases. Obstet Gynecol. 2017 Apr;129(4):643–54.

**Figure legend**

# Figure 1: Schedule of enrolment, interventions, and assessments

# Figure 2: HIV testing algorithm based on Cameroon national protocol

Figure 3: Syphilis testing algorithm adapted from the Cameroon national protocol
