# Supplementary material for: The effect of protecting women against economic shocks to fight HIV in Cameroon, Africa: The POWER randomised controlled trial
Source: PLoS Med. 2024 Oct 24;21(10):e1004355. doi: 10.1371/journal.pmed.1004355 (PMC11500901; doi:10.1371/journal.pmed.1004355)
Supplement: Attachment — Submitted filename: response to reviewers.pdf [file pmed.1004355.s008.pdf]

Reviewer #1: *The paper entitled "Protecting Women from economic shocks to prevent HIV in Africa: Evidence from the POWER randomized controlled trial in Cameroon" deals with a very important topic in Sub-Saharan Africa. The topic is not novel; however, this could still be of interest since this is a new study population (women in Cameroon) and adds to the limited literature on this topic in this population. Furthermore, and most importantly, it is a study that presents the results of an intervention. However, the article needs some improvements:*

- The introduction should better explain how the economic situation influences other psychosocial variables related to sexual behaviour. It is essential to explain the path that poverty takes before it leads to unprotective behaviour. Does poverty directly influence condom use, for example? Or does it influence the socio-cognitive variables that activate preventive behaviour? In the specific case of women, what role does poverty play in self-efficacy (an essential variable for negotiating safe sex with partners)? Read the following articles on HIV prevention in women (but not only, do an active search for articles of this nature):
1) Gender, HIV knowledge and prevention attitudes among adolescents living with HIV participating in an economic empowerment intervention in Uganda." AIDS care vol. 33,7 (2021): 888-896. doi:10.1080/09540121.2020.1844860
2) Socio-demographic, marital and psychosocial predictors of safe sex behaviour among Mozambican women at risk for HIV/AIDSA. African Journal of AIDS Research, 17(4), 323-331. https://eur01.safelinks.protection.outlook.com/?url=https%3A%2F%2Fdoi.org%2F10.2989%2F16085906.2018.1536672&data=05%7C02%7Ca.lepine%40ucl.ac.uk%7Cb16dd0b10a8441c7f42c08dc3867034c%7C1faf88fea9984c5b93c9210a11d9a5c2%7C0%7C0%7C638447262611036031%7CUnknown%7CTWFpbGZsb3d8eyJWIjoiMC4wLjAwMDAiLCJQIjoiV2luMzIiLCJBTiI6Ik1haWwiLCJXVCI6Mn0%3D%7C0%7C%7C%7C&sdata=G9mf150BY%2F0QtyBJZ3c%2FBmAmrXOuuU%2FoFB7UbWTblsI%3D&reserved=0.
3) Socio-demographic, marital, and psychosocial factors associated with condom use negotiation self-efficacy among Mozambican women at risk for HIV infection. International Journal of Behavioral Medicine, 24(6), 846-855. https://eur01.safelinks.protection.outlook.com/?url=https%3A%2F%2Fdoi.org%2F10.1007%2Fs12529-017-9681-0&data=05%7C02%7Ca.lepine%40ucl.ac.uk%7Cb16dd0b10a8441c7f42c08dc3867034c%7C1faf88fea9984c5b93c9210a11d9a5c2%7C0%7C0%7C638447262611040789%7CUnknown%7CTWFpbGZsb3d8eyJWIjoiMC4wLjAwMDAiLCJQIjoiV2luMzIiLCJBTiI6Ik1haWwiLCJXVCI6Mn0%3D%7C0%7C%7C%7C&sdata=%2FXMpzKkUbAhEYGe7eflNuO6uNPlBvRuT8%2F2QbJl%2FAtM%3D&reserved=0.

Thank you very much for your insightful comments. We fully agree that indeed poverty can affect sexual behaviours through several channels. We have now provided more information on those channels by reviewing the literature in the behavioural economics and psychosocial literature, both your suggested and through an additional literature search. We have made the following changes to the introduction:

[Introduction, paragraph 2] “In addition to income variability, poverty is linked to certain behaviours that can lead to making risky or poor decisions through cognitive bias [1]. It is hypothesised that scarcity and poverty can lead individuals to prioritise meeting immediate goals over longer-term goals, potentially influencing their risk and time preferences. In this context, it is possible that individuals experiencing poverty may take sexual risks in order to meet their immediate consumption needs [1].”

- The methodology and results are presented in a very clear and well-articulated manner.

Thank you for your comment.

- In the discussion (and in the introduction) it is important to present the results of the effectiveness of other non-medical interventions (more social and psychosocial) in African women, particularly in sub-Saharan Africa. Are social and psychosocial interventions successful? If so, to what extent? What are its strengths and weaknesses? Here too, in the debate, more culturally diverse studies need to be presented. The following articles can help improve these two points:
1) Effectiveness of educational intervention among seropositive women about knowledge about HIV sexual transmission." Revista brasileira de enfermagem vol. 76,3 e20220371. 21 Aug. 2023, doi:10.1590/0034-7167-2022-0371
2) The Effect of Theory-Based HIV/AIDS Educational Program on Preventive Behaviors Among Female Adolescents in Tehran: A Randomized Controlled Trial." Journal of reproduction & infertility vol. 21,3 (2020): 194-206.
3) Testing the effectiveness of two psychosocial interventions - ACCENT and Didactic - to prevent HIV/AIDS behavioral risk factors in Mozambican women: a randomized controlled study. AIDS Care. https://eur01.safelinks.protection.outlook.com/?url=https%3A%2F%2Fdoi.org%2F10.1080%2F13548506.2023.2229236&data=05%7C02%7Ca.lepine%40ucl.ac.uk%7Cb16dd0b10a8441c7f42c08dc3867034c%7C1faf88fea9984c5b93c9210a11d9a5c2%7C0%7C0%7C638447262611045876%7CUnknown%7CTWFpbGZsb3d8eyJWIjoiMC4wLjAwMDAiLCJQIjoiV2luMzIiLCJBTiI6Ik1haWwiLCJXVCI6Mn0%3D%7C0%7C%7C%7C&sdata=m6cL8UlOVxZJrs0wVJywGoWgXBH0iQte5uiU7W6fLOE%3D&reserved=0.
4) Testing the effectiveness of two psychosocial interventions on socio-cognitive risk factors for HIV/AIDS in Mozambican Women: A randomized controlled trial. AIDS Education and Prevention, 33(3), 169-186. https://eur01.safelinks.protection.outlook.com/?url=https%3A%2F%2Fdoi.org%2F10.1521%2Faeap.2021.33.3.169&data=05%7C02%7Ca.lepine%40ucl.ac.uk%7Cb16dd0b10a8441c7f42c08dc3867034c%7C1faf88fea9984c5b93c9210a11d9a5c2%7C0%7C0%7C638447262611050640%7CUnknown%7CTWFpbGZsb3d8eyJWIjoiMC4wLjAwMDAiLCJQIjoiV2luMzIiLCJBTiI6Ik1haWwiLCJXVCI6Mn0%3D%7C0%7C%7C%7C&sdata=NBgfwEHInjx3mTd5CtcZ4vS2gZJHyXKxvCmSPsSYZ44%3D&reserved=0
5) Evaluating the effectiveness of incentives to improve HIV prevention outcomes for young females in Eswatini: Sitakhela Likusasa impact evaluation protocol and baseline results. BMC

Public Health 20, 1591 (2020). B*https://eur01.safelinks.protection.outlook.com/?url=https%3A%2F%2Fdoi.org%2F10.1186%2Fs12889-020-09680-*[2]*8&data=05%7C02%7Ca.lepine%40ucl.ac.uk%7Cb16dd0b10a8441c7f42c08dc3867034c%7C1faf88fea9984c5b93c9210a11d9a5c2%7C0%7C0%7C638447262611055369%7CUnknown%7CTWFpbGZsb3d8eyJWIjoiMC4wLjAwMDAiLCJQIjoiV2luMzIiLCJBTiI6Ik1haWwiLCJXVCI6Mn0%3D%7C0%7C%7C%7C&sdata=X%2FTcn6KrtHCTydETtwSeeAb4ltdow2PCclQMyCRbneQ%3D&reserved=0*

Thank you for this comment that will improve our introduction to set the scene with other interventions that are used. We also add some less detailed discussion . The introduction now reads:

[Introduction, paragraph 3] “Structural interventions to address the underlying causes of HIV risk through improving income level include cash transfers. These measures can increase income and discourage risky commercial transactions, especially when merit-based incentives such as school attendance or remaining HIV negative are included. However, the evidence for their effectiveness is mixed. Only three of eight studies found cash transfers were effective to protect against HIV and STIs and only when conditions were set [3]. One possible explanation for this is that cash transfers may have limited effectiveness in protecting households against economic shocks and poverty if the value is too low or if they are received outside the period of shocks [4]. Conversely, interventions that aim to address the psychosocial factors contributing to risky sexual behaviours have shown some success. These interventions include lottery-based financial incentives to remain STI-free [6]. Such financial incentive design has been proven to be effective since it targets individuals who have a high preference for risk and hence who are more likely to engage in risky sexual behaviours [6]. In addition, interventions to improve women’s self-efficacy in negotiating condoms has also been found to be effective [7]. Self-efficacy is an important aspect of HIV risk that can be increased through education, open conversations and self-affirmation [8,9]. A literature review conducted recently found that structural interventions, such as social empowerment and economic strengthening, were significantly effective in reducing self-stigma that impedes HIV testing, HIV prevention and HIV treatment [2]. Finally, there is some evidence that cognitive behavioural therapy was effective to improve preventive health behaviour of female sex workers in India through an increase in self-image [10]"

And the discussion:

“We contribute to the growing evidence that women engaging in transactional sex should be considered as a key population in the fight against HIV. We show how a structural intervention is more protective against HIV for women in transactional sex than FSWs, yet currently they receive far fewer HIV prevention services and are ineligible to PrEP in many places [2]. Our study highlights the effectiveness of targeting studies at specific causes of economic vulnerability (i.e. economic shocks) where cash transfers have mixed impacts on HIV and risky behaviours. It is clear structural interventions alongside psychosocial interventions targeting stigma, self-efficacy and other causes of risky behaviours that could improve testing,

identification and treatment of HIV have the potential to tackle the ongoing HIV epidemic in sub-Saharan Africa"

- After revising the manuscript, please make sure the abstract accurately summarizes it.

We have now made sure this was the case. This is the new abstract:

[Abstract] "Background: Women in sub-Saharan Africa are disproportionately affected by the HIV epidemic. Young women are twice as likely to be living with HIV as men of the same age and account for 64% of new HIV infections among young people. Many studies suggest that financial needs, alongside biological susceptibility, are a leading cause of the gender disparity in HIV acquisition. New robust evidence suggests women adopt risky sexual behaviours to cope with economic shocks, the sudden falls in household's income or consumption power, enhancing our understanding of the link between poverty and HIV. We test if health insurance protects against economic shocks, reducing the need for vulnerable women to engage in risky sexual behaviours and reducing HIV incidence.

Method & Findings: We conducted a randomised controlled trial to test the effectiveness of a formal shock coping strategy to prevent HIV among high-risk women (registration number: ISRCTN 22516548). We recruited 1,506 adolescent girls and women involved in transactional sex or commercial sex in Yaounde, Cameroon, using snowball sampling. Half of the study participants were randomly assigned to receive free health insurance for themselves and their economic dependents for 12 months. The control group received the intervention at the end of the study. We collected data on socioeconomics, health and sexual behaviour and our primary outcomes of sexually transmitted infections (STIs) and HIV biomarkers at baseline, 6 months after randomisation (midline) and 12 months after randomisation (endline). We found that study participants who engaged in transactional sex and were assigned to the treatment group were less likely to become infected with HIV (odds ratio (OR)=0.109 (95% confidence interval (CI)[0.014,0.870]); p=0.036). There was no evidence of a reduction in HIV incidence among female sex workers. There was also no effect on STI acquisition. The main limitations of this study were the challenges of collecting reliable biomarkers for STIs and the lower incidence of HIV in the FSW group, which prevented detection of study effects.

Conclusion: The study provides the first evidence of the effectiveness of a formal shock strategy for HIV prevention among women who engage in transactional sex in Africa. The effectiveness of the intervention is mediated by a reduction in out-of-pocket payments rather than an increase in health care utilisation, which showed only small increases. The study suggests that policymakers in Africa should consider implementing formal shock strategies for HIV prevention among women who engage in transactional sex, taking into account the high-risk nature of this activity.

- Thank you for the opportunity to read and review this article.

We thank you very much for your comment on the importance to understand how poverty may affect sexual behaviours through psychosocial characteristics. This was a useful addition to the manuscript.

***Reviewer #2**: Statistical review*

This paper reports a randomised trial investigating whether provision of health insurance reduces HIV incidence in individuals engaging in transactional and commercial sex. The trial provides interesting results. I had some comments on the statistical methods and reporting:

Thank you for your well thoughtful and detailed comments and we appreciate the opportunity to increase the clarity and accuracy of our statistical analysis. We have made adjustments in line with your comments and added details where relevant below under each comment.

1. Abstract: briefly mention what happened with the control group, especially that it involved receiving the intervention 12 months later.

We have now added this.

2. Abstract: I would explicitly mention the primary outcome of the study.

In the abstract we have now explicitly mentioned the primary outcomes and in the paper details of when the control received the intervention.

3. Abstract (and results): for the primary analysis results, please add 95% CI for the odds ratio and put a more precise p-value following PLOS medicine guidance. I wasn't clear why the results for the transactional sex population were provided rather than th' pooled results.

All 95% CI's have been added, we report both the pooled and separate strata results because the difference of effects between transactional and commercial sex strata is an important element of the paper. Changes have been made through to improve the clarity of those results.

4. Abstract "We showed that the intervention allowed women to leave transactional sex." – perhaps add the proportions in each group that left?

Note that although we agree it was important, we have now removed this sentence from the abstract since PLOS guidelines mention that we cannot report secondary outcomes in the abstract unless we report them all. Given the high numbers of secondary outcomes we have decided to only present the effect on primary outcomes in the abstract. We however present this information in the results section (see Table 4 presented below).

5. Abstract: I would report secondary outcomes like proportion using condoms in the same way as the primary outcome (and add n in each group for this as it's not a randomised comparison given the difference in those leaving transactional sex.

We have removed this from the abstract for the above reason but are presenting these results as you suggest in the result section with an updated table 4:

Table 4: Effect of health insurance on sexual behaviours behaviour outcomes

	Baseline						Midline/Endline						Difference			
	Treatment			Control			Treatment			Control						
Sexual Behaviour & Secondary Outcomes	obs	n	% / mean	obs	n	% / mean	obs	n	% / mean	obs	n	% / mean	ME	95% CI	ME	95% CI
**<u>Commercial Sex</u>**																
Number of regular clients in the last 7 days	287	-	3.84	289	-	3.89	180	-	2.96	185	-	2.99	-0.034	-0.585 , 0.518	-0.036	-0.587 , 0.516
Number of occasional clients in the last 7 days	281	-	0.05	282	-	4.54	180	-	3.25	184	-	2.82	0.429	-0.306 , 1.165	0.382	-0.348 , 1.113
Number of clients in the last 7 days (combined)	279	-	8.85	281	-	8.46	180	-	6.21	184	-	5.74	0.129	-0.721 , 0.979	0.112	-0.711 , 0.936
Number of sex acts in the last 7 days	287	-	11.75	290	-	11.12	246	-	5.60	243	-	5.84	-0.242	-1.352 , 0.868	-0.364	-1.447 , 0.718
Number of vaginal sex acts over previous two clients‡	578	562	97.2%	580	567		360	356	98.9%	370	365	97.8%	0.002	-0.014 , 0.018	0.002	-0.014 , 0.018
Number of oral sex acts over previous two clients‡	574	69	12.0%	580	93	16.0%	359	34	9.5%	370	52	16.0%	-0.046*	-0.093 , 0.001	-0.036	-0.082 , 0.01
Number of anal sex acts over previous two clients‡	577	12	2.1%	580	15	2.6%	360	5	1.4%	370	0	2.6%	0.014**	0.002 , 0.026	0.013**	0.001 , 0.025
Self-reported riskiness of clients (average /10)‡	578	-	0.38	580	-	0.38	360	-	0.59	380	-	0.55	0.039**	0.009 , 0.070	0.038**	0.007 , 0.068
Used condom at last sex act - Direct questioning†	289	245	84.8%	290	249	85.9%	180	161	89.4%	184	162	88.0%	0.014	-0.051 , 0.079	0.013	-0.051 , 0.077
Quit commercial sex work†	-	-	-	-	-	-	218	49	22.5%	221	48	21.7%	0.008	-0.070 , 0.085	0.008	-0.067 , 0.084
Suffered violence from sexual partner	289	83	28.7%	290	124	42.8%	246	79	32.1%	243	79	32.5%	-0.004	-0.087 , 0.079	0.003	-0.081 , 0.087
Stress (PPS-4)	-	-	-	-	-	-	246	-	7.98	243	-	7.98	0.004	-0.227 , 0.236	-	- -
Loneliness (UCLA 3 point scale)	289	-	4.97	290	-	5.11	246	-	5.67	243	-	5.60	0.066	-0.251 , 0.383	0.064	-0.253 , 0.691
**List Experiment**																
Used condom at last sex act - List experiment	-	-	-	-	-	-	-	-	-	-	-	-	-0.111	-0.348 , 0.125	-0.109	-0.345 , 0.128
**<u>Transactional Sex</u>**																
Number of regular papy in the last 7 days	290	-	1.49	278	-	1.43	186	-	1.42	213	-	1.37	0.059	-0.190 , 0.307	0.080	-0.169 , 0.330
Number of occasional papy in the last 7 days	290	-	0.46	277	-	0.51	186	-	0.73	213	-	0.75	-0.021	-0.191 , 0.150	0.003	-0.170 , 0.176
Number of papy in the last 7 days	290	-	1.95	278	-	1.94	263	-	1.52	267	-	1.69	-0.164	-0.439 , 0.110	-0.140	-0.414 , 0.133
Number of sex acts in the last 7 days	290	-	2.64	278	-	2.74	263	-	2.13	267	-	2.48	-0.358*	-0.781 , 0.065	-0.320	-0.742 , 0.102
Number of vaginal sex acts‡	520	515	99.0%	593	476	80.3%	367	363	98.9%	412	405	98.3%	0.006	-0.011 , 0.024	0.007	-0.011 , 0.024
Number of oral sex acts‡	520	68	13.1%	493	87	17.6%	366	47	12.8%	414	64	15.5%	-0.023	-0.074 , 0.027	-0.019	-0.068 , 0.030
Number of anal sex acts‡	518	13	2.5%	494	11	2.2%	367	3	0.8%	414	3	0.7%	0.001	-0.012 , 0.014	-0.001	-0.014 , 0.012
Self-reported riskiness of clients (average /10)‡	520	-	0.47	495	-	0.48	358	-	0.35	400	-	0.34	0.014	-0.012 , 0.039	0.008	-0.018 , 0.034
Used condom at last sex act - Direct questioning†	290	144	49.7%	278	140	50.4%	186	82	44.1%	213	89	41.8%	0.023	-0.074 , 0.120	0.016	-0.082 , 0.114
Quit transactional sex work†	-	-	-	-	-	-	247	63	25.5%	249	42	16.9%	0.086**	0.015 , 0.158	0.084**	0.013 , 0.155
Suffered violence from sexual partner	290	35	12.1%	278	36	12.9%	263	32	12.2%	267	23	8.6%	0.036	-0.017 , 0.088	0.036	-0.016 , 0.089
Stress (PPS-4)	-	-	-	-	-	-	263	-	8.05	267	-	8.04	0.012	-0.284 , 0.308	-	- -
Loneliness (UCLA 3 point scale)	290	-	5.82	287	-	5.60	263	-	5.35	267	-	5.25	0.099	-0.179 , 0.038	0.101	-0.181 , 0.384
**List Experiment**																
Used condom at last sex act - List experiment	-	-	-	-	-	-	-	-	-	-	-	-	0.152*	-0.021 , 0.324	0.150*	-0.025 , 0.325
**<u>Pooled (commercial + transactional)</u>**																
**Continuous outcomes - OLS**																
Number of regular clients/papy in the last 7 days	577	-	2.66	567	-	2.68	366	-	2.18	398	-	2.12	0.135	-0.158 , 0.428	0.092	-0.191 , 0.376
Number of occasional clients/papy in the last 7 days	571	-	2.7	559	-	2.55	366	-	2.33	397	-	2.04	0.233	-0.077 , 0.544	0.167	-0.132 , 0.466
Number of papy's or clients/papy in the last 7 days	579	-	5.32	568	-	5.18	509	-	3.24	510	-	3.24	0.030	-0.398 , 0.457	-0.010	-0.418 , 0.397
Number of sex acts in the last 7 days	579	-	7.53	5.68	-	7.02	509	-	3.81	510	-	4.08	-0.279	-0.891 , 0.333	-0.391	-0.969 , 0.187
Number of vaginal sex acts‡	1098	1077	98.1%	1073	1043	97.2%	727	719	98.9%	782	770	98.5%	0.004	-0.007 , 0.016	0.005	-0.007 , 0.017
Number of oral sex acts‡	1094	137	12.5%	1073	180	16.8%	725	81	11.2%	784	116	14.8%	-0.035**	-0.069 , -0.001	-0.024	-0.058 , 0.009
Number of anal sex acts‡	1095	25	2.3%	1074	26	2.4%	727	8	1.1%	784	3	0.4%	0.007	-0.001 , 0.016	0.007	-0.002 , 0.016
Self-reported riskiness of clients/papy (average /10)‡	1098	-	0.42	1075	-	0.43	718	-	0.47	770	-	0.44	0.031***	0.008 , 0.054	0.027**	0.005 , 0.049
Used condom at last sex act - Direct questioning†	579	389	67.2%	568	389	68.5%	366	243	66.4%	397	251	63.2%	0.014	-0.051 , 0.079	0.036	-0.029 , 0.101
Quit either commercial or transactional†	-	-		-	-	-	465	112	24.1%	470	90	19.1%	0.008	-0.070 , 0.085	0.043	-0.008 , 0.095
Suffered violence from sexual partner	579	118	20.4%	568	160	28.2%	509	111	21.8%	510	102	20.0%	0.018	-0.032 , 0.068	0.028	-0.021 , 0.078
Stress (PPS-4)	-	-	-	-	-	-	509	-	8.02	510	-	8.01	0.008	-0.182 , 0.197	-	- -
Loneliness (UCLA 3 point scale)	579	-	5.4	568	-	5.35	509	-	5.51	510	-	5.42	0.085	-0.125 , 0.296	0.085	-0.127 , 0.296
**List Experiment**																
Used condom at last sex act - List experiment	-	-	-	-	-	-	-	-	-	-	-	-	0.034	-0.113 , 0.180	0.041	-0.106 , 0.187

Significance levels: * p<0.1, ** p<0.05, *** p<0.01

Notes: Dependent binary variables (†) were estimated using logistic regression reporting marginal effects. All other continuous outcome variables were estimated using OLS reporting marginal effects. (log) indicate where the outcome is logged in the OLS model. Unadjusted = compares outcomes of follow-up data without adjustment. Adjusted = adjusts for baseline levels of the outcome, if the outcome was collected during midline, then baseline controls, namely log of adult equivalent expenditure, number of economic dependents, household size and if the respondent has a child. The list experiment was estimated using OLS using the double list experiment method (Lépine et al., 2020) hence twice the observations as each respondent appears twice.

‡ = Data taken from the last two sex acts per sex worker.

We have also added some minor changes to the interpretation of these results:

[results paragraph 1] "Table 4 shows the impact of the intervention on risky sexual behaviours and other secondary outcomes. The intervention led to a significant increase in the likelihood of quitting transactional sex by 8.6 percentage points (16.9% vs 25.5%), or a 50% increase (ME = 0.086 (95% CI [0.015,0.158]); p=0.018) in likelihood of leaving sex work. There is an increase in condom use at the last sex act of around 15 percentage points, or a 43% increase, when measured using the list experiment method (ME=0.152 (95% CI[-0.021,0.324]); p=0.085) and we observe a decrease in the number of sex acts by 0.36 points on average for women in transactional sex (16% drop), which is statistically significant at 10% (ME=-0.358 (95% CI[-0.781,0.065]); p=0.097). For FSWs, health insurance had mixed results on risky sexual behaviours. The intervention was not effective in allowing FSWs to leave sex work and it did not increase condom use. However, it led to very small increases in the likelihood of engaging in anal sex (ME=0.014 (95% CI [0.002,0.026]); p=0.023) and on the riskiness of clients (ME=0.039 (95% CI [0.009,0.070]); p=0.012). The study found that the intervention had no impact on the likelihood of experiencing physical violence, as well as the degree of loneliness

and stress levels among participants. Appendix **Error! Reference source not found.** in **Error! Reference source not found.** provide both unadjusted and adjusted odd ratios."

6. Abstract "We showed that the intervention effectiveness operates through the reduction in health shocks since the increase in healthcare use following the intervention was low." - this sentence comes a bit out of the blue as there was no mention of use of healthcare or 'health shocks' in the results.

We have now removed this sentence and provided clarity on our definitions of shocks.

7. Abstract "We estimated that in our trial, the cost for each HIV infection averted is £4,667 among the cohort of women engaging in transactional sex." - this might be better in the results section, and ideally would have some uncertainty interval provided. (Similar comment applies to this in the discussion of the paper, although it is clearer there that it is an informal calculation - perhaps removing this statement from the abstract but keeping it in the discussion would be appropriate).

We have now moved this to the discussion which now reads:

“We perform a back-of-the-envelope calculation for the cost-effectiveness. The total cost of healthcare claimed by the intervention was £42,003, which averted 9 HIV infections in the transactional sex strata. This gives a cost-effectiveness ratio per HIV infection averted of £4,667 for women engaged in transactional sex. This means that the intervention is likely to be highly cost-effective when scaled up, and this figure does not take into account the positive externalities associated with preventing HIV in the wider community.”

8. Table 1: given the groups were randomly allocated there's no need to test for imbalance between them as significant results must be type I errors. Thus, I'd remove the p-values (note that only 1 p-value out of >20 was significant, consistent with this being by random chance).

We have updated table 1 and removed p-values as we agree that statistically significant differences must be type I errors. We have now mentioned in the text that only 1 difference was statistically significant consistent with this being random. We have now added paragraph below table 1:

"**Error! Reference source not found.** demonstrates successful randomisation. Any imbalances are type-I errors and within the expectations of randomisation.”

Table 1 and the interpretation now reads:

	Commercial Sex						Transactional Sex					
	Treatment			Control			Treatment			Control		
	Obs	N	Mean / %	Obs	N	Mean / %	Obs	N	Mean / %	Obs	N	Mean / %
Age, years	286	-	31.2	287	-	31.0	290	-	25.1	278	-	24.3
Experience - months in sex work	289	-	56.6	290	-	54.9	289	-	38.8	278	-	36.0
**Nationality**												
Cameroonian (%)	289	288	100%	290	288	99%	290	290	100%	278	277	100%
**Education**												
Currently studying	289	41	14%	290	56	19%	290	129	44%	278	136	49%
**Highest achieved level of education**												
Primary	288	43	15%	290	41	14%	290	24	8%	278	19	7%
Secondary primary cycle	288	104	36%	290	86	30%	290	70	24%	278	70	25%
Secondary second cycle	288	82	28%	290	91	31%	290	112	39%	278	91	33%
Superior	288	55	19%	290	63	22%	290	80	28%	278	93	33%
No education	288	4	2%	290	1	3%	290	3	0%	278	2	1%
Other	288	0	0%	290	8	0%	290	1	1%	278	3	1%
**Marital status**												
Never married	289	258	89%	290	257	89%	290	282	97%	278	272	98%
Separated	289	20	7%	290	19	7%	290	5	2%	278	5	2%
Divorced	289	3	1%	290	7	2%	290	0	0%	278	0	0%
Widowed	289	8	3%	290	7	2%	290	3	1%	278	1	0%
**Economic Variables**												
Household size	289	-	4.3	290	-	4.4	290	-	5.7	278	-	5.6
Number of economic dependents	289	-	3.1	290	-	3.1	290	-	2.9	278	-	3.0
Number of child economic dependents	289	-	1.8	290	-	1.9	290	-	1.6	278	-	1.9
Have at least one child economic dependents	289	235	81%	290	254	88%	290	239	82%	278	241	87%
Number of biological children	289	-	2.1	290	-	1.9	290	-	1.2	278	-	1.2
Household expenditure per adult equivalent	289	-	54,804 CFA	290	-	47,426 CFA	290	-	32,933 CFA	278	-	38,776 CFA
**Sex work and behaviours**												
Number of clients/sugar daddies in typical week	268	-	8.5	265	-	8.2	290	-	2.8	278	-	2.6
Number of sex acts in a typical week	286	-	17.9	288	-	17.4	290	-	4.0	278	-	3.8
Average earnings from sex work	289	-	92,332 CFA	290	-	93,748 CFA	290	-	45,048 CFA	278	-	42,986 CFA
Used PrEP before	289	25	9%	290	19	7%	290	0	0%	278	1	0%
Had an HIV test in the last 12 months	288	267	93%	290	268	92%	290	167	58%	277	157	57%
Used condom at last sex - direct question	289	245	85%	290	249	86%	290	144	50%	278	140	50%
Used condom at last sex - list experiment	578	-	81%	580	-	80%	580	-	44%	556	-	46%

9. Table 2 - it would be useful to see whether the rates differed by randomised group. If so this may lead to more prospect of bias due to attrition (although the attrition rate is not that high, so I wouldn't be too worried about this).

We have added the difference in attrition between randomisation groups for both strata and pooled. There is no differential attrition between the treatment and control group for the pooled results and for the commercial sex strata. There is a small but statistically significant differential attrition between treatment and control for the transactional sex strata. Note that 91% of the treatment group and 96% of the control group was followed up in the transactional sex strata.

Further investigation of the causes of attrition shows that there is no difference in risky sexual behaviours at baseline between those who attrit and those who did not attrit among the transactional sex strata. Given the very low attrition rate in the transactional sex strata and the absence of selection issue, we do not believe this poses a threat to our findings.

We have also added Table 2 below.

Table 2: Attrition

	**Pooled**		
	Combined	Treatment	Control
Baseline & Randomisation	1147 (100%)	568 (100%)	579 (100%)
Midline	956 (83%)	478 (84%)	478 (83%)
Endline	805 (70%)	394 (69%)	411 (71%)
Midline or Endline	1020 (89%)	510 (90%)	510 (88%)
	**Transactional**		
	Combined	Treatment	Control
Baseline & Randomisation	568 (100%)	290 (100%)	278 (100%)
Midline	493 (87%)	246 (85%)	247 (89%)
Endline	403 (71%)	193 (67%)	210 (76%)
Midline or Endline	530 (93%)	263 (91%)	267 (96%)
	**Commercial**		
	Combined	Treatment	Control
Baseline & Randomisation	579 (100%)	289 (100%)	290 (100%)
Midline	463 (80%)	232 (80%)	231 (80%)
Endline	402 (69%)	201 (70%)	201 (69%)
Midline or Endline	489 (84%)	246 (85%)	243 (84%)

We have now added this paragraph to the manuscript:

[X]"In **Error! Reference source not found.**, shows the response rates in each arm and strata of the study. The attrition rate for those that did not return for either midline or endline was very low, 7% for the transactional sex strata and 16% for the commercial sex strata. We find a statistically significant difference in attrition between the treatment and the control group in the transactional strata: the control arm was 5 percentage points more likely to return for follow up than the treatment arm. We show that there was no difference in risky sexual behaviours at baseline among participants who attrit and participants who did not attrit in the transactional sex strata, see table A10 in the appendix.

Table A10: Difference between baseline characteristics of attritors in the transactional sex strata

	Returned	Attrited	Difference	p-val
Part of the intervention group	0.496	0.711	0.214**	0.011
Age, years	24.830	23.579	-1.251	0.209
Experience - months in sex work	37.481	36.838	-0.643	0.923
Nationality - Cameroon	1.000	0.974	-0.026***	0.000
Education - Primary	0.077	0.053	-0.025	0.579
Education - Secondary primary cycle	0.236	0.395	0.159**	0.028
Education - Secondary second cycle	0.360	0.316	-0.045	0.580
Education - Superior	0.313	0.184	-0.129*	0.095
Education - None	0.006	0.053	0.047***	0.003
Education - Other	0.008	0.000	-0.008	0.592
Currently in school	0.474	0.368	-0.105	0.210
Never married	0.974	1.000	0.026	0.311
Separated	0.019	0.000	-0.019	0.394
Divorced	0.000	0.000	0.000	-
Widowed	0.008	0.000	-0.008	0.592
Number of economic dependents	2.926	3.053	0.126	0.616
Have at least one child economic dependents	0.843	0.868	0.025	0.681
Number of biological children	1.189	1.342	0.153	0.508
Household size	5.719	4.816	-0.903*	0.069
Household expenditure per adult equivalent (CFAF)	36173	30487	-5686	0.426
Household expenditure per adult equivalent (log CFAF)	10.249	10.187	-0.062	0.552
Used condom at last sex - direct question	0.492	0.605	0.113	0.180
Self-reported level of risky sexual behaviour (0/10)	5.521	5.658	0.137	0.750
Observations	530	38	568	-

We however now cite this as a limitation in the discussion section:

[Discussion, paragraph 7] "A final potential limitation of the study is differential attrition between the treatment and control groups in the transactional sex strata. However, assuming that all participants in the treatment arm of the transactional sex strata were treated for HIV and assuming a conservative HIV incidence equal to the one in the control arm, it would result in less than one additional HIV infection in the treatment arm. Running the unadjusted logistic regression by adding an additional HIV infection in the treatment arm yields only a small reduction in estimated impact that remains statistically significant at 5% (OR = 0.199 (95% CI[0.042,0.930]; p = 0.040)."

10. Statistical analysis "we conducted an intention-to-treat (ITT) analysis where respondents were analysed based on their randomised study group" - I would probably refer to this as modified ITT or ITT with complete cases to account for the fact that individuals with missing response were not included.

Thank you for this observation. We have now modified this:

[Statistical analysis] "To assess the effect of health insurance on our primary and secondary outcomes, we conducted a modified intention-to-treat (ITT) analysis where respondents were analysed based on their randomised study group but those with missing responses were not included."

11. Statistical analysis "We report the marginal effect for all other binary outcomes" - I wasn't quite sure what this means in comparison to the primary analysis results - can it be clarified? I presume it means the estimated difference in proportions rather than the OR. The methods section implies binary secondary outcomes would be analysed with a method for continuous outcomes but the footnote for table 4 says something different. I would recommend clarifying this in the methods section.

We have now updated the methods section:

[Statistical analysis] "We estimate logistic regressions with odds ratios (OR) for our primary biological and all binary outcomes reporting the estimated difference in proportions or estimated marginal effect. We estimate the marginal effects using ordinary least squares (OLS) for continuous outcomes. Table notes indicate which outcomes were estimated via which method."

*12. Table 4: the * and ** are not defined from what I can see - presumably p<0.05 and p<0.01; I would generally recommend the exact p-values are provided.*

Thank you for spotting this omission. All relevant tables now have star definitions included.

13. Page 17: I didn't follow the analysis used for results in Table A4 - if the authors are adjusting for post-baseline terms in a linear regression then I do not think this is the correct way to investigate whether one variable mediates another.

We fully agree with this comment and made a mistake. We have removed this model from table A4 and updated the interpretation detailed below:

	Unadjusted			Adjusted		
	n	ME	95% CI	n	AME	95% CI
**<u>Trasnactional Sex</u>**						
HIV - removed STI symptoms	284	-0.034**	-0.065, -0.003	284	-0.035**	-0.066, -0.005

Significance levels: * p<0.1, ** p<0.05, *** p<0.01
Notes: Estimated using linear probability model (OLS) since Maximum likelihood estimation used for logistic regression did not converge. Unadjusted = compares outcomes of follow-up data without adjustment. Adjusted = adjusts for baseline levels of the outcome, if the outcome was collected during midline, then baseline controls, namely log of adult equivalent expenditure, number of economic dependents, household size and if the respondent has a child. There are not enough observations to estimate on a sub-sample of those displaying possible STI symptoms

[Results, subsection 3]"In addition, **Error! Reference source not found.** displays results from models when removing those with STI symptoms, finding the impact of the intervention still reduces HIV by around 3.4 percentage points when conditioning on STI symptoms (ME = -

0.034 (95% CI[-0.065,-0.003]); p = 0.029). The estimates of reported STI symptoms are low, so caution must be exercised when interpreting them. However, they suggest that the intervention is not acting through the STI treatment channel."

James Wason

Reviewer #3:

1) *The eligibility criteria considered engagement in either transactional or commercial sex (see pg. 5), which seems to influence the results - showing positive outcomes in one category and none in the other. The reader may wonder how this study defined/differentiated the two in an African context (Yaoundé).*

Thank you for these comments, we agree that the nuance differentiating transactional sex from sex work were not clear in the first version of the paper. We have gone to effort to help differentiate and provide more details on the differences. In response to comments 1 and 2 we have updated the paragraph on participants recruitment:

[Recruitment section] "A stratified randomised controlled trial was conducted in Yaoundé, Cameroon, a country chosen due to having the highest HIV gender gap in the world. The sample was stratified 1:1 by the type of risky sexual activity: commercial or transactional sex. Two community-based organisations (CBOs) were identified, each exclusively working exclusively with one of the populations. It is worth noting that there is a fuzzy boundary between commercial and transactional sex [11]. The initial categorisation was based on peer and self-identification, which was then verified by research team members according to the types of relationships in which the participants engaged. The research team members in charge of the recruitment were trained to categorise participants based on the following definitions. Transactional sex was defined as non-commercial, non-marital sexual relationships motivated by the expectation that sex will be exchanged for material help, gifts, support or other benefits. Transactional sex may sometimes involve the exchange of money but is less systematic and explicit than commercial sex. Women who engage in transactional sex do not identify themselves as FSWs [15]. Commercial sex was defined as sexual relationships in which the acts are pre-negotiated, immediately remunerated and discreet, and are not in the context of romantic/emotional relationships, but rather are conceived and perceived in terms of the "exchange of goods" in a professional environment. Sex workers often identify themselves as such [16].

To minimise the risk of intervention spillover, only one person per household was recruited. The CBO 'Renata' was selected to recruit and follow women who engage in transactional sex, while 'Horizon Femmes' was chosen for their expertise in working with FSWs. These sites were used for data collection, and the research team collaborated with CBO staff who interacted with the study participants throughout the study. Snowball sampling was used to recruit participants from both commercial and transactional sex groups, as these populations, particularly FSWs, are often hidden due to the illegality of sex work in Cameroon [12]. Women were instructed to recruit only others in their strata. In cases where there was uncertainty, the research teams categorised based on relationship type. If a woman reported having at least one client in addition to sugar daddies, she was allocated to the commercial sex strata.

The CBO identified 34 'seeds' for the transactional sex strata and 23 'seeds' for the commercial sex strata. Seeds had a large network within their community. Each of these

seeds then recruited three participants who in turn were asked to recruit up to three other peers. This process was repeated over four waves of recruitment. An incentive of FCFA 500 (£0.64) was provided for each participant recruited who met the eligibility criteria. On average, 21 transactional and 31 commercial women were recruited per seed, resulting in a total sample of 1,506 participants for the study."

2) Following the above concern, it is important to state - where such definitions come from to avoid imposition and epistemic injustices that have historically characterised global health practice.

We share your concern. Note that the definitions came from the literature and were discussed extensively with staff involved in the study in Cameroon during training. We analysed those definitions in the context of Cameroon and the definitions used were in line with the context. We also used local term to define sugar daddy during recruitment (i.e. cou plié, sponsor).

3) It is interesting that the intervention allowed women to leave transactional sex and an increase in condom use and reduction in sex acts for those who remained in TS (pg. 15). However, these results are based on self-reports. Thus, they cannot form a solid basis for policy recommendations. Adding caution as part of the study's limitation can help clear some of these concerns.

We have added this line to the discussion:

[Discussion paragraph 5] "Secondary outcomes were primarily self-reported, with the exception of condom use by the list experiment, which was indirectly elicited using a list experiment. It is important to note that self-reported outcomes may be subject to measurement error and should therefore be interpreted with caution."

***Reviewer #4**: This is an interesting and important paper that explores the impact of protection from economic shocks on HIV prevention amongst commercial sex workers and women engaged in transactional sex in Cameroon. The paper contributes to the growing literature on women at the edge of sex work but who do not identify as selling sex and emphasises the importance of their inclusion within HIV prevention strategies including PrEP. The paper also suggests that protection from economic health shocks is likely to be of benefit in preventing HIV amongst women involved in transactional sex, but less protective for female sex workers, a finding that is not unsurprising but this is the first time a randomised trial has shown demonstrated this. I have some minor comments in relation to the methods and some suggestions where further clarification might be helpful to the reader.*

Thank you very much for your insightful and well through out comments. We are grateful to use your expertise to help improve the paper. We have addressed all your comments and added details below each of them where relevant.

We have added some additional discussion of why we felt the intervention did not work for the female sex workers:

"As there were fewer HIV infections among FSWs than expected, the trial was underpowered to detect the effect of the intervention on HIV incidence among FSWs. We do not find comparable reductions in self-reported risky sexual behaviour at the intensive and extensive margins among FSWs, suggesting that the intervention did not reduce risky sexual behaviours, but this must be treated with caution. Although this paper cannot provide a complete explanation, we believe that the potentially heterogeneous impact could be explained by differences between the commercial and transactional sex contexts.

First, we believe that the extent to which women are entrenched in their activities differs. FSWs face greater barriers to exit than women in transactional sex. This is because they are more likely to depend on sex work for income, which is higher (93,000 CFAF vs. 44,000 CFAF per month) and are less likely to have a second job as a result (31% vs. 45%) or be enrolled in school (16% vs. 46%) than women engaging in transactional sex. This means that the magnitude of the economic impact of the intervention is unlikely to be sufficient to force FSWs out of the activity. In addition, given that healthcare costs account for a significant proportion of healthcare expenditure for women engaging in transactional sex (63% compared to 34% for FSWs), if a sugar daddy were to offer to pay for medical bills, the removal of these costs for women could be enough to end such activity.

Second, the intervention was less effective because HIV knowledge was higher among FSWs, which leads to less scope for behaviour change. FSWs are considered as a "key population" by UNAIDS [13] and in Cameroon they have access to PrEP, in our sample of FSWs 13% had ever taken PrEP by endline while none of the women engaging in transactional sex were using PrEP at endline. In addition, over 90% of FSWs had been tested for HIV in the last 12 months, compared with only 50% of women in transactional sex (Table 1). Third, women in transactional sex engage in riskier sexual behaviour, albeit with fewer partners (Table 1), and may therefore be more responsive to the intervention. In a companion paper, we show that trust and implied future support (compared to immediate payment for sex workers) are part

of the payment system for transactional sex and may explain the higher levels of risky behaviour [14].

Third, stigma from health workers towards FSWs is a main barrier to health care use and leads to self-stigma [42]. This may explain why there was no increase in the likelihood of visiting a professional among FSWs (ME = 0.039 (95% CI[-0.063,0.140]); p = 0.455) than among women who engage in transactional sex (ME = 0.126 (95% CI[0.032,0.219]); p = 0.008). This issue may limit the effect of hospital-based interventions for FSWs.

Whilst I recognise the Respondent Driven Sampling approach has been detailed elsewhere in a UCL protocol publication, I would still have liked to have seen some further clarification on the method within this paper, both in terms of its potential limitations within the discussion, and to strengthen the reader's conviction in this method to recruit participants within the trial - perhaps by presenting recruitment trees to demonstrate heterogeneity in the sample within supplemental files?

Thank you for this comment. We have added more detail to the recruitment section of the paper detailing this process alongside some important inclusion, exclusions criteria as well as detailed definition of commercial and transactional sex used in the study. The recruitment tree can be found in Appendix Figure A3 and is copied below:

The updated recruitment section:

[X] "

A stratified randomised controlled trial was conducted in Yaoundé, Cameroon, a country chosen due to having the highest HIV gender gap in the world. The sample was stratified 1:1 by the type of risky sexual activity: commercial or transactional sex. Two community-based organisations (CBOs) were identified, each exclusively working exclusively with one of the populations. It is worth noting that there is a fuzzy boundary between commercial and transactional sex [11]. The initial categorisation was based on peer and self-identification, which was then verified by research team members according to the types of relationships in which the participants engaged. The research team members in charge of the recruitment were trained to categorise participants based on the following definitions. Transactional sex was defined as non-commercial, non-marital sexual relationships motivated by the expectation that sex will be exchanged for material help, gifts, support or other benefits. Transactional sex may sometimes involve the exchange of money but is less systematic and explicit than commercial sex. Women who engage in transactional sex do not identify themselves as FSWs [15]. Commercial sex was defined as sexual relationships in which the acts are pre-negotiated, immediately remunerated and discreet, and are not in the context of romantic/emotional relationships, but rather are conceived and perceived in terms of the "exchange of goods" in a professional environment. Sex workers often identify themselves as such [16].

To minimise the risk of intervention spillover, only one person per household was recruited. The CBO 'Renata' was selected to recruit and follow women who engage in transactional sex, while 'Horizon Femmes' was chosen for their expertise in working with FSWs. These

sites were used for data collection, and the research team collaborated with CBO staff who interacted with the study participants throughout the study. Snowball sampling was used to recruit participants from both commercial and transactional sex groups, as these populations, particularly FSWs, are often hidden due to the illegality of sex work in Cameroon [12]. Women were instructed to recruit only others in their strata. In cases where there was uncertainty, the research teams categorised based on relationship type. If a woman reported having at least one client in addition to sugar daddies, she was allocated to the commercial sex strata.

The CBO identified 34 'seeds' for the transactional sex strata and 23 'seeds' for the commercial sex strata. Seeds had a large network within their community. Each of these seeds then recruited three participants who in turn were asked to recruit up to three other peers. This process was repeated over four waves of recruitment. An incentive of FCFA 500 (£0.64) was provided for each participant recruited who met the eligibility criteria. On average, 22 women engaged in transactional sex and 33 engaged in commercial sex were recruited per seed, resulting in a total sample of 1,506 participants for the study.

To be eligible for the study, participants had to engage in transactional or commercial sex in Yaoundé, Cameroon, be female, aged 15 years or older, not be married, have at least one economic dependent living in Yaoundé (no family relationship required), be HIV-negative, have a password-protected phone and be able to respond to text messages. STI testing was conducted at baseline, with free treatment offered to those who tested positive, referral for ART initiation to those who tested positive for HIV, and referral to the CBO if the participant was severely depressed, a victim of physical violence, or had recently been raped.

Minors, defined as women recruited under the age of 21, were included in the study. To protect their privacy and avoid negative consequences, the ethics committees allowed them to waive parental consent and obtain it from an adult representative of the CBOs. More information can be found in the study protocol [17]."

We have also added the following limitation to the discussion:

[Discussion, paragraph 7] "The sample used in our study may be subject to bias due to the limitations of the snowball methodology. Specifically, the seeds used by the CBO who initiated our recruitment chains may have led to the exclusion of participants with certain characteristics. As a result, the generalizability of our findings to the wider population of women engaged in transactional and commercial sex may be limited. Note that this recruitment strategy was the only option available, given that both strata were hidden populations."

The recruitment tree / consort diagram was added in the appendix as Figure A4

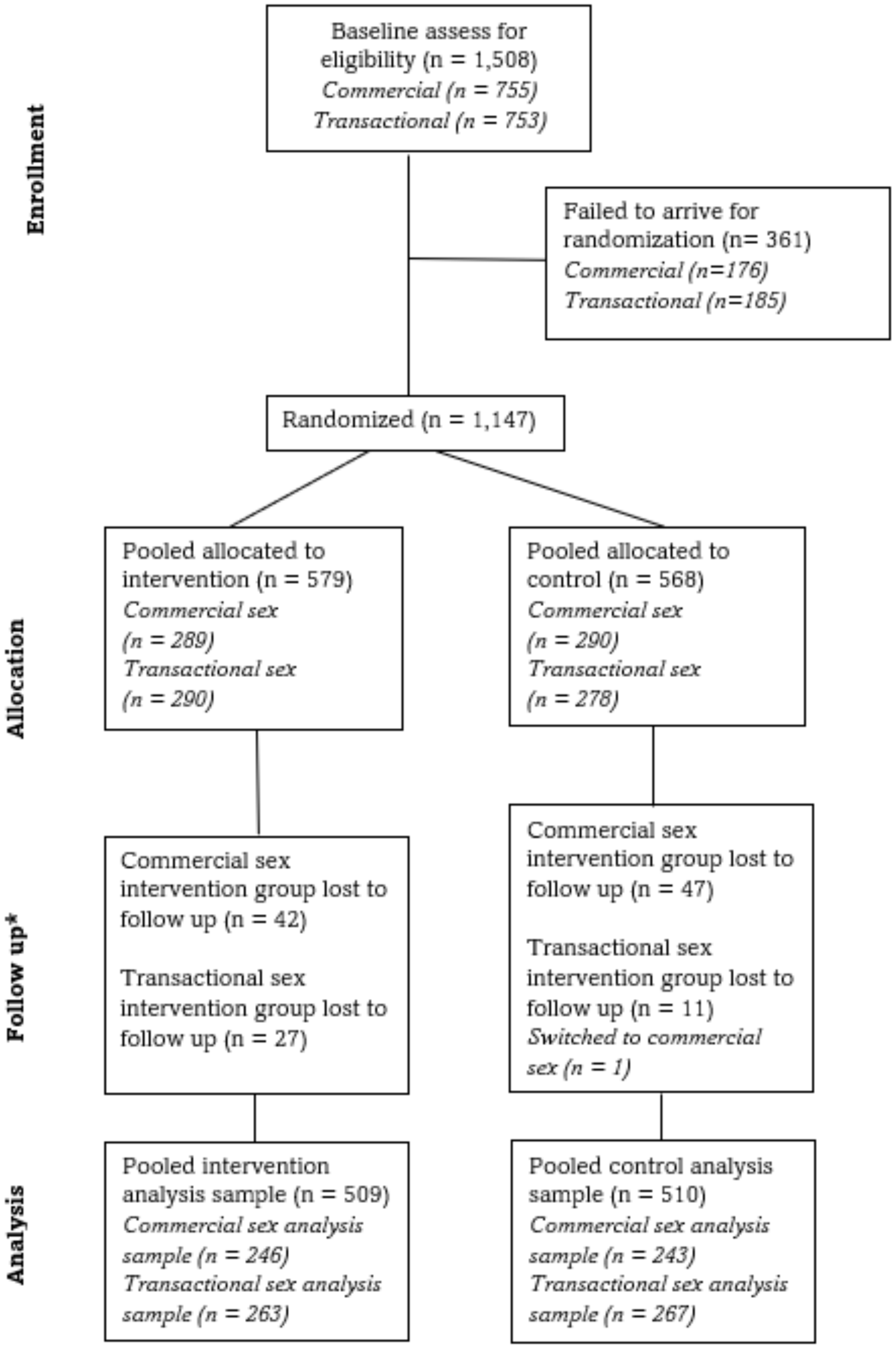

* Follow up defined as attended either midline or endline. Lost to follow up are those that missed both follow up surveys

- *Linked to this I wondered whether seeds identified through local CBOs had social networks that extended and crossed over between women involved in transactional and commercial sex. Further detail on the recruitment process from each seed would have been useful.*

We have updated the paper with further details in the recruitment paragraphs. Note that we did not allow for women to recruit women who engaged in the other strata of the survey. This would only occur if a participant engaged in both activities, in which case we would consider her as a female sex worker. The research team was careful to allocate each participant to the correct strata using clear definitions. We have now added these definitions in the text. If someone was mis-categorised by our definitions, they were moved into the other group. Instances of this were very low.

We also have now also added this in the manuscript:

[Recruitment] "Note that if a participant was found to engage in both commercial and transactional sex, they will be considered as a female sex worker".

- *The authors present the terms commercial and transactional uncritically as categorisation that forms the basis of their analysis but raise the fact that there are issues in terms of reaching women involved in transitional sex in the discussion. It might have been helpful if they had included a clear working definition for this study within the Yaounde context.*

We have now added the following:

[Recruitment] "A stratified randomised controlled trial was conducted in Yaoundé, Cameroon, a country chosen due to having the highest HIV gender gap in the world. The sample was stratified 1:1 by the type of risky sexual activity: commercial or transactional sex. Two community-based organisations (CBOs) were identified, each exclusively working exclusively with one of the populations. It is worth noting that there is a fuzzy boundary between commercial and transactional sex [11]. The initial categorisation was based on peer and self-identification, which was then verified by research team members according to the types of relationships in which the participants engaged. The research team members in charge of the recruitment were trained to categorise participants based on the following definitions. Transactional sex was defined as non-commercial, non-marital sexual relationships motivated by the expectation that sex will be exchanged for material help, gifts, support or other benefits. Transactional sex may sometimes involve the exchange of money but is less systematic and explicit than commercial sex. Women who engage in transactional sex do not identify themselves as FSWs [15]. Commercial sex was defined as sexual relationships in which the acts are pre-negotiated, immediately remunerated and discreet, and are not in the context of romantic/emotional relationships, but rather are conceived and perceived in terms of the "exchange of goods" in a professional environment. Sex workers often identify themselves as such [16].."

- *They also don't identify the potential relationality between transactional and commercial sex and the fact that these 'categories' are actually on a continuum in*

terms of identity, behaviour and risk exposure. Using these as distinct categories uncritically within the methodology and presentation of the results might need to be discussed within the Discussion section.

We have included more detailed definitions in the introduction that speaks to the continuum between transactional and commercial sex, highlighting the key differences and similarities for readers a the outset, see previous comment.

We also added some additional explanation as to why we believe the intervention worked for women in transactional but not for women engaged in commercial sex. See below the new section in the discussion:

[Discussions from paragraph 2] "As there were fewer HIV infections among FSWs than expected, the trial was underpowered to detect the effect of the intervention on HIV incidence among FSWs. We do not find comparable reductions in self-reported risky sexual behaviour at the intensive and extensive margins among FSWs, suggesting that the intervention did not reduce risky sexual behaviours, but this must be treated with caution. Although this paper cannot provide a complete explanation, we believe that the potentially heterogeneous impact could be explained by differences between the commercial and transactional sex contexts.

First, we believe that the extent to which women are entrenched in their activities differs. FSWs face greater barriers to exit than women in transactional sex. This is because they are more likely to depend on sex work for income, which is higher (93,000 CFAF vs. 44,000 CFAF per month) and are less likely to have a second job as a result (31% vs. 45%) or be enrolled in school (16% vs. 46%) than women engaging in transactional sex. This means that the magnitude of the economic impact of the intervention is unlikely to be sufficient to force FSWs out of the activity. In addition, given that healthcare costs account for a significant proportion of healthcare expenditure for women engaging in transactional sex (63% compared to 34% for FSWs), if a sugar daddy were to offer to pay for medical bills, the removal of these costs for women could be enough to end such activity.

Second, the intervention was less effective because HIV knowledge was higher among FSWs, which leads to less scope for behaviour change. FSWs are considered as a "key population" by UNAIDS [13] and in Cameroon they have access to PrEP, in our sample of FSWs 7.5% had ever taken PrEP which rises to 13% currently taking PrEP by endline. No women in transactional sex are using PrEP at endline. In addition, over 90% of FSWs had been tested for HIV in the last 12 months, compared with only 50% of women in transactional sex (Table 1). Third, women in transactional sex engage in riskier sexual behaviour, albeit with fewer partners (Table 1), and may therefore be more responsive to the intervention. In a companion paper, we show that trust and implied future support (compared to immediate payment for sex workers) are part of the payment system for transactional sex and may explain the higher levels of risky behaviour. [14].

Thirdly, stigma from health workers towards FSWs is a main barrier to health care use and leads to self-stigma [42]. This may explain why there was a smaller increase in health care use (Xpts versus Xpts) among FSWs than among women who engage in transactional sex who

received the health insurance. This issue may limit the effect of hospital-based interventions for FSWs."

"

- *One small final point - what proportion of the sample were under 21? How feasible was it obtaining parental consent for younger women engaged in transactional or commercial sex work? Why was this approach used? I wonder if it impacted the recruitment?*

Thanks you for noting this omission. We have included this comment by adding the following paragraph to the recruitment paragraph:

[Recruitment paragraph 5]"Minors, defined as women recruited below the age of 21, were included in the study. To protect their privacy and prevent any negative consequences, ethics committees allowed for parental consent to be waived and obtained from an adult representative of the CBOs. Further information can be found in the study protocol. [31]."

- *Other than these minor comments and points of clarification the paper is well described, the study has been well conducted and addresses an important potential contribution to addressing risk behaviour exacerbated through health shocks, particularly for women engaged in transactional sex and should be considered for publication.*

Thank you very much for this comment.

- *The authors might also like to consider the potential impact of the bias towards self-selection since the analysis was based only on women who returned for randomisation.*

In the recruitment section, we have now included the following information. It should be noted that randomising participants at baseline may have resulted in a higher attrition rate. However, it is important to mention that by randomising only returning participants, we may reduce the generalisability of the results.

[Randomising and blinding] "By completing randomisation after baseline only on returning participants, we have reduced attrition rate but we may have reduced the generalisability of pour results."

Requests from the ***editors (and journal requirements):***

*****Please note: not all will apply to your paper, but please check each item carefully*

ACADEMIC EDITOR COMMENTS

The central reasons for needing a revision are to unpack the rationale for the study, put this in context of what is known, clarify that due to the design the findings may not be generalizable, but there is enough to work to understand the mechanism better. Alternative interventions could be considered in future trials such as guaranteed basic income. The term "shock" in this context was new to me so could be explained and the abstract edited to include a definition.

Thank you very much for the opportunity to improve our paper and to resubmit given yours and the reviewers expertise. Throughout we have updated the language and structure to improve the clarity of our argument including definitions and explanations.

We have updated the *background* part of the abstract to say:

"**Background**: Women in sub-Saharan Africa are disproportionately affected by the HIV epidemic. Young women are twice as likely to be living with HIV as men of the same age and account for 64% of new HIV infections among young people. Many studies suggest that financial needs, alongside biological susceptibility, are a leading cause of the gender disparity in HIV acquisition. New robust evidence suggests women adopt risky sexual behaviours to cope with economic shocks, the sudden falls in household's income or consumption power, enhancing our understanding of the link between poverty and HIV. We test if health insurance protects against economic shocks, reducing the need for vulnerable women to engage in risky sexual behaviours and reducing HIV incidence."

We have also added the definition of economic shocks in the Introduction:

[Introduction, paragraph 2] "Not only that, the network of male partners can result in other financial or in-kind gifts used to cope with economic shocks, defined as 'An unexpected, sudden and significant change in income or expenditure of a household, which if unaddressed would have meaningful impacts on household consumption' [12,13]. ."

All of the general comments have been addressed with details provided where relevant.

GENERAL COMMENTS

1) Please include page numbers and line numbers in the manuscript file. Use continuous line numbers (do not restart the numbering on each page).

2) Please cite the reference numbers in square brackets. Citations should be preceding

punctuation.

FINANCIAL DISCLOSURE

The funding statement should include: specific grant numbers, initials of authors who received each award, URLs to sponsors' websites. Also, please state whether any sponsors or funders (other than the named authors) played any role in study design, data collection and analysis, the decision to publish, or preparation of the manuscript. If they had no role in the research, include this sentence: "The funders had no role in study design, data collection and analysis, decision to publish, or preparation of the manuscript."

The financial disclosure has been included.

COMPETING INTEREST

All authors must declare their relevant competing interests per the PLOS policy, which can be seen here: https://eur01.safelinks.protection.outlook.com/?url=https%3A%2F%2Fjournals.plos.org%2Fplosmedicine%2Fs%2Fcompeting-interests&data=05%7C02%7Ca.lepine%40ucl.ac.uk%7Cb16dd0b10a8441c7f42c08dc3867034c%7C1faf88fea9984c5b93c9210a11d9a5c2%7C0%7C0%7C638447262610998722%7CUnknown%7CTWFpbGZsb3d8eyJWIjoiMC4wLjAwMDAiLCJQIjoiV2luMzIiLCJBTiI6Ik1haWwiLCJXVCI6Mn0%3D%7C0%7C%7C%7C&sdata=knf0AceO89GmrQzoQmf%2Bx44Ydj3Sk95gCKBggQdHlsc%3D&reserved=0

For authors with ties to industry, please indicate whether any of the interests has a financial stake in the results of the current study. All authors declare no competing interests and we have added a statement in the paper

"No authors have competing interests."

TITLE

Please revise your title according to PLOS Medicine's style. Your title must be nondeclarative and not a question. It should begin with main concept if possible. "Effect of" should be used only if causality can be inferred, i.e., for an RCT. Please place the study design ("A randomized controlled trial," "A retrospective study," "A modelling study," etc.) in the subtitle (ie, after a colon).

We used the name of the registered study as a title. Causality is inferred so we used the word 'effect' and the study design is there.

"The effect of protecting women against economic shocks to fight HIV in Africa: Evidence from the POWER randomized controlled study in Cameroon"

ABSTRACT

1) Please report your abstract according to CONSORT for abstracts, following the PLOS Medicine abstract structure (Background, Methods and Findings, Conclusions). https://eur01.safelinks.protection.outlook.com/?url=https%3A%2F%2Fwww.equator-network.org%2Freporting-guidelines%2Fconsort-abstracts%2F&data=05%7C02%7Ca.lepine%40ucl.ac.uk%7Cb16dd0b10a8441c7f42c08dc3867034c%7C1faf88fea9984c5b93c9210a11d9a5c2%7C0%7C0%7C638447262611003815%7CUnknown%7CTWFpbGZsb3d8eyJWIjoiMC4wLjAwMDAiLCJQIjoiV2luMzIiLCJBTiI6Ik1haWwiLCJXVCI6Mn0%3D%7C0%7C%7C%7C&sdata=HqvLeif6dP2mp134hgsxiXdWPg0cDwOTPkg2Jts2bKQ%3D&reserved=0

The abstract now meet these requirements.

2) Please note that trial abstracts should only report secondary outcomes if all secondary outcomes of the trial are included. For trials that have many secondary outcomes, the abstract should be limited to reporting the primary outcome.

We have removed specific mention of secondary outcomes in the results section of the abstract and focussed only on primary outcomes. We do summarise the findings from the mechanism investigation.

The new abstract now reads:

Background: Women in sub-Saharan Africa are disproportionately affected by the HIV epidemic. Young women are twice as likely to be living with HIV as men of the same age and account for 64% of new HIV infections among young people. Many studies suggest that financial needs, alongside biological susceptibility, are a leading cause of the gender disparity in HIV acquisition. New robust evidence suggests women adopt risky sexual behaviours to cope with economic shocks, the sudden falls in household's income or consumption power, enhancing our understanding of the link between poverty and HIV. We test if health insurance protects against economic shocks, reducing the need for vulnerable women to engage in risky sexual behaviours and reducing HIV incidence.

Method & Findings: We conducted a randomised controlled trial to test the effectiveness of a formal shock coping strategy to prevent HIV among high-risk women (registration number: ISRCTN 22516548). We recruited 1,506 adolescent girls and women involved in transactional sex or commercial sex in Yaounde, Cameroon, using snowball sampling. Half of the study participants were randomly assigned to receive free health insurance for themselves and their economic dependents for 12 months. The control group received the intervention at the end of the study. We collected data on socioeconomics, health and sexual behaviour and our primary outcomes of sexually transmitted infections (STIs) and HIV biomarkers at baseline, 6 months after randomisation (midline) and 12 months after randomisation (endline). We found that study participants who engaged in transactional sex and were

assigned to the treatment group were less likely to become infected with HIV (odds ratio (OR)=0.109 (95% confidence interval (CI)[0.014,0.870]); p=0.036). There was no evidence of a reduction in HIV incidence among female sex workers. There was also no effect on STI acquisition. The main limitations of this study were the challenges of collecting reliable biomarkers for STIs and the lower incidence of HIV in the FSW group, which prevented detection of study effects.

Conclusion: The study provides the first evidence of the effectiveness of a formal shock strategy for HIV prevention among women who engage in transactional sex in Africa. The effectiveness of the intervention is mediated by a reduction in out-of-pocket payments rather than an increase in health care utilisation, which showed only small increases. The study suggests that policymakers in Africa should consider implementing formal shock strategies for HIV prevention among women who engage in transactional sex, taking into account the high-risk nature of this activity.

3) PLOS Medicine requests that main results are quantified with 95% CIs as well as p values. When reporting p values please report as p<0.001 and where higher as the exact p value p=0.002, for example. For the purposes of transparent data reporting, if not including the aforementioned please clearly state the reasons why not. When a p value is given, please specify the statistical test used to determine it.

We have updated the paper throughout to fit these requirements.

4) Throughout, suggest reporting statistical information as follows to improve clarity for the reader "22% (95% CI [13%,28%]; p</=)". Please be sure to define all numerical values at first use. Please amend throughout the abstract and main manuscript (including tables). Please note the use of commas to separate upper and lower bounds, as opposed to hyphens as these can be confused with reporting of negative values.

We have updated the paper throughout to fit these requirements.

5) Please ensure that all numbers presented in the abstract are present and identical to numbers presented in the main manuscript text.

We have replaced all numbers in the abstract to be consistent.

6) Please include the study design, population and setting, number of participants, years during which the study took place (enrollment and follow up), length of follow up, and main

outcome measures.

We have added reference to Figure A3 which is a detailed project timeline. We also detail why there was a delay between randomisation and the intervention starting.

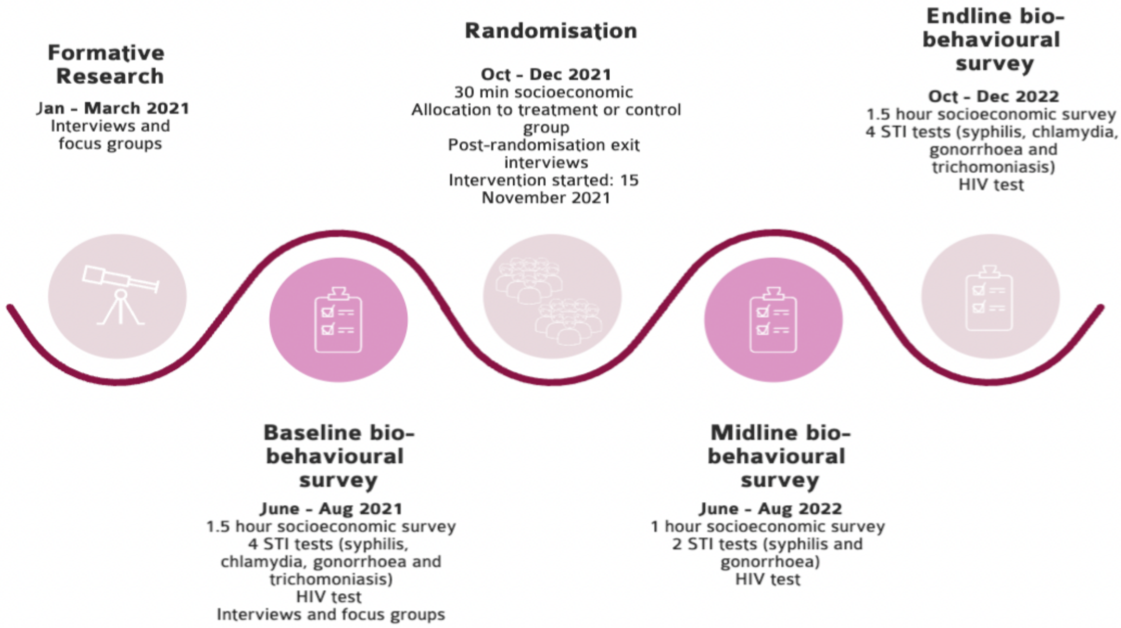

7) Please specify who was blinded to the intervention and control, define the intervention and control states, provide the number in each group, state that analysis was intention to treat and provide the number of participants lost to follow up in each group.

We have added authors initials to indicate who was blinded and to what prior to the paper being written.

"Authors, HC, AL leading analysis were blinded to the treatment allocation of respondents. All analyses have been conducted in STATA version 17 and follow the analysis specified in the study protocol [17] and registered on ISRCTN (study 225165484)."

8) Please include the important dependent variables that are adjusted for in the analyses.

These are included below each table that has models including them.

"Adjusted = adjusts for baseline levels of the outcome, if the outcome was collected during midline, then baseline controls, namely log of adult equivalent expenditure, number of economic dependents, household size and if the respondent has a child."

9) Please define all abbreviations including those for statistical reporting at first use.

All abbreviations are spelled out at their first use.

10) Abstract Background: Provide the context of why the study is important. The final sentence should clearly state the study question.

We have addressed this, please see the abstract above.

11) In the last sentence of the Abstract Methods and Findings section, please describe the main limitation(s) of the study's methodology.

We have added this:

[Abstract] "The main limitations of this study were the challenges of collecting reliable biomarkers for STIs and the lower incidence of HIV in the FSW group, which prevented detection of study effects."

12) Abstract Conclusions: Please address the study implications without overreaching what can be concluded from the data and interpret the study based on the results presented in the abstract, emphasizing what is new without overstating your conclusions.

The conclusion of the abstract has been updated accordingly. Please see the updated abstract:

[Abstract] **Conclusion**: The study provides the first evidence of the effectiveness of a formal shock strategy for HIV prevention among women who engage in transactional sex in Africa. The effectiveness of the intervention is mediated by a reduction in out-of-pocket payments rather than an increase in health care utilisation, which showed only small increases. The study suggests that policymakers in Africa should consider implementing formal shock strategies for HIV prevention among women who engage in transactional sex, taking into account the high-risk nature of this activity.

13) Please include the clinical trial registry number in the abstract.

We have now included the ISRCTN number

AUTHOR SUMMARY

An author summary has been added to the manuscript copied below:

Author Summary

Why Was This Study Done? Authors should reflect on what was known about the topic before the research was published and why the research was needed.

- Women in Africa, especially adolescents girls and young women, suffer a greater burden of new HIV infections than men.

- Evidence suggests that women may engage in risky sexual behaviours, including unprotected sex, to earn additional money when faced with high expenses or low income also known as economic shocks.
- One of the most common economic shocks is health out of pocket payments which can be dramatically reduced with health insurance.

What Did the Researchers Do and Find? Authors should briefly describe the study design that was used and the study's major findings. Do include the headline numbers from the study, such as the sample size and key findings.

- We conducted a randomized control trial assessing the impact of health insurance as a way of protecting women engaging in commercial sex and in transactional sex from these economic shocks to examine to prevent HIV infection.
- We recruited 1,508 women, the sample was stratified depending on whether the women is a female sex worker (n=755)or engage in transactional sex (n=753)
- We randomised 1,147 with 579 allocated to the treatment (commercial n = 289, transactional n = 290) and 568 allocated to the control (commercial n = 290, transactional n=278) who received the intervention after the conclusions of the study.
- We found that health insurance almost eliminated fully out-of-pocket payments and led to a small increase in health care use.
- The effect of the intervention on primary outcomes was large among women engaging in transactional sex. We found that health insurance reduced the incidence of HIV 8 fold (OR=0.109, p-value<0.05). There was no effect of the intervention in the commercial sex strata.

• What Do These Findings Mean? Authors should reflect on the new knowledge generated by the research and the implications for practice, research, policy, or public health. Authors should also consider how the interpretation of the study's findings may be affected by the study limitations. In the final bullet point of 'What Do These Findings Mean?', please describe the main limitations of the study in non-technical language.

- These findings show that preventing economic shocks among women engaging in transactional sex in Africa is an effective HIV prevention strategy.
- The results also highlighted that women in transactional sex were more affected by HIV than female sex workers, which should required some attention from policymakers.

METHODS AND RESULTS

1) PLOS Medicine requests that main results are quantified with 95% CIs as well as p values. We suggest reporting statistical information as detailed above – see under ABSTRACT

These have included throughout.

2) Please present numerators and denominators for percentages (at least in the Tables [not necessarily each time they're mentioned]).

We have updated table 1 to include both numerator and denominator. Please see table 1 below:

	Commercial Sex						Transactional Sex					
	Treatment			Control			Treatment			Control		
	Obs	N	Mean / %	Obs	N	Mean / %	Obs	N	Mean / %	Obs	N	Mean / %
Age, years	286	-	31.2	287	-	31.0	290	-	25.1	278	-	24.3
Experience - months in sex work	289	-	56.6	290	-	54.9	289	-	38.8	278	-	36.0
**Nationality**												
Cameroonian (%)	289	288	100%	290	288	99%	290	290	100%	278	277	100%
**Education**												
Currently studying	289	41	14%	290	56	19%	290	129	44%	278	136	49%
**Highest achieved level of education**												
Primary	288	43	15%	290	41	14%	290	24	8%	278	19	7%
Secondary primary cycle	288	104	36%	290	86	30%	290	70	24%	278	70	25%
Secondary second cycle	288	82	28%	290	91	31%	290	112	39%	278	91	33%
Superior	288	55	19%	290	63	22%	290	80	28%	278	93	33%
No education	288	4	2%	290	1	3%	290	3	0%	278	2	1%
Other	288	0	0%	290	8	0%	290	1	1%	278	3	1%
**Marital status**												
Never married	289	258	89%	290	257	89%	290	282	97%	278	272	98%
Separated	289	20	7%	290	19	7%	290	5	2%	278	5	2%
Divorced	289	3	1%	290	7	2%	290	0	0%	278	0	0%
Widowed	289	8	3%	290	7	2%	290	3	1%	278	1	0%
**Economic Variables**												
Household size	289	-	4.3	290	-	4.4	290	-	5.7	278	-	5.6
Number of economic dependents	289	-	3.1	290	-	3.1	290	-	2.9	278	-	3.0
Number of child economic dependents	289	-	1.8	290	-	1.9	290	-	1.6	278	-	1.9
Have at least one child economic dependents	289	235	81%	290	254	88%	290	239	82%	278	241	87%
Number of biological children	289	-	2.1	290	-	1.9	290	-	1.2	278	-	1.2
Household expenditure per adult equivalent	289	-	54,804 CFA	290	-	47,426 CFA	290	-	32,933 CFA	278	-	38,776 CFA
**Sex work and behaviours**												
Number of clients/sugar daddies in typical week	268	-	8.5	265	-	8.2	290	-	2.8	278	-	2.6
Number of sex acts in a typical week	286	-	17.9	288	-	17.4	290	-	4.0	278	-	3.8
Average earnings from sex work	289	-	92,332 CFA	290	-	93,748 CFA	290	-	45,048 CFA	278	-	42,986 CFA
Used PrEP before	289	25	9%	290	19	7%	290	0	0%	278	1	0%
Had an HIV test in the last 12 months	288	267	93%	290	268	92%	290	167	58%	277	157	57%
Used condom at last sex - direct question	289	245	85%	290	249	86%	290	144	50%	278	140	50%
Used condom at last sex - list experiment	578	-	81%	580	-	80%	580	-	44%	556	-	46%

3) Please complete the CONSORT checklist and ensure that all components of CONSORT are present in the manuscript, including [how randomization was performed, allocation concealment, blinding of intervention, definition of lost to follow-up, power statement]. When completing the checklist, please use section and paragraph numbers, rather than page numbers.

This has been completed.

4) Please include the study protocol document and analysis plan, with any amendments, as Supporting Information to be published with the manuscript if accepted.

Study protocol is both referenced and included in this submission.

5) Please provide a flowchart (CONSORT flowchart may be used as a model) and indicate the number of individuals in each group analyzed in the ITT analysis.

A consort based flowchart has been added to the appendix, Figure A4

6) The trial protocol lists "STI acquisition measured using % test positive for syphilis, % test positive for chlamydia, % test positive for gonorrhoea" as one of the two primary outcomes but it is listed as a secondary outcome in the current manuscript. Please clarify and explain this discrepancy.

We have updated to paper to reflect STIs were specified as a primary outcome as well as HIV.

7) The trial protocol lists several secondary outcomes of, such as violence, stress as measured by the Perceived Stress Scale 4 (PSS-4), or loneliness, which are not reported here. You state that the current manuscript only examines the outcomes relating directly to health and risky behavior outcomes. Please list all secondary outcomes in the Methods section and please indicate those that will be reported elsewhere.

We have included the suggested outcomes in the reported results in Tables 4 and A8, plus write-up in the results and other places. We have also corrected and listed a summary of the secondary variables not included in the paper in the methods.

Please find them and their new interpretation copied below:

	Treatment			Control			Treatment			
Sexual Behaviour & Secondary Outcomes	obs	n	% / mean	obs	n	% / mean	obs	n	% / mean	obs
<u>**Commercial Sex**</u>										
Number of regular clients in the last 7 days	287	-	3.84	289	-	3.89	180	-	2.96	185
Number of occasional clients in the last 7 days	281	-	0.05	282	-	4.54	180	-	3.25	184
Number of clients in the last 7 days (combined)	279	-	8.85	281	-	8.46	180	-	6.21	184
Number of sex acts in the last 7 days	287	-	11.75	290	-	11.12	246	-	5.60	243
Number of vaginal sex acts over previous two clients‡	578	562	97.2%	580	567		360	356	98.9%	370
Number of oral sex acts over previous two clients‡	574	69	12.0%	580	93	16.0%	359	34	9.5%	370
Number of anal sex acts over previous two clients‡	577	12	2.1%	580	15	2.6%	360	5	1.4%	370
Self-reported riskiness of clients (average /10)‡	578	-	0.38	580	-	0.38	360	-	0.59	380
Used condom at last sex act - Direct questioning†	289	245	84.8%	290	249	85.9%	180	161	89.4%	184
Quit commercial sex work†	-	-	-	-	-	-	218	49	22.5%	221
Suffered violence from sexual partner	289	83	28.7%	290	124	42.8%	246	79	32.1%	243
Stress (PPS-4)	-	-	-	-	-	-	246	-	7.98	243
Loneliness (UCLA 3 point scale)	289	-	4.97	290	-	5.11	246	-	5.67	243
**List Experiment**										
Used condom at last sex act - List experiment	-	-	-	-	-	-	-	-	-	-
<u>**Transactional Sex**</u>										
Number of regular papy in the last 7 days	290	-	1.49	278	-	1.43	186	-	1.42	213
Number of occasional papy in the last 7 days	290	-	0.46	277	-	0.51	186	-	0.73	213
Number of papy in the last 7 days	290	-	1.95	278	-	1.94	263	-	1.52	267
Number of sex acts in the last 7 days	290	-	2.64	278	-	2.74	263	-	2.13	267
Number of vaginal sex acts‡	520	515	99.0%	593	476	80.3%	367	363	98.9%	412
Number of oral sex acts‡	520	68	13.1%	493	87	17.6%	366	47	12.8%	414
Number of anal sex acts‡	518	13	2.5%	494	11	2.2%	367	3	0.8%	414
Self-reported riskiness of clients (average /10)‡	520	-	0.47	495	-	0.48	358	-	0.35	400
Used condom at last sex act - Direct questioning†	290	144	49.7%	278	140	50.4%	186	82	44.1%	213
Quit transactional sex work†	-	-	-	-	-	-	247	63	25.5%	249
Suffered violence from sexual partner	290	35	12.1%	278	36	12.9%	263	32	12.2%	267
Stress (PPS-4)	-	-	-	-	-	-	263	-	8.05	267
Loneliness (UCLA 3 point scale)	290	-	5.82	287	-	5.60	263	-	5.35	267
**List Experiment**										
Used condom at last sex act - List experiment	-	-	-	-	-	-	-	-	-	-
<u>**Pooled (commercial + transactional)**</u>										
**Continuous outcomes - OLS**										
Number of regular clients/papy in the last 7 days	577	-	2.66	567	-	2.68	366	-	2.18	398
Number of occasional clients/papy in the last 7 days	571	-	2.7	559	-	2.55	366	-	2.33	397
Number of papy's or clients/papy in the last 7 days	579	-	5.32	568	-	5.18	509	-	3.24	510
Number of sex acts in the last 7 days	579	-	7.53	5.68	-	7.02	509	-	3.81	510
Number of vaginal sex acts‡	1098	1077	98.1%	1073	1043	97.2%	727	719	98.9%	782
Number of oral sex acts‡	1094	137	12.5%	1073	180	16.8%	725	81	11.2%	784
Number of anal sex acts‡	1095	25	2.3%	1074	26	2.4%	727	8	1.1%	784
Self-reported riskiness of clients/papy (average /10)‡	1098	-	0.42	1075	-	0.43	718	-	0.47	770
Used condom at last sex act - Direct questioning†	579	389	67.2%	568	389	68.5%	366	243	66.4%	397
Quit either commercial or transactional†	-	-		-	-	-	465	112	24.1%	470
Suffered violence from sexual partner	579	118	20.4%	568	160	28.2%	509	111	21.8%	510
Stress (PPS-4)	-	-	-	-	-	-	509	-	8.02	510
Loneliness (UCLA 3 point scale)	579	-	5.4	568	-	5.35	509	-	5.51	510
**List Experiment**										
Used condom at last sex act - List experiment	-	-	-	-	-	-	-	-	-	-

Significance levels: * p<0.1, ** p<0.05, *** p<0.01

Notes: Dependent binary variables (†) were estimated using logistic regression reporting marginal effects. All other continuous ou
effects. (log) indicate where the outcome is logged in the OLS model. Unadjusted = compares outcomes of follow-up data without
the outcome was collected during midline, then baseline controls, namely log of adult equivalent expenditure, number of economi
list experiment was estimated using OLS using the double list experiment method (Lépine et al., 2020) hence twice the observatio

307 ‡ = Data taken from the last two sex acts per sex worker.

Sexual Behaviour & Secondary Outcomes	Unadjusted odds n	OR	95% CI	Adjusted odds n	AOR	95% CI
**Commercial Sex**						
Used condom at last sex act - Direct questionning	364	1.151	0.600 , 2.207	364	1.148	0.589 , 2.238
Quit commercial sex work	439	1.045	0.666 , 1.640	439	1.055	0.655 , 1.701
Quit commercial sex work - bootstrapped	439	1.045	0.672 , 1.625	439	1.055	0.651 , 1.712
Suffered violence from sexual partner	489	0.982	0.670 , 1.435	489	1.014	0.689 , 1.491
**Trasnactional Sex**						
Used condom at last sex act - Direct questionning	399	1.099	0.738 , 1.635	399	1.069	0.712 , 1.604
Quit transactional sex	496	1.687**	1.089 , 2.615	496	1.682**	1.075 , 2.632
Quit transactional sex - bootstrapped	496	1.687**	1.090 , 2.614	496	1.682**	1.049 , 2.696
Suffered violence from sexual partner	530	1.470	0.835 , 2.586	530	1.493	0.841 , 2.651
**Pooled (commercial + transactional)**						
Used condom at last sex act - Direct questionning	763	1.149	0.853 , 1.548	763	1.189	0.869 , 1.627
Number exited either type of sex work	935	1.340*	0.980 , 1.832	935	1.308	0.948 , 1.804
Number exited either type of sex work - bootstrapped	935	1.340*	0.963 , 1.863	935	1.308*	0.957 , 1.787
Suffered violence from sexual partner	1,019	1.116	0.825 , 1.509	1,019	1.192	0.876 , 1.622

Significance levels: * p<0.1, ** p<0.05, *** p<0.01

Notes: All models estimated using logistic regression reporting odds ratios. Unadjusted = compares outcomes of follow-up data without adjustment. Adjusted = adjusts for baseline levels of the outcome, if the outcome was collected during midline, then baseline controls, namely log of adult equivalent expenditure, number of economic dependents, household size and if the respondent has a child.

[Results] 'Table 4 shows the impact of the intervention on risky sexual behaviours and other secondary outcomes. The intervention led to a significant increase in the likelihood of quitting transactional sex by 8.6 percentage points (16.9% vs 25.5%), or a 50% increase (ME = 0.086 (95% CI [0.015,0.158]); p=0.018) in likelihood of leaving sex work. There is an increase in condom use at the last sex act of around 15 percentage points, or a 43% increase, when measured using the list experiment method (ME=0.152 (95% CI[-0.021,0.324]); p=0.085) and we observe a decrease in the number of sex acts by 0.36 points on average for women in transactional sex (16% drop), which is statistically significant at 10% (ME=-0.358 (95% CI[-0.781,0.065]); p=0.097). For FSWs, health insurance had mixed results on risky sexual behaviours. The intervention was not effective in allowing FSWs to leave sex work and it did not increase condom use. However, it led to very small increases in the likelihood of engaging in anal sex (ME=0.014 (95% CI [0.002,0.026]); p=0.023) and on the riskiness of clients (ME=0.039 (95% CI [0.009,0.070]); p=0.012). The study found that the intervention had no impact on the likelihood of experiencing physical violence, as well as the degree of loneliness and stress levels among participants. Appendix **Error! Reference source not found.** in **Error! Reference source not found.** provide both unadjusted and adjusted odd ratios. '

8) Please present the safety data for the study including numbers of specific events and whether or not adverse events are thought to be related to the intervention.

In the ethics section, I have added the sentence:

"There were no adverse events during the study."

DISCUSSION

Please present and organize the Discussion as follows: a short, clear summary of the article's findings; what the study adds to existing research and where and why the results may differ from previous research; strengths and limitations of the study; implications and next steps for research, clinical practice, and/or public policy; one-paragraph conclusion.

We have changed the order of the discussion to fit this template. We have also added the following paragraph to the discussion:

[Discussion, paragraph 12] "Further research is necessary to gain a better understanding of the heterogeneous impacts between the two study populations. Additionally, a thorough estimation of the cost-effectiveness of the intervention to prevent HIV at the population level is important, taking into account the impact of the infections averted on other sexual partners. Furthermore, research is needed to determine the applicability of similar interventions for other common causes of economic shocks. For example, women in rural areas who are at risk of contracting HIV are often highly exposed to economic shocks caused by droughts which translates in risky sexual behaviours [18]. Therefore, drought insurance could be a suitable tool to prevent HIV in this context. Additionally, future research should focus on preventing HIV among FSWs. For this group, combining this intervention with an intervention that enables FSWs to substitute their earnings from sex work (e.g. cash transfer) might be more effective. "

FIGURES

For all Figures, please ensure that you have complied with our figures requirements [https://eur01.safelinks.protection.outlook.com/?url=http%3A%2F%2Fjournals.plos.org%2Fplosmedicine%2Fs%2Ffigures&data=05%7C02%7Ca.lepine%40ucl.ac.uk%7Cb16dd0b10a8441c7f42c08dc3867034c%7C1faf88fea9984c5b93c9210a11d9a5c2%7C0%7C0%7C638447262611016045%7CUnknown%7CTWFpbGZsb3d8eyJWIjoiMC4wLjAwMDAiLCJQIjoiV2luMzIiLCJBTiI6Ik1haWwiLCJXVCI6Mn0%3D%7C0%7C%7C%7C&sdata=xZtWGBFVZNpIcUFHyBWxx%2B012mIjf%2F7vbDrg3zSEW60%3D&reserved=0].

1) Please provide titles and legends for all figures (including those in Supporting Information files).

All figures and titles are included on figures and tables.

2) Please consider avoiding the use of red and green in order to make your figure more accessible to those with color blindness.

We have no instances of this in the paper.

3) Please in the figure legend/description, define abbreviations used in each figure (including those in Supporting Information files).

We have done it.

4) Please define the meaning of all dots, lines and bars in the captions/footnotes.

We adhere to these suggestions.

TABLES

1) Please provide titles and legends for all tables (including those in Supporting Information files).

All the points involving tables have been addressed in their relevant tables.

2) Please define all abbreviations used in the table below each table (including those in Supporting Information files).

All the points involving tables have been addressed in their relevant tables.

SUPPLEMENTARY MATERIAL

1) For supplementary figures and tables, please see the general comments under TABLES and FIGURES (color, abbreviations, titles, descriptions, etc.) and amend accordingly.

We have done this.

2) We suggest reporting statistical information as detailed above – see under ABSTRACT. Please be sure to define all numerical values.

We have done this.

3) As for the main manuscript, please indicate whether analyses are adjusted to help facilitate transparent data reporting please also detail the factors adjusted for and present the unadjusted analyses for comparison. If not, please clearly state the reasons why not.

All adjusted analyses are separated with details of what was adjusted with estimates labelled in tables as “adjusted”.

4) Please revise the references in the supplementary material according to comments detailed below – see under REFERENCES.

We have done this.

5) Please cite your Supporting Information as outlined here: https://eur01.safelinks.protection.outlook.com/?url=https%3A%2F%2Fjournals.plos.org%2Fplosmedicine%2Fs%2Fsupporting-information&data=05%7C02%7Ca.lepine%40ucl.ac.uk%7Cb16dd0b10a8441c7f42c08dc3867034c%7C1faf88fea9984c5b93c9210a11d9a5c2%7C0%7C0%7C638447262611020828%7CUnknown%7CTWFpbGZsb3d8eyJWIjoiMC4wLjAwMDAiLCJQIjoiV2luMzIiLCJBTiI6Ik1haWwiLCJXVCI6Mn0%3D%7C0%7C%7C%7C&sdata=udWwC47knDg1ug7kVSY6SqPqyVsoqixcgWjZEw8dztI%3D&reserved=0

We have done this.

REFERENCES

1) PLOS uses the numbered citation (citation-sequence) method and first six authors, et al.

References are now formatted in this way.

2) Please ensure that journal name abbreviations match those found in the National Center for Biotechnology Information (NCBI) databases (https://eur01.safelinks.protection.outlook.com/?url=http%3A%2F%2Fwww.ncbi.nlm.nih.gov%2Fnlmcatalog%2Fjournals&data=05%7C02%7Ca.lepine%40ucl.ac.uk%7Cb16dd0b10a8441c7f42c08dc3867034c%7C1faf88fea9984c5b93c9210a11d9a5c2%7C0%7C0%7C638447262611026008%7CUnknown%7CTWFpbGZsb3d8eyJWIjoiMC4wLjAwMDAiLCJQIjoiV2luMzIiLCJBTiI6

Ik1haWwiLCJXVCI6Mn0%3D%7C0%7C%7C%7C&sdata=IosUSrqXo8OISiiBrTi2yTDrmYOnBWhridf7w9Oa1ig%3D&reserved=0), and are appropriately formatted and capitalised.

All journal abbreviations should adhere to these requirements

3) Where website addresses are cited, please specify the date of access (e.g. [accessed: 12/06/2023]).

This has been updated.

4) Please also see https://eur01.safelinks.protection.outlook.com/?url=https%3A%2F%2Fjournals.plos.org%2Fplosmedicine%2Fs%2Fsubmission-guidelines%23loc-references&data=05%7C02%7Ca.lepine%40ucl.ac.uk%7Cb16dd0b10a8441c7f42c08dc3867034c%7C1faf88fea9984c5b93c9210a11d9a5c2%7C0%7C0%7C638447262611030914%7CUnknown%7CTWFpbGZsb3d8eyJWIjoiMC4wLjAwMDAiLCJQIjoiV2luMzIiLCJBTiI6Ik1haWwiLCJXVCI6Mn0%3D%7C0%7C%7C%7C&sdata=sjW3CY9bK6gVAohw%2Ff9kgDYmR9KvPF4FrhDHHCNRJvo%3D&reserved=0 for further details on reference formatting.

5) RE reference [24] listed as "Working paper", papers cannot be listed in the reference list until they have been accepted for publication or are publicly available on a preprint archive.

This reference has been updated.

General editorial requests:

1) Please ensure that the paper adheres to the PLOS Data Availability Policy (see https://eur01.safelinks.protection.outlook.com/?url=http%3A%2F%2Fjournals.plos.org%2Fplosmedicine%2Fs%2Fdata-availability&data=05%7C02%7Ca.lepine%40ucl.ac.uk%7Cb16dd0b10a8441c7f42c08dc3867034c%7C1faf88fea9984c5b93c9210a11d9a5c2%7C0%7C0%7C638447262611065291%7CUnknown%7CTWFpbGZsb3d8eyJWIjoiMC4wLjAwMDAiLCJQIjoiV2luMzIiLCJBTiI6Ik1haWwiLCJXVCI6Mn0%3D%7C0%7C%7C%7C&sdata=jLAC47FC3xioiYAl%2B493hfx06%2BJRZe69lnpJUEFBev0%3D&reserved=0), which requires that all data underlying the study's findings be provided in a repository or as Supporting Information. For data residing with a third party, authors are required to provide instructions with contact information for obtaining the data. PLOS journals do not allow statements supported by "data not shown" or "unpublished results." For such statements, authors must provide supporting data or cite public sources that include it.

The data is available on a public repository at UCL at:
https://rdr.ucl.ac.uk/articles/dataset/POWER_RCT_dataset/25244878/1

2) In addition, we request that you upload any figures associated with your paper as individual TIF or EPS files with 300dpi resolution at resubmission; please read our figure guidelines for more information on our requirements: https://eur01.safelinks.protection.outlook.com/?url=http%3A%2F%2Fjournals.plos.org%2Fplosmedicine%2Fs%2Ffigures&data=05%7C02%7Ca.lepine%40ucl.ac.uk%7Cb16dd0b10a8441c7f42c08dc3867034c%7C1faf88fea9984c5b93c9210a11d9a5c2%7C0%7C0%7C638447262611070445%7CUnknown%7CTWFpbGZsb3d8eyJWIjoiMC4wLjAwMDAiLCJQIjoiV2luMzIiLCJBTiI6Ik1haWwiLCJXVCI6Mn0%3D%7C0%7C%7C%7C&sdata=hAxtvweGmncFurdqeGbej%2FBelaI4Dl1O0ozE32pdejg%3D&reserved=0. While revising your submission, please upload your figure files to the PACE digital diagnostic tool, https://eur01.safelinks.protection.outlook.com/?url=https%3A%2F%2Fpacev2.apexcovantage.com%2F&data=05%7C02%7Ca.lepine%40ucl.ac.uk%7Cb16dd0b10a8441c7f42c08dc3867034c%7C1faf88fea9984c5b93c9210a11d9a5c2%7C0%7C0%7C638447262611075245%7CUnknown%7CTWFpbGZsb3d8eyJWIjoiMC4wLjAwMDAiLCJQIjoiV2luMzIiLCJBTiI6Ik1haWwiLCJXVCI6Mn0%3D%7C0%7C%7C%7C&sdata=8Yn490vdOVwL%2FWB5e2CZUXXmOa7j700ACEZn9kA%2FXbk%3D&reserved=0. PACE helps ensure that figures meet PLOS requirements. To use PACE, you must first register as a user. Then, login and navigate to the UPLOAD tab, where you will find detailed instructions on how to use the tool. If you encounter any issues or have any questions when using PACE, please email us at PLOSMedicine@plos.org.

All figures meet these requirements.

3) We ask every co-author listed on the manuscript to fill in a contributing author statement, making sure to declare all competing interests. If any of the co-authors have not filled in the statement, we will remind them to do so when the paper is revised. If all statements are not completed in a timely fashion this could hold up the re-review process. If new competing interests are declared later in the revision process, this may also hold up the submission. Should there be a problem getting one of your co-authors to fill in a statement we will be in contact. YOU MUST NOT ADD OR REMOVE AUTHORS UNLESS YOU HAVE ALERTED THE EDITOR HANDLING THE MANUSCRIPT TO THE CHANGE AND THEY SPECIFICALLY HAVE AGREED TO IT. You can see our competing interests policy here: https://eur01.safelinks.protection.outlook.com/?url=http%3A%2F%2Fjournals.plos.org%2Fplosmedicine%2Fs%2Fcompeting-interests&data=05%7C02%7Ca.lepine%40ucl.ac.uk%7Cb16dd0b10a8441c7f42c08dc3867034c%7C1faf88fea9984c5b93c9210a11d9a5c2%7C0%7C0%7C638447262611079999%7CUnknown%7CTWFpbGZsb3d8eyJWIjoiMC4wLjAwMDAiLCJQIjoiV2luMzIiLCJBTiI6Ik1haWwiLCJXVCI6Mn0%3D%7C0%7C%7C%7C&sdata=NvNWRDqxPtHKP4sAjs6oJzETlbg%2FU%2Fx9gn7Fo0EB3m8%3D&reserved=0.

We are unsure whether co-authors have sent their statement.

In compliance with data protection regulations, you may request that we remove your personal registration details at any time. (Use the following URL: https://eur01.safelinks.protection.outlook.com/?url=https%3A%2F%2Fwww.editorialmanager.com%2Fpmedicine%2Flogin.asp%3Fa%3Dr&data=05%7C02%7Ca.lepine%40ucl.ac.uk%7Cb16dd0b10a8441c7f42c08dc3867034c%7C1faf88fea9984c5b93c9210a11d9a5c2%7C0%7C0%7C638447262611085143%7CUnknown%7CTWFpbGZsb3d8eyJWIjoiMC4wLjAwMDAiLCJQIjoiV2luMzIiLCJBTiI6Ik1haWwiLCJXVCI6Mn0%3D%7C0%7C%7C%7C&sdata=BLI27Gj%2FYM2fMZfI50o3LkB9UxFozgumdUxDJvqhq%2Fc%3D&reserved=0). Please contact the publication office if you have any questions.
