# Supplementary material for: The effect of protecting women against economic shocks to fight HIV in Cameroon, Africa: The POWER randomised controlled trial
Source: PLoS Med. 2024 Oct 24;21(10):e1004355. doi: 10.1371/journal.pmed.1004355 (PMC11500901; doi:10.1371/journal.pmed.1004355)
Supplement: Attachment — Submitted filename: Review report_PMEDICINE-D-24-00181R1.docx [file pmed.1004355.s007.docx]

Review report for PMEDICINE-D-24-00181R1

Thanks for inviting me to review this article.

Addressing gender inequalities is a significant challenge in tackling HIV, both in sub-Saharan Africa and globally. This article is essential and timely in this regard. A trial on formal economic mechanisms to cope with shocks to avert HIV risks is much needed and novel. The researchers provide a detailed and convincing data set to justify the inclusion of shock-coping strategies as policy tools for addressing HIV in sub-Saharan Africa.

Overall, this is an exciting article.

1) However, the eligibility criteria considered engagement in either transactional or commercial sex (see pg. 5), which seems to influence the results – showing positive outcomes in one category and none in the other. The reader may wonder how this study defined/differentiated the two in an African context (Yaunde).
2) Following the above concern, it is important to state – where such definitions come from to avoid imposition and epistemic injustices that have historically characterised global health practice.
3) It is interesting that the intervention allowed women to leave transactional sex and an increase in condom use and reduction in sex acts for those who remained in TS (pg. 15). However, these results are based on self-reports. Thus, they cannot form a solid basis for policy recommendations. Adding caution as part of the study's limitation can help clear some of these concerns.

Generally, this study provides critical lessons for global health and local health experts in addressing HIV among women engaging in sex work and transactional sex.
