# Supplementary material for: The effect of protecting women against economic shocks to fight HIV in Cameroon, Africa: The POWER randomised controlled trial
Source: PLoS Med. 2024 Oct 24;21(10):e1004355. doi: 10.1371/journal.pmed.1004355 (PMC11500901; doi:10.1371/journal.pmed.1004355)
Supplement: Attachment — Submitted filename: response letter.docx [file pmed.1004355.s009.docx]

GENERAL COMMMENTS

Please check the grammar, spelling, and use of tenses throughout the main manuscript and revise the main text for clarity.

We have done this.

When referring to tables and figures in the main manuscript, please do not write "see Table A3", but add parentheses referring to the table at the end of a sentence (e.g. (Table A3)).

We have made these changes.

FINANCIAL DISCLOUSRE

Please update the Financial Disclosure Statement in the online submission form using the information provided under "Funding," lines 508-510.

We have added this.

ABSTRACT

1) Please include the age of the participants

We now make clear that we recruited participants over 15 years old.

2) Please include the recruitment dates under Methods and Findings.

We have added this.

3) l.13: We suggest changing the word ‘falls’ to ‘decreases’.

We have changed this.

4) l.15: Please change to “We investigated...”

We have changed this.

5) ll.20-22: Please change to: ““Participants were randomly assigned (1:1) to receive free health insurance for themselves and their economic dependents for 12 months either at the beginning of the study (intervention; n=XX) or at the end of the study (control; n=XX).”

We have changed this.

6) ll.22-25: Please clearly specify the primary outcome of the trial and timing of assessment and indicate the planned population for the primary outcome.

We have specified these.

7) ll.25-27: Please specify the time point of this finding (Was this at endline?).

We have made clear that we use data from "midline or endline".

8) l.26: We suggest exchanging “treatment” with “intervention”.

We have made this change.

9) ll.28-29: “There was also no effect on STI acquisition.” – Please clarify whether this applies for both strata.

We now specified it does apply to both strata.

10) l.30: Please write FSW in full

We have done this.

11) l.31: Please temper claims of primacy of results by stating, "to our knowledge" or something similar (“The study provides, to our knowledge, the first evidence of the effectiveness..”)

We have changed this.

AUTHOR SUMMARY

1) For each section, please use the questions only as subheadings (i.e. ‘Why Was This Study Done?’, ‘What Did the Researchers Do and Find?’, ‘What Do These Findings Mean?’).

We have changed this.

2) l.57: We suggest replacing "treatment" with "intervention".

We have changed this.

3) ll.61-63: Under ‘What Did the Researchers Do and Find?’, we suggest changing the last bullet point to: “The effect of the intervention on primary outcomes was large among women engaging in transactional sex, but there was no effect of the intervention in the commercial sex strata.”

We have changed this.

4) ll.73-74: Under ‘What Do These Findings Mean?’, we suggest changing the last bullet point to: “The results also highlight that women in transactional sex are more affected by HIV than sex workers, a finding that warrants attention from policy makers.”

We have changed this.

5) In the final bullet point of ‘What Do These Findings Mean?’, please describe the main limitations of the study in non-technical language.

We have added this: “the main limitation of this study was the poor sensitivity and specificity of STI tests.”

INTRODUCTION

1) ll.98-99: Please note that there is a single upper quotation mark (“defined as ‘an unexpected”). We suggest removing the quote, the reference is sufficient.

We have changed this.

2) ll.100-102: We suggest changing this to: “Within such relationships, women are incentivised to provide unprotected sex to men [14] because they can earn more, for example between 9% more in Kenya and 66% more in India for unprotected sex [15–17].”

We have changed this.

3) l.104: Please change to: “…to provide basic needs [13,18].”

We have changed this.

4) l.114: Please define “STI” at first use.

We have done this.

METHODS AND RESULTS

1) l.143: Please also mention that the trial protocol is available as supporting information and include the appropriate reference.

We have added this "The protocol paper with more details on the methods can be found in the supporting information [30]".

2) l.156: Please define “FSW” at first use.

We have done this.

3) l.160: Please define or briefly explain the term “sugar daddies”.

We have added this: " ... [31] and typical have boyfriends or “sugar daddies” in this context which we define as a man who gives money, gifts, etc., to a younger woman in exchange for sex"

4) l.175: Please change “23 transactional and 31 commercial women” to “23 women in the transactional strata and 31 in the commercial strata” or similar**.**

We have changed this.

5) l.181: Please define “ART” at first use.

We have done this.

6) l.204: We suggest exchanging “treatment” with “intervention”. Please revise throughout the main manuscript including tables and figures (including those in the Supplementary Material).

We have done this.

7) Table 1: Please define “Obs” and “PrEP”. Please add a definition for “sugar daddies”.

We have done these.

8) l.233: Please remove the footnote and include the information in the text.

We have done this.

9) l.238: Please define “ID” at first use

We have done this.

10) ll.276.277: Please change to: “….and we collected data on unprotected sex using the double-list experiment method (See appendix 7.2 for details).”

We have done this.

11) ll.296-310: Please revise the two paragraphs for grammar and tense

We have updated the tenses throughout.

11) ll.313-323: Please use the past tense when describing the statistical analysis.

We have done this.

12) l.333 ff: Please use the past tense when writing your results (see GENERAL comments).

We have done this.

13) ll.340-342: Please change to: “The absence of effect could be due to serious problems found with the STI tests because they were not effective in determining STI status with certainty.”

We have changed this.

14) ll.343-344: Please change to: “At endline, as many samples as possible were retested in the ELISA laboratory using new types of assays.”

We have changed this.

15) l.355: Please define “ME” at first use.

We have done this.

16) Table 4: Please report exact p-values. Please define “OLS” and “Obs”. In the table description, please briefly explain what PPS-4 and UCLA 3 point scale are and/or provide references.

We have done this.

18) l.381: Please remove any footnotes and include the information provided in the footnotes in the main text, if desired.

We have done this.

19) Table 5: Please report exact p-values. Please define “OLS”, “ME”, and “CI”

Note that the statistician reviewer asked us to report CI only, the reason why we removed p-values, should we add them again? We have changed the abbreviations, however.

DISCUSSION

1) ll.407-408: Please change to: “Our results show the importance of including transactional sex in HIV transmission research, as we found 9 times more infections among women in transactional sex than among women in commercial sex in the control group.”

We have changed this.

2) ll.441-444: Please do not report exact values in the Discussion, as this should be limited to the Results section (please revise throughout the Discussion). Please change to: "This may explain why there was no increase in the likelihood of visiting a professional among FSWs compared to women who engage in transactional sex."

We have changed this.

3) l.467 and l.500: Please temper claims of primacy of results by stating, "to our knowledge" or something similar.

We have changed this.

FIGURES

1) Figure 1: Please define “STI”. We also suggest that the figure description briefly explain what the figure is supposed to show (as described in the main text)

We have changed this.

2) Figure 2: In the figure description, please describe the meaning of the bars and whiskers. Please describe the meaning of the green percentages in the figure, and we suggest adding the n numbers for the different groups to the description.

We have done this.

SUPPLEMENTARY MATERIAL

1) You have indicated that you have completed the CONSORT checklist. It appears that the completed CONSORT checklist was not uploaded as a supporting information file. When completing the checklist, please remember to use section and paragraph numbers, not page numbers

We have now attached the SPIRIT checklist, which is to our mind an equivalent to the CONSORT checklist.

2) We were unable to access the file "data_plos med.dta". Could you please provide the contents of the file in an alternative way?

We are now providing this file in the excel format.

3) Figure A1: Please include the CONSORT flowchart in the main manuscript. **–**

We have added it.

4) Figure A2: Please define “STI”.

We have defined the term.

5) Figure A3: Please present numerators and denominators for percentages. –

We have provided the numbers in the Figure notes.

6) Table A1-A10: Please see the comments for the main tables and revise accordingly. Please report exact p-values and ensure to define all abbreviations (e.g. “Obs”, “ME”, “STI”,”OR”, etc.)

We have changed this.

7) Table A9: What do Magenta and Teal refer to?

It was how we blinded groups and has been corrected.

8) Table A10: Please remove the asterisks given you are reporting the exact p-

We did it.

REFERENCES

1) Please revise your references. Some references, e.g. [34] and [41], appear to be under review and cannot be listed in the reference list until they have been accepted for publication or are publicly available on a preprint archive. The information may be cited in the text as a personal communication with the author if the author provides written permission to be named. Alternatively, please provide a different appropriate reference. -

Both these papers have now been uploaded to UCL discovery working paper repository and are referring to these in the references.

3) Please revise references [4] and [7] as they do not appear to provide sufficient detail. Please check if this applies to any other references.

We have added additional detail to these references.

4) Where website addresses are cited, when specifying the date of access, please use the word “accessed” instead of “cited” (e.g. [accessed: 12/06/2023]).

We have changed this.

SOCIAL MEDIA

To help us extend the reach of your research, please provide any X (formerly known as Twitter) handle(s) that would be appropriate to tag, including your own, your co-authors’, your institution, funder, or lab. Please enter in the submission form any handles you wish to be included when we post about this paper –

Please add these:

@aure_lepine

@henrycust

@SandSzawlowski

@UCLGlobalHealth

@POWER_UCL

[@UKRI_News](https://twitter.com/UKRI_News)
