# Supplementary material for: The effect of protecting women against economic shocks to fight HIV in Cameroon, Africa: The POWER randomised controlled trial
Source: PLoS Med. 2024 Oct 24;21(10):e1004355. doi: 10.1371/journal.pmed.1004355 (PMC11500901; doi:10.1371/journal.pmed.1004355)
Supplement: Attachment — Submitted filename: Comments from Editor_July.docx [file pmed.1004355.s010.docx]

Requests from Editors:

GENERAL COMMENTS

Please remove the terms/descriptions "midline" and "endline" throughout the paper and refer to these time points as 6 months and 12 months post-intervention (including in display items such as figures and tables).

This change has been made throughout

FINANCIAL DISCLOSURE

Please revise the statement once more. The funding statement should include: specific grant numbers, initials of authors who received each award, URLs to sponsors’ websites. Also, please state whether any sponsors or funders (other than the named authors) played any role in study design, data collection and analysis, the decision to publish, or preparation of the manuscript. If they had no role in the research, include this sentence: “The funders had no role in study design, data collection and analysis, decision to publish, or preparation of the manuscript.”

This change has been made throughout

ABSTRACT

1) l.18: Please remove the term “high-risk women” and use patient-centered language instead, i.e., “women at high risk of HIV infection”.
This change has been made throughout

2) ll.19-20: Please check the numbers presented (1506 versus 1508), and revise throughout the main text. Please change to “Between June and August 2021, we recruited 1,508 adolescent girls and women over age 15 years who were involved in transactional sex (n=753) or commercial sex (n=755), using snowball sampling.”

This change has been made throughout

3) ll.23-26, please change to: We collected data on socioeconomic characteristics of participants. Primary outcomes included incidence of HIV and sexually transmitted infections (STIs). Outcomes were measured at baseline, 6 months after randomisation, and 12 months after randomisation.

This change has been made throughout

4) Please note that only the primary outcome of the trial should be reported in your Abstract. Our policy is that secondary outcomes should only be included in the Abstract if all secondary outcomes are included. For trials that have many secondary outcomes, such as yours, the abstract should be limited to reporting the primary outcome.

This change has been made in the abstract

5) l.30: Please change “the commercial sex strata” to “women and girls involved in commercial sex” (or similar). Please revise accordingly throughout the main text.

This change has been made throughout.

6) l.31: Please remove the term ‘biomarkers’. Editorial suggestion: The main limitations of this study were the challenges of collecting reliable STI incidence data and the low incidence of HIV in women and girls engaged in commercial sex, which might have prevented detection of study effects.

This change has been made.

7) Please include the details provided in lines 53-55 in the Abstract. Editorial suggestion: Participants were randomly assigned (1:1) to receive free health insurance for themselves and their economic dependents for 12 months either at the beginning of the study (intervention; n=579; commercial sex n = 289, transactional sex n = 290) from November 2021 or at the end of the study 12 months later (control; n=568; commercial sex n = 290, transactional sex n = 278).

This change has been made.

AUTHOR SUMMARY

1) ll.51-55: Please combine the second and third bullet point under ‘What Did the Researchers Do and Find?’.

This change has been made.

2) ll.58-59, please change to: Among women who received the intervention, we found large reductions in HIV infection and reductions in risky sexual behaviors for women and girls engaging in transactional sex, but there was no effect in women and girls engaging in commercial sex.

This change has been made.

3) Under ‘What Do These Findings Mean?’, please swap the last two bullet points as the main limitation(s) should be the last bullet point.

This change has been made.

INTRODUCTION

1) l.71, please change to: “Women aged 15-24 years in Sub-Saharan Africa…”
This change has been made.

2) l.75, please change to: “…adolescent girls aged 15–19 years..”

This change has been made.

3) ll.78-80: Please remove the quotes. Editorial suggestion: There is growing evidence that heterosexual sex as part of commercial and transactional relationships (i.e., non-commercial sexual relationships in exchange for material support and benefits) are a key driver of the HIV epidemic in sub-Saharan Africa.

This change has been made.

4) ll.86-89: Please provide references.

These have been provided.

5) ll.106-107: Please rephrase to “Use of cash transfers is one type of structural intervention used to address the underlying causes of HIV risk by improving income levels.”
This change has been made.

6) l.115: Please rephrase to “…have shown some success, including lottery-based financial incentives…”

This change has been made.

METHODS AND RESULTS

1) l.139: Please provide an appropriate reference for trial protocol as supporting information, i.e. “The protocol paper with more details on the methods can be found in the supporting information (S2 Study protocol) [30].”

We have not made this change and left the phrasing as: “Further information can be found in the study protocol in the supporting information” since we do not know the reference number the supporting information will have on submission.

2) l.140: Please remove the sentence. The flow diagram may be referenced later (see comment l.334).

This change has been made.

3) Figure 1: In flow diagram, for the boxes showing the follow-up section, please first show the total numbers of participants at follow-up with the numbers lost-to-follow up listed below.

This change has been made.

4) ll.146-153: Please note that there are two identical paragraphs. Please remove one.

This change has been made.

5) l.162: Please change to “…which we defined…”.
This change has been made.

6) l.195: Please also reference the Supporting Information file.
This change has been made.

7) l.200: Please reference figures/tables in parentheses, i.e. “…two months after the baseline survey had taken place in order to minimise attrition (Figure A1).”. Please change the label of the supplementary figure from ‘Figure A1’ to ‘S1 Figure’ (for further details see comment under ‘Supplementary Material’). Please revise throughout the main text.

This changed to “(S1 Figure in ‘Supplementary Material’)”.

8) l.223: Please remove the sentence “Figure 1 displays the flowchart of respondents in the analysis.”. It is sufficient to mention the flowchart once, which we suggest to do at the beginning of the Results section.

This change has been made.

9) ll.223-225, please change to: By completing randomisation after baseline only for returning participants, we reduced the attrition rate but may have reduced the generalisability of our results.

This change has been made.

10) Table 1: Please re-name to “Summary characteristics at baseline”.

This change has been made.

11) l.246: Please define ‘ID’ at first use.

This change has been made.

12) ll.253-255, please change to: “The primary outcome measure was HIV incidence, measured by rapid serological blood tests, with positive and indeterminate tests retested by enzyme-linked immunosorbent assay (ELISA), and STI incidence, including chlamydia, gonorrhoea, trichomoniasis and syphilis.”
This change has been made.

13) l.261: Please change to ‘…per protocol..’.

This change has been made.

14) ll.264-267: We feel this part is currently rather unclear. Please clarify the meaning of ‘intermediate’ – do you mean secondary outcomes? What does ‘intensity of commercial or transactional sex’ mean? Please list the prespecified secondary outcomes in clearly and in explicit detail and ensure that all prespecified outcomes are listed.

This change has been made.

15) l.271, please change to: “We hypothesized…”

This change has been made.

16) l.273, please change to: ”…lead to an increase in STIs treated,..”

This change has been made.

17) l.277: Please write ‘OOPs’ in full.

This change has been made.

18) l.293: Please remove the abbreviation MDE, as it is only used one additional time; you should then spell out “minimal detectable effect” at line 297.
This change has been made.

19) l.296: Please change ‘sub-samples’ to ‘sub-groups’.

This change has been made.

20) l.313: The term “attritors” to describe individuals lost to follow-up is not standard. Please revise to something more conventional.

This now reads “lost to follow up”

21) Figure 2: Please write ‘STI’ in full in the figure or add a definition in the figure description. Please add a brief explanation of what the figure depicts.

This change has been made

22) l.332, please change to: “…whether the respondent had a child.”

This change has been made

23) l.334: At the beginning of the Results section, please describe briefly the recruitment and enrollment schedule (i.e. participant numbers) of the trial. Please reference the flow diagram here.

This change has been made

24) l.337: Please reference figures/tables in parentheses.

This change has been made

25) l.342: Please define ‘CI’ and ‘AOR’ at first use.

This change has been made

26) l.343: Please rephrase “and when the samples were pooled” to avoid referring to participants as samples. Editorial suggestion: “…and in both strata combined”

This change has been made

27) Figure 3: Please change the x-axis title to ‘Intervention status’ and replace the word ‘treatment’ with ‘intervention’ in the whole figure. The y-axis is labeled ‘HIV prevalence’ – do you mean HIV incidence? Also, we suggest changing the y-axis to showing percentages. In the figure description, please add details about the number of participants included in each group, the meaning of the bars and whiskers and a brief explanation of what the figures shows (e.g. the time point measured, what the intervention entails etc.). We also suggest re-naming the first two graphs ‘Commercial Sex’ and ‘Transactional Sex’. Please remember that all figures and/or tables should be self-explanatory on a stand-alone basis. Please revise accordingly and provide sufficient detail, including the title.

This change has been made

28) ll.364ff: Causal language - In trials, there is usually a distinction in the language in terms of causal vs associational for primary and secondary trial outcomes. Please use associational language for secondary outcomes.

We feel that the strength of the association is equally valid regardless of the assignment of an outcome as primary or secondary. Our theory of change theoretically supports the causal link between the intervention and the secondary outcomes.

29) ll.366ff: Please remove any descriptions of percentage increases/decreases (50% increase, 43% increase, 16% reduction).

This change has been made.

30) ll.368-372: Please break down the sentence into multiple sentences to improve readability. "which was statistically significant at 10%" - please refrain from describing results as significant if the analysis was not pre-specified in the SAP. Editorial suggestion: There was an increase in condom use at last sex of about 15 percentage points as measured by the list experiment method (ME=0.152 (95% CI [-0.021,0.324]); p=0.085). We also observed a reduction in the number of sex acts by an average of 0.36 for women having transactional sex, which was not statistically significant (ME=-0.358 (95% CI [-0.781,0.065]); p=0.097).

This change has been made and reads as:

“There was an increase in condom use at last sex of about 15 percentage points as measured by the list experiment method that is not statistically significant at the 5% level (ME=0.152 (95% CI [-0.021,0.324]); p=0.085). We also observed a reduction in the number of sex acts by an average of 0.36 for women having transactional sex (ME=-0.358 (95% CI[-0.781,0.065]); p=0.097) that is also not statistically significant at 5% level.”

31) l.373: We have noticed that you switch between the descriptions "women engaging in commercial sex" and "FSW". For consistency, please use the description “women and girls engaged in commercial sex” throughout the main text. Please revise accordingly.
This change has been made.

34) Table 4: Since you provided the exact p-values in the table, please remove the asterisks from and below the table. Please define or replace the word "papy" (it does not appear that you have used the word elsewhere). In the table description, please add details about the midline and endline time points.

This change has been made

35) ll.384ff: Please use the past tense when describing methods and results.

The manuscript has been revised to fix this. Please highlight any area’s where this is not completed.

36) Table 5: Since you have reported the exact p-values in the table, please remove the asterisks in and below the table. In the table description, please add details about the midline and endline time points.

This change has been made but previous comments in earliest versions required we have asterisks.

DISCUSSION

1) ll.440-442, please change to: Women and girls engaged in commercial sex are considered a key population by UNAIDS [2] and have access to PrEP in Cameroon; among women and girls engaged in commercial sex in this study, 13% had ever taken PrEP at endline, while none of the women engaging in transactional sex were using PrEP at endline.

This change has been made.

2) ll.444ff: Please remove references to tables/figures (e.g. Table 1) as you do not need to re-reference the results in the Discussion section. If you feel a specific reference to a table or appendix is necessary, please use parentheses.

This change has been made.

3) l.471: Please avoid referring to the participants in your study as "sample" and/or "used". Please revise the main text accordingly. Editorial suggestion: The participants included in our study may be subject to bias due to the limitations of the snowball method.

This change has been made.

SUPPLEMENTARY MATERIAL

1) Thank you for providing the SPIRIT checklist. We noticed that you did not complete the checklist by using section and paragraph numbers to indicate where the item can be found in the manuscript. Please do so by adding a "Location Reported" column. Also, in the Methods section, please add the following statement: "This study is reported as per the Standard Protocol Items: Recommendations for Interventional Trials (SPIRIT) guideline (S1 Checklist)."

We submitted this checklist by mistake and is not appropriate since it is a checklist for the protocol. Please find the correct checklist (CONSORT 2010) as was submitted in the original submission.

2) Tables in the appendices: Please remove the asterisks from the tables and below the tables if you are reporting exact p-values.

This change has been made.

3) Figure A2: Please write “STI” in full.

4) Where website addresses are cited, when specifying the date of access, please use the word “accessed” instead of “cited” (e.g. [accessed: 12/06/2023]).
In the published article, supporting information files are accessed only through a hyperlink attached to the captions. For this reason, you must list captions at the end of your manuscript file. You may include a caption within the supporting information file itself, as long as that caption is also provided in the manuscript file. Do not submit a separate caption file.

This change has been made.
