# Supplementary material for: The effect of protecting women against economic shocks to fight HIV in Cameroon, Africa: The POWER randomised controlled trial
Source: PLoS Med. 2024 Oct 24;21(10):e1004355. doi: 10.1371/journal.pmed.1004355 (PMC11500901; doi:10.1371/journal.pmed.1004355)
Supplement: Attachment — Submitted filename: Comments from Editor_August.docx [file pmed.1004355.s011.docx]

Thanks for your comments and picking up where changes had slipped through the net. All changes have been made with exception of the last comment. See details below.

1) Title: We suggest changing the title to “The effect of protecting women against economic shocks to fight HIV in Cameroon, Africa: the POWER randomized controlled trial”.

This has been changed

2) ll.20-22, please change to: Between June and August 2021, we recruited 1,508 adolescent girls and women over age 15 years who were involved in transactional sex (n=753) or commercial sex (n=755), using snowball sampling.
This has been changed

3) ll.22-25, please change to: Participants were randomly assigned (1:1) to receive free health insurance for themselves and their economic dependents for 12 months either at the beginning of the study (intervention; n=579; commercial sex n = 289, transactional sex n = 290) from November 2021 or at the end of the study 12 months later (control; n=568; commercial sex n = 290, transactional sex n = 278).
This has been changed

4) ll.26-28, please change to: Primary outcomes included incidence of HIV and sexually transmitted infections (STIs) and were measured at baseline, 6 months after randomisation, and 12 months after randomisation.
This has been changed

5) ll.29-31, please remove the following sentence (Per CONSORT, only the primary outcome of the trial should be reported in the Abstract): “Secondary health and sexual behaviour outcomes at baseline, 6 months after randomisation (6 months post-intervention) and 12 months after randomisation (12 months post-intervention).”
This has been changed

6) ll.31-34, please change to: We found that study participants who engaged in transactional sex and were assigned to the intervention group were less likely to become infected with HIV post-intervention (combined result of 6 months post-intervention or 12 months post-intervention, depending on the follow-up data available; odds ratio (OR)=0.109 (95% confidence interval (CI)[0.014,0.870]); p=0.036).”
This has been changed

7) Abstract ll.37-39, please change to: The main limitations of this study were the challenges of collecting reliable STI incidence data and the low incidence of HIV in women and girls involved in commercial sex, which might have prevented detection of study effects.
This has been changed

8) Author Summary: In the last bullet point, please spell out ‘STI’ (sexually transmitted infection).
This has been changed

9) ll.90-93, please change to: There is growing evidence that heterosexual sex as part of commercial and transactional relationships (i.e., non-commercial sexual relationships in exchange for material support and benefits) are a key driver of the HIV epidemic in Sub-Saharan Africa.
This has been changed

10) Figure 2: Please write ‘STI’ in full in the figure or add a definition in the figure description.
This has been changed

11) Figure 3: For all three graphs, please:
a) change the x-axis title to ‘Intervention status’ and replace the word ‘treatment’ with ‘intervention’ below the second bar;
b) change the y-axis label to ‘HIV incidence’;
c) re-name the first two graphs ‘Commercial Sex’ and ‘Transactional Sex’
d) re-name the figure ‘Effect of free health insurance on HIV incidence’
This has been changed

12) Table 4: Please remove the description of the significance levels below the table.
This has been changed

13) Table 5: Please remove the terms ‘midline’ and ‘endline’ and write ‘6 months post intervention’ and ’12 months post intervention’ only.
This has been changed

14) Thank you for providing your CONSORT checklist. Please replace the page numbers with paragraph numbers per section (e.g. "Methods, paragraph 1"), since the page numbers of the final published paper may be different from the page numbers in the current manuscript.
Please find page numbers included in the consort checklist

15) Please add the following statement, or similar, to the Methods (for example line 160): "This study is reported as per the Consolidated Standards of Reporting Trials (CONSORT) guideline for randomised controlled trials (S1 Checklist)."

This has been updated

16) ll.431ff: Thank you for your response regarding causal versus associational language. Typically, trials are only powered for the primary outcome(s), which justifies the use of causal language. Unless you can demonstrate that the study was also powered for the secondary outcomes, we ask that you remove any causal language and instead use associational language when reporting the secondary outcomes. At the very least, please include a qualifier (e.g., "suggesting a potential causal link...") to mitigate the causal relationship.

We have now tempered the language in the following section:

Table 4 shows the association between the intervention and risky sexual behaviour and other secondary outcomes. The intervention was associated with a significant increase in the likelihood of stopping transactional sex by 8.6 percentage points (16.9% vs 25.5%) (marginal effect (ME) = 0.086 (95% CI [0.015,0.158]); p=0.018). There was an increase in condom use at last sex of about 15 percentage points as measured by the list experiment method that was not statistically significant at the 5% level (ME=0.152 (95% CI [-0.021,0.324]); p=0.085). We also observed a reduction in the number of sex acts by an average of 0.36 for women having transactional sex that was not statistically significant at the 5% level (ME=-0.358 (95% CI[-0.781,0.065]); p=0.097).

Among women and girls engaged in commercial sex, results were mixed. The intervention was not associated with a greater likelihood of leaving sex work or of engaging in protected sex. However, it was associated with very small increases in the likelihood of engaging in anal sex (ME=0.014 (95% CI [0.002,0.026]); p=0.024) and in engaging in sex with riskier clients (ME=0.037 (95% CI [-0.004,0.078]); p=0.075).

The study found that the intervention was not associated with the likelihood of experiencing physical violence, nor with participants' levels of loneliness and stress. Both unadjusted and adjusted odd ratios are presented in Appendix A9.
